# Potent Functional Antibody Responses Elicited by HIV-I DNA Priming and Boosting with Heterologous HIV-1 Recombinant MVA in Healthy Tanzanian Adults

**DOI:** 10.1371/journal.pone.0118486

**Published:** 2015-04-14

**Authors:** Agricola Joachim, Charlotta Nilsson, Said Aboud, Muhammad Bakari, Eligius F. Lyamuya, Merlin L. Robb, Mary A. Marovich, Patricia Earl, Bernard Moss, Christina Ochsenbauer, Britta Wahren, Fred Mhalu, Eric Sandström, Gunnel Biberfeld, Guido Ferrari, Victoria R. Polonis

**Affiliations:** 1 Department of Microbiology and Immunology, Muhimbili University of Health and Allied Sciences, Dar es Salaam, Tanzania; 2 Department of Microbiology, Tumor and Cell Biology, Karolinska Institutet, Stockholm, Sweden; 3 Public Health Agency of Sweden, Solna, Sweden; 4 Department of Laboratory Medicine, Karolinska Institutet, Huddinge, Sweden; 5 Department of Internal Medicine, Muhimbili University of Health and Allied Sciences, Dar es Salaam, Tanzania; 6 The Military HIV Research Program, The Henry M. Jackson Foundation for the Advancement of Military Medicine, Bethesda, Maryland, United States of America; 7 The Military HIV Research Program, Walter Reed Army Institute of Research, Silver Spring, Maryland, United States of America; 8 National Institute of Allergy and Infectious Diseases, National Institutes of Health, Bethesda, Maryland, United States of America; 9 Department of Medicine, University of Alabama at Birmingham, Birmingham, Alabama, United States of America; 10 Venhälsan, Karolinska Institutet at Södersjukhuset, Stockholm, Sweden; 11 Department of Surgery, Duke University Medical Center, Durham, North Carolina, United States of America; Harvard Medical School, UNITED STATES

## Abstract

Vaccine-induced HIV antibodies were evaluated in serum samples collected from healthy Tanzanian volunteers participating in a phase I/II placebo-controlled double blind trial using multi-clade, multigene HIV-DNA priming and recombinant modified vaccinia Ankara (HIV-MVA) virus boosting (HIVIS03). The HIV-DNA vaccine contained plasmids expressing HIV-1 gp160 subtypes A, B, C, Rev B, Gag A, B and RTmut B, and the recombinant HIV-MVA boost expressed CRF01_AE HIV-1 Env subtype E and Gag-Pol subtype A. While no neutralizing antibodies were detected using pseudoviruses in the TZM-bl cell assay, this prime-boost vaccination induced neutralizing antibodies in 83% of HIVIS03 vaccinees when a peripheral blood mononuclear cell (PBMC) assay using luciferase reporter-infectious molecular clones (LucR-IMC) was employed. The serum neutralizing activity was significantly (but not completely) reduced upon depletion of natural killer (NK) cells from PBMC (p=0.006), indicating a role for antibody-mediated Fcγ-receptor function. High levels of antibody-dependent cellular cytotoxicity (ADCC)-mediating antibodies against CRF01_AE and/or subtype B were subsequently demonstrated in 97% of the sera of vaccinees. The magnitude of ADCC-mediating antibodies against CM235 CRF01_AE IMC-infected cells correlated with neutralizing antibodies against CM235 in the IMC/PBMC assay. In conclusion, HIV-DNA priming, followed by two HIV-MVA boosts elicited potent ADCC responses in a high proportion of Tanzanian vaccinees. Our findings highlight the potential of HIV-DNA prime HIV-MVA boost vaccines for induction of functional antibody responses and suggest this vaccine regimen and ADCC studies as potentially important new avenues in HIV vaccine development.

**Trial Registration:**

Controlled-Trials ISRCTN90053831

The Pan African Clinical Trials Registry ATMR2009040001075080 (currently PACTR2009040001075080)

## Introduction

There is considerable evidence that neutralizing antibodies are important to protect from HIV-1 infection. In fact, passive immunization studies using HIV-1 neutralizing polyclonal and monoclonal antibodies against HIV-1, HIV-2, SIV or SHIV have been shown to provide various degrees of protective efficacies in primates [1]. HIV-specific monoclonal antibodies infused intravenously to macaques have been shown to protect against intravenous and mucosal chimeric SHIV challenge [2,3,4,5]. Neutralizing antibodies block HIV entry by recognizing epitopes on the envelope spike critical for their interaction with receptors and co-receptors, and/or play a role in the fusion process [6]. However, developing an effective HIV vaccine that elicits neutralizing antibodies against a wide range of primary HIV isolates remains a significant challenge [7].

Recently, antibodies with functional properties other than the classical cell-free virus neutralizing activity are being considered as potentially protective against HIV-1 [8]. Non-neutralizing antibodies have the ability to control HIV infection by binding to Fc receptors mediating anti-HIV activities, including antibody-dependent cellular cytotoxicity (ADCC) and antibody-dependent cell-mediated viral inhibition (ADCVI) [9]. These activities are primarily mediated by Fc-γ receptor IIIa (Fcγ-RIIIa) or CD16 expressed on the surface of monocytes/macrophages and natural killer (NK) cells. Antibodies binding the HIV-1 antigens expressed on the membrane of infected cells can also bind the Fcγ-RIIIa via their Fc region of immunoglobin G (IgG). The effector cells are then triggered to release cytokines such as IFN-γ and cytotoxic granules containing perforin and granzymes that specifically lyse the HIV-infected cells [10].

Unlike virus neutralizing antibodies, which neutralize and clear free virions from circulation, ADCC-mediating antibodies can kill the CD4^+^ cells targeted by HIV-1 at the time of virus entry [11] and/or at the time of virus budding, thus preventing infection and/or the cell-to-cell transmission of HIV-1 [12]. It has been reported that rhesus macaques vaccinated with replicating recombinant adenovirus type 5 followed by SIV gp120 developed potent ADCC antibody activity that significantly correlated with reduced acute viremia after a mucosal challenge with pathogenic SIV [13,14]. During persistent infection with live attenuated SIV, Env-specific ADCC activity developed and was associated with protection against pathogenic SIV challenge [15]. Increasing titers of gp120-specific ADCC-mediating antibodies have been shown to correlate inversely with the rates of HIV-1 disease progression, while rapid progressors had significantly lower titers of antibodies against HIV-1 gp120 compared to the non-rapid progressors [16]. Similarly, HIV-1 elite controllers with undetectable viremia had higher ADCC antibody titers than viremic individuals [17,18]. Recently, Wren et al. reported that HIV infected long-term slow progressors have broader epitope specific ADCC responses compared to progressors [19]. Additionally, ADCC activity in breast milk has been shown to be associated with reduced risk of mother-to-child transmission of HIV [20]. In a study of children born to HIV-infected mothers, higher ADCC antibody titers correlated with a better clinical stage of the children [21]. Lastly, it has been postulated that ADCC responses detected in the RV144 study might have played a role in the protection against HIV-1 infection in individuals with low levels of anti-Env IgA antibodies [22,23].

We have previously conducted a phase I/II HIV vaccine trial (HIVIS03) in Dar es Salaam, Tanzania, that included priming with HIV-DNA followed by boosting with HIV-MVA among healthy adult volunteers [24]. A high neutralizing antibody response rate (up to 83%) was demonstrated in the vaccinees using infectious molecular clones (IMC) in a peripheral blood mononuclear cell (PBMC) assay, but there was no neutralizing activity in the TZM-bl pseudovirus assay [24]. Other investigators have also reported contrasting data when using PBMC versus TZM-bl neutralization assays [25,26,27,28,29]. The IMC/PBMC assay used here is a platform where the antibody is continuously present. It has been reported that NK cells may influence the assessment of neutralization by HIV+ polyclonal sera under such conditions [30]. The aim of the current study was to determine the role of NK cells in the HIV inhibitory activity observed in PBMC assays using sera from the HIVIS03 vaccinated volunteers, and to further explore potential FcγR-mediated ADCC responses, as compared to neutralizing or binding antibody responses.

## Materials and Methods

### Ethics statement

The HIVIS03 trial protocol was approved by Tanzania’s National Health Research Ethics Committee and the Senate Research and Publications Committee of MUHAS. The protocol for the trial is available as supporting information; see S1 Protocol. Use of the vaccine candidate products for humans in Tanzania was approved by the Tanzania Food and Drugs Authority (TFDA). The HIVIS03 trial was conducted in accordance with the International Conference on Harmonization, Good Clinical Practice guidelines (ICH-GCP). All volunteers provided signed written informed consent. The trial was registered at the Pan African Clinical Trial with Registry ATMR2009040001075080 (currently PACTR2009040001075080) and Controlled-Trials registry number ISRCTN90053831.

### Study design

Serum samples were obtained from volunteers enrolled in the HIVIS03 trial which had been conducted among healthy individuals in Tanzania using priming with multi-clade, multi-gene HIV-DNA and boosting with HIV-MVA [24]. In the HIVIS03 trial, 60 HIV-uninfected volunteers were randomized into three groups of 20 volunteers and received placebo or 1 mg HIV-DNA intradermally (id) or 3.8 mg intramuscularly (im). DNA plasmids expressing HIV-1 gp160 subtypes A, B, C; Rev B; Gag A, B and RTmut B were given at months 0, 1 and 3 using a needle-free Biojector device. Recombinant MVA expressing CRF01_AE HIV-1 Env subtype E and Gag-Pol subtype A (HIV-MVA) was administered im by needle at months 9 and 21 [24]. Sera used in the present study had been collected from 29 vaccinees at baseline, eight weeks post-first and four weeks post-second HIV-MVA boosting and stored at -70°C until the time of testing.

### PBMC neutralization assay

A PBMC assay, employing IMC carrying the luciferase gene from *Renilla reneformis* (LucR) as a reporter, was used for measuring neutralization activity [31]. The PBMC were obtained by standard ficoll-hypaque gradient centrifugation and the mixed (bulk) PBMC were cryopreserved at 30 million per ml of freezing medium in 1 ml cryovials. The following IMCs were used: CM235 CRF01_AE, SF162 subtype B and BaL subtype B. IMC reporter virus (25 μl) was incubated at 37°C with 25 μl of diluted test serum from pre-and post-vaccination in triplicates in 96-well round bottom plates for 1 hour. For the bulk PBMC assays, phytohemagglutinin (PHA) stimulated PBMC (10^5^ cells/well in 50 μl) were added and plates were incubated overnight. One hundred μl of RPMI/IL-2 medium were added to each well on the next day and plates were incubated further for three days. Each well was then treated with 50 μl of lysis buffer, followed by two freeze/thaw cycles, and 20 μl of the cell lysate was transferred to a corresponding 96-well Perkin Elmer black opti-plate. A 100 μl aliquot of substrate was then added to each well via the injection system of the Envision Luminometer (Perkin Elmer Inc. USA), followed by immediate measurement of luminescence signal in relative luminescence units (RLU). The percent neutralization of the post-vaccination serum was calculated based on the level of virus growth in the presence of the same dilution of pre-vaccination serum and neutralization values greater than 50% were considered positive.

### IgG depletion from serum

IgG depletion was performed on whole vaccinee sera using protein G Sepharose beads (GE Healthcare Bio-Science Corp, USA) as per manufacturer’s instructions. Briefly, 200 μl of protein G Sepharose beads were mixed with 200 μl of phosphate buffered saline (PBS) and then added to 100 μl of sera diluted 1:5. The mixture was rotated slowly in a sample mixer (Invitrogen, Carlsbad, CA, USA) for 50 min at room temperature. The depleted IgG serum fractions were recovered by centrifugation three times for 2 min.

### NK cell depletion from PBMC

For the neutralization assays using NK cell- depleted PBMC, depletions were performed on cryopreserved PBMC using mouse anti-human CD16 and CD56 antibodies (Invitrogen, Carlsbad CA) and Dynabeads (M-280) coated with sheep anti-mouse IgG (Invitrogen, Carlsbad CA), as per the manufacturer’s instructions. The PBMC were then PHA stimulated and verification of NK cell depletion was performed using flow cytometry; >90% of NK cells were depleted.

### Infection of CEM.NKR_CCR5_ cell line with HIV-1 IMC

For ADCC assays, IMCs were titrated in order to achieve maximum expression within 36–48 hours post-infection as determined by detection of Luciferase activity and intra-cellular p24 expression and subsequently cryopreserved. We infected 1x10^6^ *CEM*.*NKR*_*CCR5*_ cells with 1 TCID50/cell IMC_CM235_ by incubation for 0.5 hour at 37°C and 5% CO_2_ in presence of DEAE-Dextran (7.5 μg/ml). The cells were subsequently re-suspended at 0.5x10^6^/ml and cultured for 36–48 hours in complete medium containing 7.5μg/ml DEAE-Dextran. For each ADCC assay, we monitored the frequency of infected target cells by intracellular p24 staining. Assays performed using the infected target cells were considered reliable if the percentage of viable p24^+^ target cells was ≥20% on the day of testing.

### ADCC-GranToxiLux (ADCC-GTL) assay

ADCC-mediating antibody was detected according to the previously described flow cytometry GTL based assay using gp120 coated target cells [32]. The CEM.NKR_CCR5_ target cells were coated with recombinant gp120 HIV-1 protein derived from Env of HIV-1 CM243 CRF01_AE (GenBank accession no. AY214109; Protein Sciences Corporation) or HIV-1 gp120 SF162 subtype B (GenBank accession no. AAT67508; Immune Technology Corp). Target cells were coated at a concentration of 20 μg/μl as previously described [33]. PBMCs obtained from an HIV-seronegative healthy donor were used as effectors. Effectors and target cells (E/T) were used at a ratio of 30:1. Cells were acquired with LSR II (BD Bioscience, USA). Data analysis was performed using FlowJo software version 9.5.3. The results were expressed as percentage of Granzyme B (GzB) activity positive cells. The final results were expressed after subtracting the background from the percent GzB activity observed under the conditions containing effector and target cell populations in the presence of vaccinee serum. The ADCC-mediating antibody titer was defined as the reciprocal of the highest dilution indicating a positive GzB response (>8% GzB activity) after background subtraction as described by Pollara et al [32].

### ADCC-luciferase assay

This ADCC assay was performed using Env.IMC.LucR virus-infected cells as targets as previously described [33]. The Env-IMC-LucR viruses used were subtype CRF01_AE HIV-CM235-2-LucR.T2A.ecto/293T(IMC_CM235_) (GenBank accession no. AF259954.1) and SF162.LucR.T2A.ecto/293T(IMC _SF162_) (GenBank accession no. EU123924), here referred to as CM235 IMC and SF162 IMC, respectively. Reporter viruses were produced by transfection of 293T/17 cells with proviral IMC plasmid DNA. Briefly, cryopreserved IMC-Infected CEM.NKR_CCR5_ target cells were thawed and rested by incubation for 2 hours at 37°C and 5% CO_2_ before utilizing them in the assay. PBMC effector and target cells were counted via Guava and the concentrations were adjusted to reach a final effector-to-target ratio of 30:1. In some experiments, fresh target cells were used directly without the 2 hours incubation. Twenty-five μl of effector/target cell suspension were incubated with appropriately diluted sera (at 1:50) in duplicate wells in a 96-well flat bottom plates for 30 min at room temperature. A preparation of polyclonal purified IgG from HIV infected donors (HIVIG- obtained through the AIDS Reagent Program, Division of AIDS, NIAID, NIH) was used as positive control while serum from an HIV-uninfected individual was used as negative control. The plates were centrifuged for 1 min at 300 ×g and incubated for 5.5 hours at 37°C and 5% CO_2_. After incubation, 50μl of diluted ViviRen substrate (Promega, diluted 1:500) were added to each well and incubated for 9 min at 37°C and 5% CO_2_. RLU were measured immediately using a luminometer (Perkin Elmer Inc.). ADCC activity was measured as the percent of loss of luciferase activity observed in the presence of serum. The ADCC-mediating antibody titer was defined as the reciprocal of the highest dilution indicating a positive specific killing (>15% specific killing activity determined based on the responses observed before immunization to allow for 2% false positive rate) after background subtraction.

### Assessment of binding antibodies

Binding antibody to recombinant HIV-1_96ZM651_ subtype C gp140 protein (kindly provided by the Centre for AIDS Reagents, NIBSC Potter Bar, UK) was determined using an enzyme-linked immunosorbent assay (ELISA) as previously described [34]. Binding antibody to recombinant HIV-1_CM243_ CRF01_AE gp120 (Protein Science Corp) was performed as follows. Microtiter plates (Nunc) were coated with 25μg/ml of recombinant HIV-1_CM243_ gp120 (pH 7.5) diluted in phosphate carbonate buffer and incubated overnight at 4–8°C. Plates were washed and blocked with 1% Tween 20 in PBS. Diluted sera from vaccinees were added to the plates in duplicate wells at 1:50 starting dilutions and incubated for 1 hour at 37°C, along with positive and negative controls on each plate. Goat anti-human IgG conjugated to horseradish-peroxidase (KPL) was added and incubated at 37°C for 1hour. Appropriate substrate was used to develop the plates for 30 min and the reaction was stopped using 2N sulfuric acid. The optical density (O.D.) for each well was then read at 450 nm. Endpoint titers were defined as the reciprocal of the highest serum dilution that conferred an O.D. value greater than the negative control cut-off, defined as twice the mean O.D. for the negative controls, plus 2 standard deviations.

### Statistical analysis

The Wilcoxon rank test was used to determine statistical significance between bulk and NK cell depleted PBMCs. Neutralizing antibody titers were defined as 50% inhibitory doses (ID_50_) >20. The Mann-Whitney test was used to compare the magnitudes of ADCC responses, neutralizing titers and binding antibody titers between the vaccination groups. The Fisher’s Exact test was used to compare the differences in ADCC response rates against specific viruses. Correlations were determined by the Spearman rank correlation method used for non-parametric data. A two-sided p-value of ≤0.05 was considered statistically significant.

## Results

### Magnitude of neutralizing antibody responses

**Fig 1:**
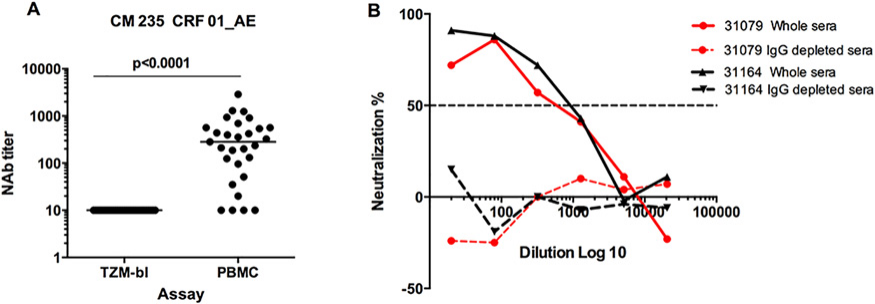
Neutralizing antibody responses. Neutralizing antibody responses in vaccinees four weeks after the second HIV-MVA boost vaccination as determined using the pseudovirus/TZMbl assay versus the IMC/PBMC assay (A). The neutralizing antibody activity detected by the IMC/PBMC assay was IgG-mediated (B). The solid lines indicate neutralization of the CM235 CRF01_AE IMC by unfractionated sera from two different volunteers, while the dashed lines indicate the percent neutralization by the same sera after depletion of IgG. For graphing, serum samples with no neutralizing activity at 1:20 dilutions were arbitrarily assigned a value of 10.

In the phase I/II HIV vaccine trial (HIVIS03) we reported high neutralizing antibody response rates in vaccinees after receipt of three HIV-DNA and two HIV-MVA vaccinations using the IMC/PBMC assay. The response rates were highest against the CM235 CRF01_AE virus (24/29, 83%), followed by the SF162 subtype B virus (21/29, 72%) and the BaL subtype B virus (9/29, 31%) [24]. In the present study, we calculated titers to measure the magnitude of neutralizing antibodies against the CM235 IMC. Thus, while there was no neutralizing activity demonstrated in the pseudovirus/TZM-bl assay (all ID_50_s <20), significant titers were measured four weeks after the second HIV-MVA vaccination when using the IMC/PBMC assay. The ID_50_s among responders in the IMC/PBMC assay ranged from 20 to 2868, with a median titer of 357, as shown in Fig 1A. The neutralizing antibody titers did not differ significantly between id primed (median 421, range 20–1289) and im primed (median 259, range 96–2886) vaccinees (p = 0.67 by Mann-Whitney test) (Fig S2A in S1 File).

### Neutralizing activity was IgG mediated

To verify that the HIV neutralizing activity was IgG–mediated, we tested whole sera from two individuals with neutralizing antibody titers of more than 1000 against CM235 IMC with and without IgG depletion. The neutralizing activity was removed (<50% neutralization at all serum dilutions) in the two IgG-depleted sera (Fig 1B), confirming that the neutralizing activity detected in the IMC/PBMC assay against the CM235 IMC was IgG-mediated.

### Role of Natural Killer (NK) cells in PBMC neutralizing antibody assay activity

**Fig 2:**
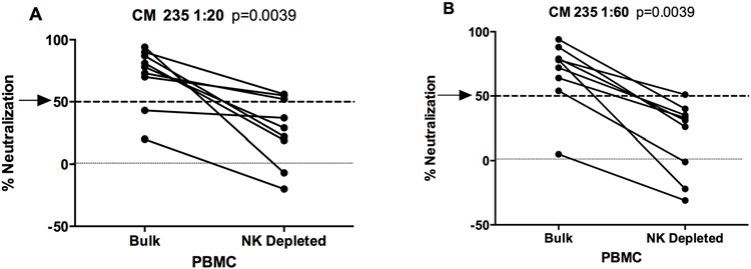
Impact of NK cell depletion on neutralizing antibody activity detected by the IMC/PBMC assay. NK cell depletion reduces neutralizing antibody activity against CM235 at 1:20 (A) and 1:60 (B) serum dilutions, as measured using the IMC/PBMC neutralization assay. The black lines connect the neutralizing activities by sera from the same vaccinees before and after NK cell depletion.

Given that the TZM-bl pseudovirus entry assay demonstrated no neutralizing activity and taking into consideration that the PBMC assay utilizes a mixture of cells, we sought to better understand the functional antibody activity observed in the PBMC assay, where antibody remains present with the cells and virus throughout the entire 4 day culture period allowing other effector cells present to exert potential functional activity [30], and where multiple rounds of infection may occur. To explore the possible role of NK effector cells in these neutralization assays using vaccinee sera, we tested the neutralizing activity of sera from HIVIS03 vaccinees against the CM235 CRF01_AE and BaL subtype B IMC using bulk and NK cell-depleted PBMC as targets in the neutralization assay. Sera from nine vaccinees collected four weeks after the second HIV-MVA boost who had neutralizing antibody titers above 200, were tested at 1:20 and 1:60 dilutions. NK cell depletion resulted in a significant decrease, but not a complete loss of, neutralizing activity against the CM235 IMC when using sera diluted at 1:20 (Fig 2A, p = 0.0039) and at 1:60 (Fig 2B, p = 0.0039), indicating a possible role for antibody-mediated Fcγ-receptor function. At a serum dilution of 1:20, some residual activity was still present against CM235 virus when NK cell-depleted PBMC were used, indicating that, for certain volunteers, some of the neutralization was not dependent on NK cells. When sera from the same 9 volunteers were similarly tested against the BaL subtype B IMC, neutralization was significantly reduced at both 1:20 (p = 0.003, Fig S1A in S1 File) and at 1:60 (p = 0.003, Fig S1B in S1 File), and no neutralization >50% was observed when using NK-depleted PBMC.

### ADCC-mediating antibody responses

Having demonstrated a role for NK effector cells in the PBMC assay, we next assessed whether ADCC antibody responses were elicited by the HIVIS03 vaccine regimen. Serum samples collected four weeks after the second HIV-MVA vaccination were tested against the vaccine homologous CRF01_AE (Env E) and subtype B viruses. Sera from the 29 vaccinees were examined for ADCC activity against gp120-coated CEM.NKR_CCR5_ target cells using the flow cytometric GTL assay and/or against IMC-infected CEM.NKR_CCR5_ target cells using the ADCC-luciferase assay. The serum samples from one volunteer were only available in sufficient volume for ADCC testing against the CM235 IMC-infected target cells. Sera were tested at baseline, two months after the first HIV-MVA boost and four weeks after the second HIV-MVA boost. There was no detectable ADCC activity pre-vaccination and two months after the first HIV-MVA boost in either of the two ADCC assays. The ADCC antibody response rates four weeks after the second HIV-MVA boost are summarized in Table 1. Twenty-one out of 28 (75%) vaccinees had detectable ADCC-GTL activity against SF162 gp120-coated targets (median titer 876, range 296–5261) and 24/28 (86%) against CM243 gp120-coated target cells (median titer 1841, range 146–7327). ADCC responses against SF162 subtype B infected cells were detected in 19/28 (68%, median titer 715, range 365–4460) volunteers tested, and against CM235 CRF01_AE infected cells in 28/29 (97%, median titer 1076, range 200–24668) vaccinees in the ADCC-luciferase assay (Table 1).

**Table 1:** ADCC-mediating antibody response rates in 29 vaccinees primed with HIV-DNA and tested four weeks after the second HIV-MVA vaccination.

Assay	Target cells	Virus	Subtype	Positive/ Total responders (%)			P-value (Fisher’s Exact test Id vs im
				Overall	Id	Im	
ADCC-GTL	CEM.NKR _CCR5_ coated gp120	SF162	B	21/28 (75)	13/16(81)	8/12(67)	0.41
ADCC-GTL	CEM.NKR _CCR5_ coated gp120	CM 243	CRF01_AE	24/28(86)	13/16 (81)	11/12 (92)	0.61
ADCC-Luciferase	IMC-LucR infected CEM.NKR_CCR5_	SF162	B	19/28(68)	13/16(81)	6/12(50)	0.11
ADCC-Luciferase	MC-LucR infected CEM.NKR_CCR5_	CM 235	CRF01_AE	28/29(97)	15/16 (94)	13/13(100)	1.00

ADCC-GTL: Flow cytometric antibody-dependent cellular-cytotoxicity Gran Toxi Lux-based assay

**Fig 3:**
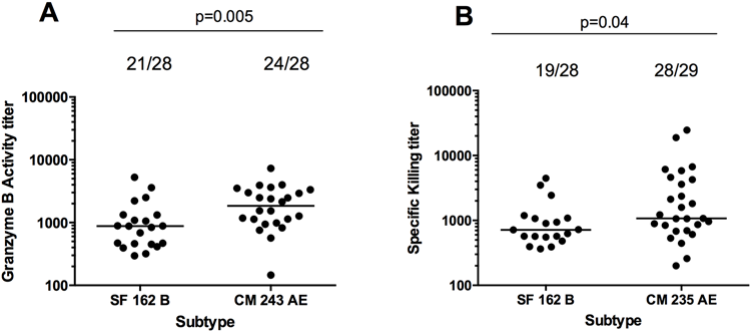
Magnitude of ADCC responses using IMC-infected and gp120-coated targets. The magnitude of the ADCC responses against subtype B (SF162) and CRF01_AE (CM235) targets using gp120-coated targets in the ADCC-GTL assay (A) or using IMC-infected targets in the ADCC-Luciferase assay (B) is shown. In both assays, the CRF01_AE ADCC response was the most potent.

**Fig 4:**
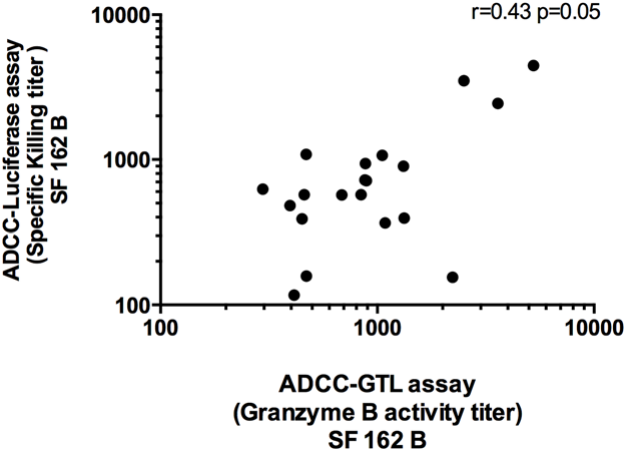
Positive correlation of ADCC responses between the ADCC-Luciferase assay and the ADCC-GTL assay. The Spearman rank correlation between ADCC responses to SF162 subtypes B as measured by the ADCC-Luciferase assay versus the ADCC-GTL assay is shown.

The ADCC mediating antibody responses against CRF01_AE were significantly higher in magnitude than those against subtype B, when detected using either the ADCC-GTL assay (p = 0.0057) or the ADCC-luciferase assay (p = 0.041) (Fig 3). ADCC antibody responses to SF162 were of the same magnitude irrespective of whether the ADCC-GTL assay or the ADCC luciferase assay was used (p = 0.9784). As shown in Fig 4, a correlation of the ADCC antibody titers was observed between the two assays when using subtype B SF162 targets (r = 0.43, p = 0.05). Similarly, the ADCC antibody responses against CM243 CRF01_AE gp120 coated cells in the ADCC-GTL assay and CM235 infected cells in the ADCC-luciferase assay were of the same magnitude (p = 0.479). However, a correlation between the ADCC antibody titers in the two assays was not noted for CM235 (r = 0.13, p = 0.48, Fig S3 in S1 File) Several of the subjects exhibited cross-clade ADCC; 18/28 (64%) had reactivity to both SF162 subtype B and CM235 CRF01_AE when using infected target cells, and 19/28 (68%) exhibited reactivity to both SF162 and CM243 when using gp120-coated targets. Furthermore, ADCC antibody titers did not differ significantly between the id and im primed vaccinees in any assay (Table 1 and Fig S2, B-C in S1 File).

### ADCC activity against CM235 IMC correlated with neutralizing activity

**Fig 5:**
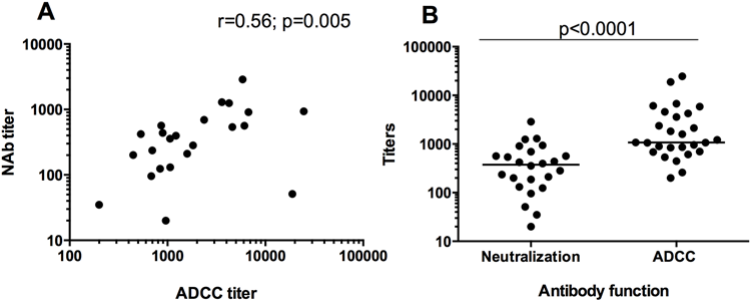
Comparison of neutralizing antibody responses to CM235 in the IMC/PBMC assay and ADCC responses to CM235 in the ADCC-Luciferase assay. The neutralizing antibody and ADCC titers showed a positive correlation (A). The CM235 CRF01_AE titers in the ADCC-Luciferase assay were significantly higher than the CM235 CRF01_AE titers measured in the PBMC/IMC neutralizing antibody assay (B, p<0.0001).

Both ADCC-mediating antibodies and PBMC neutralizing antibody activity against CM235 CRF01_AE IMC were detected in 24/29 (79%) vaccinees four weeks after the HIV-MVA boost. Additionally, four vaccinees had ADCC mediating antibody titers of 260 to 2125 against CM235 CRF01_AE using infected cells, but no demonstrable neutralization in the IMC/PBMC neutralization assay. Only one vaccinee was negative in the ADCC assay, but had a detectable neutralizing antibody titer of 187. Titers of ADCC antibodies against CM235 IMC-infected cells were directly correlated with neutralizing antibodies against CM235 IMC in the PBMC assay (r = 0.56 p = 0.005) (Fig 5A). The potency of the HIV-MVA vaccine-homologous ADCC response to CM235 was significantly higher (median 1076, range 200–24668) than the activity measured in the IMC/PBMC neutralization assay (median 377, range 20–2868) (P<0.0001, Wilcoxon matched signed test) (Fig 5B).

### Comparison of anti-Env binding antibodies and functional antibody responses

**Fig 6:**
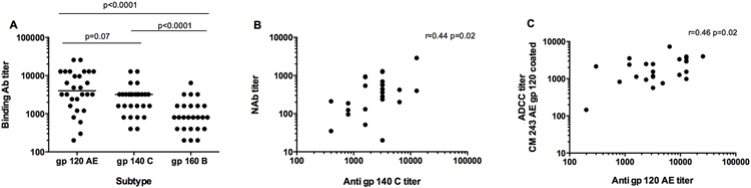
Functional and binding antibody responses in vaccinees tested four weeks after the second HIV-MVA boost vaccination. Binding antibody titers to CRF01_AE (CM243) gp120, subtype C (HIV-1_96ZM651_) gp140 or subtype B (IIIb) gp160 envelope protein (A). The response rate was 100% to CRF01_AE gp120 and subtype C gp140, while 26/29 (90%) vaccinees exhibited binding antibodies to subtype B gp160. There were positive correlations between: IMC/PBMC neutralizing antibody titers to CM235 and subtype C gp140 antibody binding titers (B, p = 0.02); ADCC responses to subtype CM243 CRF01_AE determined by the ADCC-GTL assay and CM243 CRF01_AE gp120 binding antibodies (C, p = 0.02).

Anti-Env binding antibody was assessed by ELISA in sera collected from 29 vaccinees at baseline and four weeks after the second HIV-MVA vaccination. All 29 (100%) vaccinees had detectable antibodies to recombinant CM243 CRF01_AE gp120 (median titer 3200, range 200–25600) and HIV-1 subtype C gp140 (median titer 3200, range 400–12800) four weeks after the second HIV-MVA boost. As shown in Fig 6, the binding antibody titers against CRF01_AE gp120 and subtype C gp140 were significantly higher than those we previously reported [24] against subtype B gp160 (median titer 800, range 200–6400), p<0.0001 and p<0.0001, respectively (Mann Whitney test, Fig 6A). There were significant correlations between neutralizing antibody titers against the CM235 IMC and the ELISA titers to subtype C gp140 (r = 0.45 p = 0.02) as shown in Fig 6B. ADCC antibody titers against CM243 gp120 coated target cells also correlated positively with CM243 gp120 binding antibodies (r = 0.46, p = 0.02, Fig 6C). All other comparisons yielded non-significant p-values (data not shown). In addition, no difference in binding antibodies to CRF01_AE gp120 was observed between vaccinees obtaining id or im immunization with the HIV-DNA (Fig S2, D in the S1 File).

## Discussion

In the present study, we investigated the antibody responses of HIVIS03 vaccinees primed with HIV-DNA containing plasmids expressing HIV Envelope subtypes A, B and C and boosted with HIV-MVA (containing genes expressing Env subtype CRF01_AE and Gag-Pol subtype A). We found that a majority of the vaccine recipients had ADCC antibody responses, 68% against subtype B (present in the prime) and 97% against CRF01_AE (present in the boost) four weeks after the second HIV-MVA vaccination. We had previously observed neutralizing antibodies using an IMC/PBMC assay in the same HIVIS03 sera tested four weeks after the second HIV-MVA vaccination, where up to 83% of the vaccinees exhibited neutralizing activity to CM235 CRF01_AE virus [24]. Here, we demonstrate that the magnitude of ADCC-mediating antibody correlated well with neutralizing activity against the vaccine-homologous CM235 CRF01_AE IMC. Furthermore, NK cell depletion from PBMC targets abrogated the neutralizing activity (but did not completely abolish the activity in all volunteers) in the PBMC assay, indicating a role for Fc-receptor mediated antibody functions, as well as some possible additional neutralizing function(s). Brown et al. have previously demonstrated that NK cells were critical for a substantial portion of the neutralizing activity of HIV+ sera detected when using the IMC/PBMC assay [30]. In our study, the residual neutralizing activity detected after NK cell depletion in the IMC/PBMC assay may have been due to either Fab-dependent neutralizing activity, or Fc-dependent antiviral activity mediated by other cells in bulk PBMC, such as monocytes/macrophages, that express surface Fc receptors. In future studies, it might be interesting to test for the presence of antibody-dependent cellular phagocytosis, direct NK cell-mediated killing, or other antibody-dependent cell-mediated virus inhibition mechanisms, using select sera from our study.

This is the first demonstration using human vaccinee sera, where HIV neutralizing activity was detected in an assay employing PBMC targets, while detecting no activity in a pseudovirus HIV entry inhibition assay. Data that are discordant in the TZM-bl cell versus PBMC assays have been previously reported for human monoclonal antibodies [25] and for HIV positive sera [28,29]. It has thus been suggested that for HIV vaccine evaluation, a battery of different functional antibody assay approaches should be applied until an antibody-based correlate of immune protection is identified [28,29].

Here, we report high titers of ADCC-mediating antibodies induced by HIV-DNA priming and HIV-MVA boosting in healthy volunteers, with a delay of 11–16 months preceding the second HIV-MVA boost. No difference was seen between vaccinees receiving three priming immunizations with a 3.8 mg dose of HIV-DNA im versus a 1 mg dose of HIV-DNA id prior to the HIV-MVA boosting.

We observed a dichotomy between neutralizing and non-neutralizing antibody responses, because the NAb were not detected in standard TZM-bl neutralization assays, whereas we observed a high (>50%) ADCC response rate. This is different than what was observed in the RV144 clinical trial where a protein subunit boost induced modest, but detectable NAb responses that did not correlate with lower risk of infection. In the efficacy trial conducted in Thailand, priming with a recombinant canarypox vector vaccine (ALVAC-HIV) and boosting with recombinant gp120 subunit vaccine (AIDSVAX B/E) induced ADCC activity that was associated with a reduced risk of infection in a subgroup of individuals with low serum IgA Env-specific antibodies [22,35]. Prime/boost vaccine concepts in non-human primate studies have previously been shown to induce ADCC-mediating antibodies [13,14,36,37]. Furthermore, in rhesus macaque vaccine studies, ADCC responses were reported to correlate with reduced acute viremia after intrarectal SIVmac251 challenge [13,14] or intravenous SHIV89.6P challenge [37].

We characterized the ADCC activity against tier 1 viruses, including the CM235 CRF01_AE virus and the SF162 subtype B virus. At the time these analyses were performed, subtypes A and C LucR-IMC were not available for use in the ADCC or PBMC neutralization assays. There was no demonstrable neutralizing activity in the TZM-bl pseudovirus assay using BaL subtype B, GS015 subtype C and CM235 CRF01 AE pseudoviruses [24]. ADCC antibody responses were demonstrated using both gp120-coated (ADCC-GTL assay) and IMC-infected target cells (ADCC-luciferase assay) in samples collected four weeks after the second HIV-MVA vaccination. The ADCC antibody response rates were higher to CRF01_AE virus, (86% in the ADCC-GTL assay and 97% in the ADCC-luciferase assay) than to subtype B virus (75% in the ADCC-GTL assay and 68% in the ADCC-Luciferase assay). Similarly, using an assay employing ^51^Cr labeled CEM.NKR cells coated with either CRF01_AE gp120 or subtype B gp120 as target cells and PBMC as effector cells, a 40% ADCC antibody response rate to CRF01_AE and 30% response rate to subtype B was reported in vaccinees after receipt of three doses of 10^8^pfu MVA-CMDR [38]. In the Phase I/II RV135 clinical trial, a combination of ALVAC-HIV (vCP1521) prime and AIDSVAX B/E gp120 boost generated more HIV-specific ADCC activity in vaccinees, as compared to ALVAC-HIV alone. While we observed stronger and more frequent humoral responses to CRF01_AE in the present study, ADCC activity in RV135 sera tended to be slightly higher against the MN subtype B gp120, as compared to ADCC activity against the CM243 CRF01_AE gp120, but the difference was not statistically significant [39]. This observation is not surprising, as the protein boost in RV135 contained a MN subtype B gp120, while our HIV-MVA used as a boost contained only the CM235 CRF01_AE subtype E Env.

All vaccinees in the present study produced cross-clade binding antibodies to subtype C and CRF01_AE envelopes. Anti-CM243 gp120 CRF01_AE binding correlated with CRF01_AE ADCC antibody activity determined by ADCC-GTL (using matched CM243 gp120 coated targets), but not with ADCC antibody activity detected using CM235 IMC-infected target cells (ADCC-luciferase assay). Minor differences in gp120-specific ADCC epitopes between the CM235 and CM243 strains could account for the lack of correlation, or more likely, a contribution of ADCC responses directed against gp41 epitopes in the IMC-infected cell targets could play a role. Subtype C ADCC antibody activity was not tested for, but cross-clade gp140 subtype C binding antibodies were detected. Taken together, these findings suggest that antibodies mediating ADCC, and possibly neutralization, constitute subsets of antibodies detected in the ELISA, as would be expected. A similar correlation between ADCC responses and gp140 binding antibodies has been reported in SIV infection [40].

In summary, we have found that the HIV-DNA prime, recombinant HIV-MVA boost vaccine elicited potent ADCC mediating antibody responses in a high proportion (up to 97%) of the vaccinees four weeks after a second HIV-MVA vaccination. The NK cells were responsible for the majority of the inhibitory activity demonstrated by the IMC/PBMC assay, thereby supporting the observation of potent ADCC responses. Our findings highlight the potential of HIV-DNA prime, HIV-MVA boost vaccines for induction of non-neutralizing functional antibody responses and suggest this vaccine regimen and ADCC studies as a potential new avenue in HIV vaccine development.

## Supporting Information

S1 File **Fig S1. NK cell depletion from PBMC influenced the HIV-1 neutralizing antibody activity**. NK cell depletion from PBMC influenced the HIV-1neutralizing antibody activity to subtype B HIV-1 BaL at two different serum dilutions 1:20 (A) and 1:60 (B), as measured using the IMC/PBMC neutralization assay. The black lines connect the neutralizing activity from same vaccinees’ sera before and after NK cell depletion. **Fig S2. Antibody responses against CRF01_AE based on the route of HIV-MVA vaccination**. Comparison of antibody responses against CRF01_AE based on the route of HIV-MVA vaccination (id versus im), as determined using the IMC/PBM neutralization assay (A), the ADCC-Luciferase assay (B), the ADCC-GTL assay (C) and ELISA binding titers (D). Sera with no activity or negative values in the assays were arbitrarily assigned a reciprocal titer of 10. **Fig S3. Spearman rank correlation of ADCC responses**. No correlation was seen between ADCC responses to CM235 CRF01_AE as measured by the ADCC-Luciferase assay and ADCC responses to CM243 CRF01_AE as measured by the ADCC-GTL assay
(DOCX)

S1 Protocol Clinical Study Protocol HIVIS03. A Phase I/II trial to assess the safety and immunogenicity of a plasmid DNA-MVA prime boost HIV-1 vaccine candidate among volunteers in Dar es Salaam, Tanzania
(PDF)

## References

[1] Ferrantelli F, Ruprecht RM (2002) Neutralizing antibodies against HIV-back in the major leagues? Curr Opin Immunol 14:495–502. 12088685 10.1016/s0952-7915(02)00362-x

[2] Mascola JR, Lewis MG, Stiegler G, Harris D, VanCott TC, et al (1999) Protection of macaques against pathogenic simian/human immunodeficiency virus 89.6PD by passive transfer of neutralizing antibodies. J Virol 73:4009–4018. 10196297 10.1128/jvi.73.5.4009-4018.1999 PMC104180

[3] Mascola JR, Stiegler G, VanCott TC, Katinger H, Carpenter CB, et al (2000) Protection of macaques against vaginal transmission of a pathogenic HIV- 1/SIV chimeric virus by passive infusion of neutralizing antibodies. Nat Med 6:207–210. 10655111 10.1038/72318

[4] Nishimura Y, Igarashi T, Haigwood NL, Sadjadpour R, Donau OK, et al (2003) Transfer of neutralizing IgG to macaques 6 h but not 24 h after SHIV infection confers sterilizing protection: implications for HIV-1 vaccine development. Proc Natl Acad Sci USA 100:15131–15136. 14627745 10.1073/pnas.2436476100 PMC299920

[5] Hessell AJ, Rakasz EG, Tehrani DN, Huber M, Weisgrau KL, et al (2010) Broadly neutralizing monoclonal antibodies 2F5 and 4E10 directed against the human immunodeficiency virus type 1 gp41 membrane-proximal region protect against mucosal challenge by simian-human immunodeficiency virus SHIV_Ba-L_ . J Virol 84:1302–1313. 10.1128/JVI.01272-09 19906907 PMC2812338

[6] McElrath MJ, Haynes BF (2010) Induction of immunity to human immunodeficiency virus type 1 by vaccination. Immunity 33:342–355. 10.1016/j.immuni.2010.09.011 PMC3031162 21029964

[7] Hoxie JA (2010) Towards an antibody-based HIV-1 vaccine. Annu Rev Med 61:135–152. 10.1146/annurev.med.60.042507.164323 19824826

[8] Tomaras GD, Haynes BF (2010) Strategies for eliciting HIV-1 inhibitory antibodies. Curr Opin HIV AIDS 5:421–427. 10.1097/COH.0b013e32833d2d45 20978384 PMC3516814

[9] Robinson HL (2013) Non-neutralizing antibodies in prevention of HIV infection. Expert Opin Biol Ther 13:197–207. 10.1517/14712598.2012.743527 23130709

[10] Huber M, Trkola A (2007) Humoral immunity to HIV-1: neutralization and beyond. J Intern Med 262:5–25. 17598812 10.1111/j.1365-2796.2007.01819.x

[11] Guan Y, Pazgier M, Sajadi MM, Kamin-Lewis R, Al-Darmarki S, et al (2012) Diverse specificity and effector function among human antibodies to HIV-1 envelope glycoprotein epitopes exposed by CD4 binding. Proc Natl Acad Sci USA E69–E78. 10.1073/pnas.1217609110 23237851 PMC3538257

[12] Ahmad R, Sindhu ST, Toma E, Morisset R, Vincelette J, et al (2001) Evidence for a correlation between antibody dependent cellular cytotoxicity-mediating anti-HIV-1 antibodies and prognostic predictors of HIV infection. J Clin Immunol 21:227–233. 11403230 10.1023/a:1011087132180

[13] Gómez-Román VR, Patterson LJ, Venzon D, Liewehr D, Aldrich K, et al (2005) Vaccine-elicited antibodies mediate antibody-dependent cellular cytotoxicity correlated with significantly reduced acute viremia in rhesus macaques challenged with SIVmac251. J Immunol 174:2185–2189. 15699150 10.4049/jimmunol.174.4.2185

[14] Hidajat R, Xiao P, Zhou Q, Venzon D, Summers LE, et al (2009) Correlation of vaccine-elicited systemic and mucosal non-neutralizing antibody activities with reduced acute viremia following intrarectal simian immunodeficiency virus SIVmac251 challenge of rhesus macaques. J Virol 83:791–801. 10.1128/JVI.01672-08 18971271 PMC2612365

[15] Alpert MD, Harvey JD, Lauer WA, Reeves RK, Piatak M Jr, et al (2012) ADCC develops over time during persistent infection with live-attenuated SIV and is associated with complete protection against SIVmac251 challenge. PLoS Pathog 8:e1002890 10.1371/journal.ppat.1002890 22927823 PMC3426556

[16] Baum LL, Cassutt KJ, Knigge K, Khattri R, Margolick J, et al (1996) HIV-1 gp120 specific antibody-dependent cell mediated cytotoxicity correlates with rate of disease progression. J Immunol 157:2168–2173. 8757343

[17] Lambotte O, Ferrari G, Moog C, Yates NL, Liao H, et al (2009) Heterogeneous neutralizing antibody and antibody-dependent cell cytotoxicity responses in HIV-1 elite controllers. AIDS 23:897–906. 10.1097/QAD.0b013e328329f97d 19414990 PMC3652655

[18] Lambotte O, Pollara J, Boufassa F, Moog C, Venet A, et al (2013) High antibody-dependent cellular cytotoxicity responses are correlated with strong CD8 T cell viral suppressive activity but not with B57 status in HIV-1 elite controllers. PLoS One 8 e74855 10.1371/journal.pone.0074855.t002 24086385 PMC3781132

[19] Wren LH, Chung AW, Isitman G, Kelleher AD, Parsons MS, et al (2012) Specific antibody-dependent cellular cytotoxicity responses associated with slow progression of HIV infection. Immunology 138:116–123. 10.1111/imm.12016 PMC3575764 23173935

[20] Mabuka J, Nduati R, Odem-Davis K, Peterson D, Overbaugh J (2012) HIV-specific antibodies capable of ADCC are common in breast milk and are associated with reduced risk of transmission in women with high viral loads. PLoS Pathog 8(6):e1002739 doi: 0.1371/journal.ppat.1002739 22719248 10.1371/journal.ppat.1002739 PMC3375288

[21] Ljunggren K, Moschese V, Broliden PA, Giaquinto C, Quinti I, et al (1990) Antibodies mediating cellular cytotoxicity and neutralization correlate with a better clinical stage in children born to human immunodeficiency virus-infected mothers. J Infect Dis 161:198–202. 2299204 10.1093/infdis/161.2.198

[22] Haynes BF, Gilbert PB, McElrath MJ, Zolla-Pazner S, Tomaras GD, et al (2012) Immune-correlates analysis of an HIV-1 vaccine efficacy trial. N Engl J Med 366:1275–1286. 10.1056/NEJMoa1113425 22475592 PMC3371689

[23] Tomaras GD, Ferrari G, Shen X, Alam SM, Liao H-X, et al (2013) Vaccine-induced plasma IgA specific for the C1 region of the HIV-1 envelope blocks binding and effector function of IgG. Proc Natl Acad Sci USA 110:9019–9024. 10.1073/pnas.1301456110 23661056 PMC3670311

[24] Bakari M, Aboud S, Nilsson C, Francis J, Buma D, et al (2011) Broad and potent immune responses to a low dose intradermal HIV-1 DNA boosted with HIV-1 recombinant MVA among healthy adults in Tanzania. Vaccine 29:8417–8428. 10.1016/j.vaccine.2011.08.001 21864626 PMC4795940

[25] Binley JM, Wrin T, Korber B, Zwick MB, Wang M, et al (2004) Comprehensive cross-subtype neutralization analysis of a panel of anti-human immunodeficiency virus type 1monoclonal antibodies. J Virol 78:13232–52. 15542675 10.1128/JVI.78.23.13232-13252.2004 PMC524984

[26] Choudry V, Zang M-Y, Sidorov IA, Louis JM, Harris I, et al (2007) Cross-reactive HIV-1 neutralizing monoclonal antibodies selected by screening of an immune human phage library against an envelope glycoprotein (gp140) isolated from a patient (R2) with broadly neutralizing antibodies. Virology 363:79–90. 17306322 10.1016/j.virol.2007.01.015 PMC2696119

[27] Brown BK, Wieczorek L, Sanders-Buell E, Rosa Borges A, Robb ML, et al (2008) Cross-clade neutralization patterns among HIV-1 strains from the six major clades of the pandemic evaluated and compared in two different models. Virology 375:529–538. 10.1016/j.virol.2008.02.022 18433824

[28] Polonis VR, Brown BK, Rosa Borges A, Zolla-Pazner S, Dimitrov DS, et al (2008) Recent advances in the characterization of HIV-1 neutralization assays for standardized evaluation of the antibody response to infection and vaccination. Virology 375:315–320. 10.1016/j.virol.2008.02.007 18367229

[29] Fenyö EM, Heath A, Dispinseri S, Holmes H, Lusso P, et al (2009) International network for comparison of HIV neutralization assays: The Neut Net report. PLoS One 4: e4505 10.1371/journal.pone.0004505 19229336 PMC2640999

[30] Brown BK, Wieczorek L, Kijak G, Lombardi K, Currier J, et al (2012) The role of natural killer (NK) cells and NK cell receptor polymorphisms in the assessment of HIV-1 neutralization. PLoS One 7(4): e29454 10.1371/journal.pone.0029454 22509241 PMC3324450

[31] Edmonds TG, Ding H, Yuan X, Wei Q, Smith KS, et al (2010) Replication competent molecular clones of HIV-1 expressing Renilla luciferase facilitate the analysis of antibody inhibition in PBMC. Virology 408:1–13. 10.1016/j.virol.2010.08.028 20863545 PMC2993081

[32] Pollara J, Hart L, Brewer F, Pickeral J, Packard BZ, et al (2011) High-throughput quantitative analysis of HIV-1 and SIV-specific ADCC-mediating antibody responses. Cytometry A 79:603–612. 10.1002/cyto.a.21084 21735545 PMC3692008

[33] Pollara J, Bonsignori M, Moody MA, Liu P, Alam SM, et al (2014) HIV-1 Vaccine-Induced C1 and V2 Env-Specific Antibodies Synergize for Increased Antiviral Activities J Virol 88:7715–7726. 10.1128/JVI.00156-14 24807721 PMC4097802

[34] Nilsson C, Godoy-Ramirez K, Hejdeman B, Bråve A, Gudmundsdotter L, et al (2014) Broad and potent cellular and humoral immune responses after a second late HIV-MVA vaccination in HIV-DNA primed and HIV-MVA boosted Swedish vaccinees. AIDS Res Hum Retroviruses 30:299–311. 10.1089/AID.2013.0149 24090081 PMC3938943

[35] Rerks-Ngarm S, Pitisuttithum P, Nitayaphan S, Kaewkungwal J, Chiu J, et al (2009) Vaccination with ALVAC and AIDSVAX to prevent HIV-1 infection in Thailand. N Engl J Med 361: 2209–2220. 10.1056/NEJMoa0908492 19843557

[36] Florese RH, Demberg T, Xiao P, Kuller L, Larsen K, et al (2009) Contribution of non-neutralizing vaccine-elicited antibody activities to improved protective efficacy in rhesus macaques immunized with Tat/Env compared with multigenic vaccine. J Immunol. 182:3718–3727 10.4049/jimmunol.0803115 19265150 PMC2744397

[37] Xiao P, Zhao J, Patterson J, Brocca-Cofano E, Venzon D, et al (2010) Multiple vaccine-elicited nonneutralizing anti envelope antibody activities contribute to protective efficacy by reducing both acute and chronic viremia following simian/human immunodeficiency virus SHIV89.6P challenge in rhesus macaques. J Virol 84: 7161–7173. 10.1128/JVI.00410-10 20444898 PMC2898229

[38] Currier JR, Ngauy V, de Souza MS, Ratto-Kim S, Cox JH, et al (2010) Phase I safety and immunogenicity evaluation of MVA-CMDR, a multigenic, recombinant modified vaccinia Ankara HIV-1 vaccine candidate. PLoS ONE 5(11): e13983 10.1371/journal.pone.0013983 21085591 PMC2981570

[39] Karnasuta C, Robert M, Paris RM, Cox J H, Nitayaphan S, et al (2005) Antibody-dependent cell-mediated cytotoxic responses in participants in a phase I/II ALVAC-HIV/AIDSVAX B/E prime-boost HIV-1 vaccine trial in Thailand. Vaccine 23:2522–2529. 15752839 10.1016/j.vaccine.2004.10.028

[40] Sun Y, Asmal M, Lane S, Permar SR, Schmidt SD, et al (2011) Antibody-dependent cell-mediated cytotoxicity in simian immunodeficiency virus-infected rhesus monkeys. J Virol 85:6906–6912. 10.1128/JVI.00326-11 21593181 PMC3126600

