# Supplementary figures and images for: Potent Functional Antibody Responses Elicited by HIV-I DNA Priming and Boosting with Heterologous HIV-1 Recombinant MVA in Healthy Tanzanian Adults
Source: PLoS One. 2015 Apr 14;10(4):e0118486. doi: 10.1371/journal.pone.0118486 (PMC4396991; doi:10.1371/journal.pone.0118486)

Figure S1


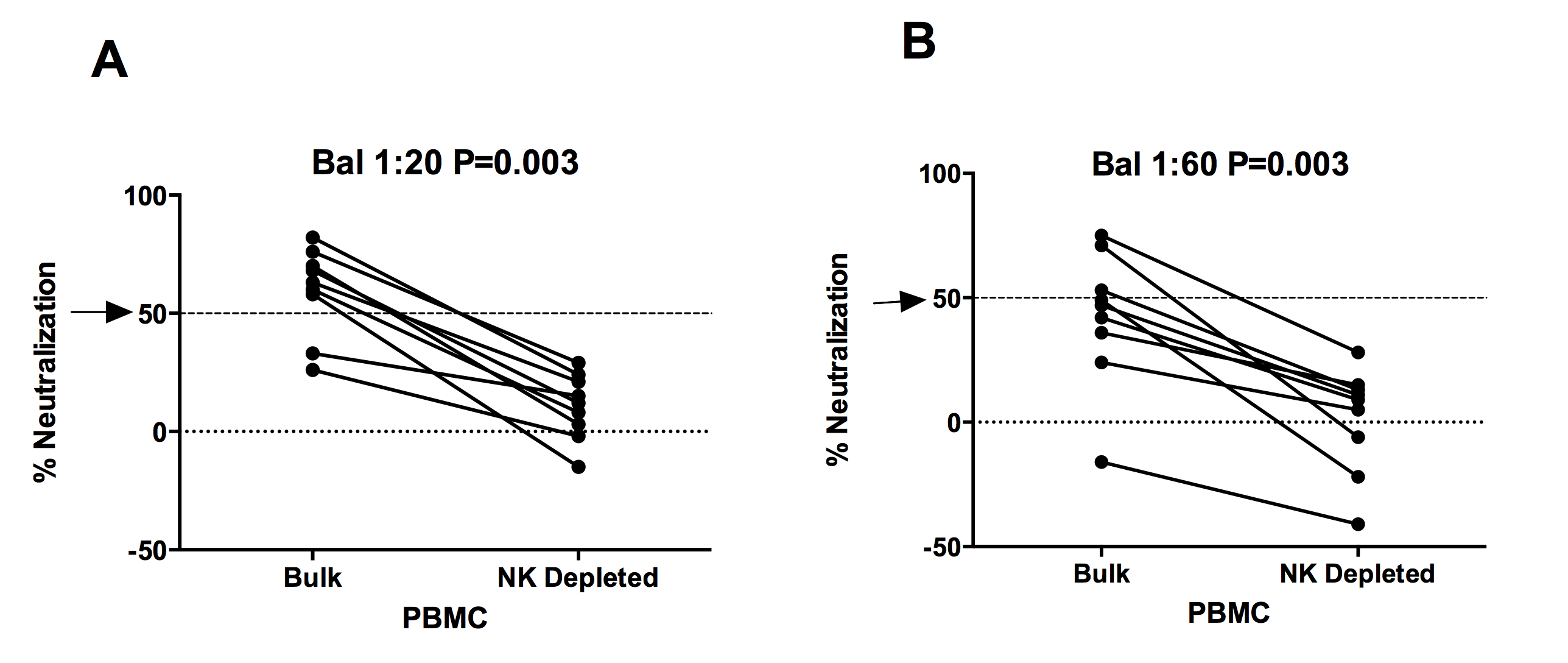


Figure S2


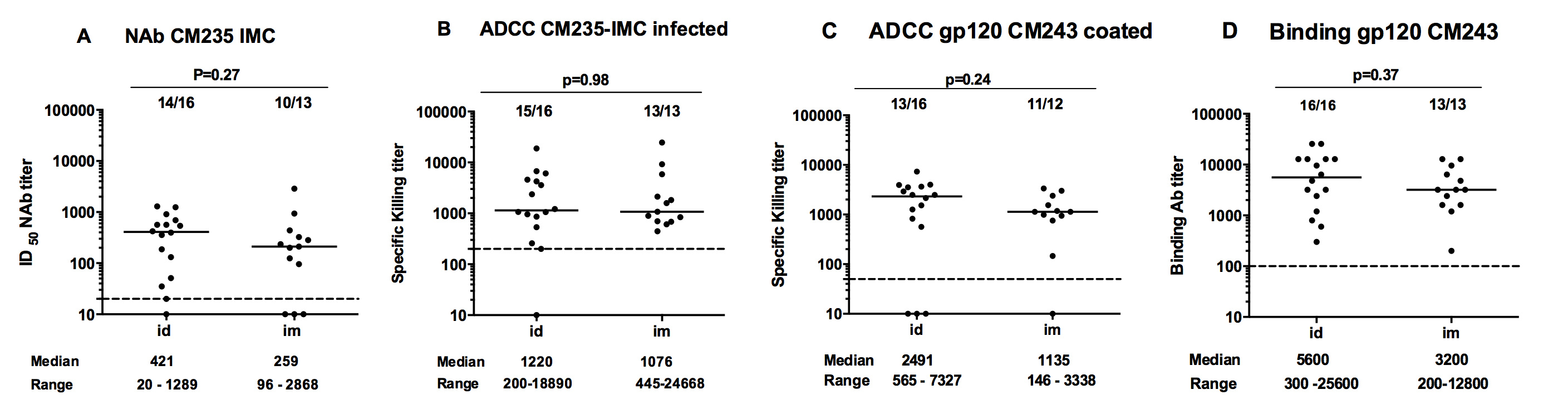


Figure S3


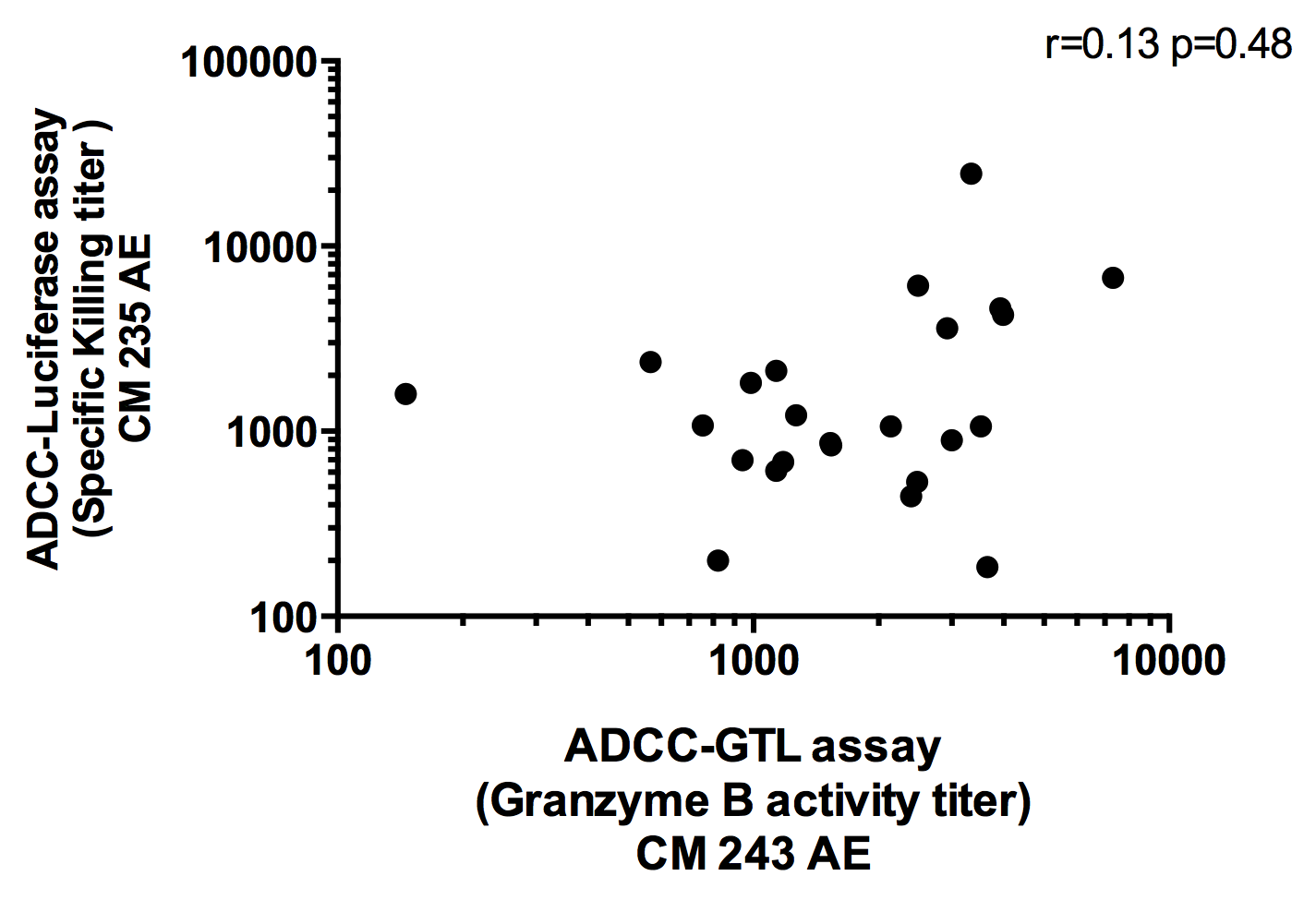

Supplement: S1 File — Fig S1. NK cell depletion from PBMC influenced the HIV-1 neutralizing antibody activity. NK cell depletion from PBMC influenced the HIV-1neutralizing antibody activity to subtype B HIV-1 BaL at two different serum dilutions 1:20 (A) and 1:60 (B), as measured using the IMC/PBMC neutralization assay. The black lines connect the neutralizing activity from same vaccinees’ sera before and after NK cell depletion. Fig S2. Antibody responses against CRF01_AE based on the route of HIV-MVA vaccination. Comparison of antibody responses against CRF01_AE based on the route of HIV-MVA vaccination (id versus im), as determined using the IMC/PBM neutralization assay (A), the ADCC-Luciferase assay (B), the ADCC-GTL assay (C) and ELISA binding titers (D). Sera with no activity or negative values in the assays were arbitrarily assigned a reciprocal titer of 10. Fig S3. Spearman rank correlation of ADCC responses. No correlation was seen between ADCC responses to CM235 CRF01_AE as measured by the ADCC-Luciferase assay and ADCC responses to CM243 CRF01_AE as measured by the ADCC-GTL assay (DOCX) [file pone.0118486.s001.docx]
