# Supplementary material for: Potent Functional Antibody Responses Elicited by HIV-I DNA Priming and Boosting with Heterologous HIV-1 Recombinant MVA in Healthy Tanzanian Adults
Source: PLoS One. 2015 Apr 14;10(4):e0118486. doi: 10.1371/journal.pone.0118486 (PMC4396991; doi:10.1371/journal.pone.0118486)
Supplement: S1 Protocol — A Phase I/II trial to assess the safety and immunogenicity of a plasmid DNA-MVA prime boost HIV-1 vaccine candidate among volunteers in Dar es Salaam, Tanzania (PDF) [file pone.0118486.s002.pdf]

RESEARCH PROTOCOL

TITLE: A Phase I/II trial to assess the Safety and Immunogenicity of a plasmid DNA-MVA prime boost HIV-1 vaccine candidate among volunteers in Dar es Salaam, Tanzania

Principal Investigator (PI):
Prof Fred Mhalu (MBChB, FRCPath, Dr MED)
Department of Microbiology/Immunology
Muhimbili University of Health and Allied Sciences (MUHAS)
P.O. Box 65001, Dar es Salaam, TANZANIA
fmhalu@muhas.ac.tz

Co-Principal Investigator:
Prof Kisali Pallangyo (MD, MMed)
Department of Internal Medicine
Muhimbili University of Health and Allied Sciences (MUHAS)
P.O. Box 65001, Dar es Salaam, TANZANIA
kpallangyo@muhas.ac.tz

Other Investigators:

A. Department of Internal Medicine, MUHAS&MNH
1. Dr Muhammad Bakari (MD, MMed, PhD), Project Director
2. Dr Ferdinand Mugusi (MD, MMed)
3. Dr Eric Aris (MD, MMed)
4. Dr Mohamed Janabi (MD, MSc, PhD)
5. Dr Patricia Munseri (MD, MMed, MPH)

B. National Institute for Medical Research (NIMR)
1. Dr Joel Francis (MD, MSc),Clinical Coordinator

C. Department of Microbiology/Immunology, MUHAS
1. Prof Eligius Lyamuya (MD, MMed, PhD), Laboratory Co-ordinator
2. Prof Willy Urassa (MD, MMed, PhD),
3. Dr Said Aboud (MD, Mphil, MMed)
4. Dr Judica Mbwana (MSc, PhD),Laboratory Manager

D. Department of Epidemiology and Biostatistics
1. Dr Candida Moshiro (PhD)

E. Ministry of Home Affairs, Police Force Medical Section
1. Dr Layon Mwanyika (MD, MMed)
2. Dr David Siyame (MD, MMed)

F. School of Nursing, MUHAS
1. Ms Edith Mrosso (BScN, Mphil)

G. Swedish Institute for Infectious Disease Control (SMI) & Karolinska Institute (KI)
1. Prof Gunnel Biberfeld (MD, PhD)
2. Prof Britta Wahren (MD, PhD)
3. Dr Charlotta Nilson (BSc, PhD)

H. Karolinska Institute & Sodersjukhuset, Stockholm, Sweden
1. Prof Eric Sandstrom (MD, PhD),HIVIS Project Coordinator
2. Dr Bo Hejdeman (MD, PhD)

I. University of Munich, Germany
1. Dr Michael Hoelscher (MD,PhD)

J. University of Cape Town, South Africa
1. Prof Carolyn Williams (PhD)

Protocol Identification Number: VERSION 04

DATED: 25th May, 2008

Signed:..............................
Professor Fred Mhalu
(Principal Investigator)

TRIAL SPONSORS:

1. Professor David Ngassappa
   Deputy Vice Chancellor (Academics, Research and Consultancy)
   Muhimbili University of Health and Allied Sciences (MUHAS)
   P.O. Box 65001
   Dar es Salaam
   TANZANIA

   Phone: +255 22 2150473
   E-mail: vcarc@muhas.ac.tz

   Signature:...

   Deputy Vice-Chancellor (Academic Research and Consultancy) Muhimbili University of Health and Allied Sciences P. O. Box 65001 DAR-ES-SALAAM

   3rd Sept- 08.

2. Ragnar Norrby, (MD, PhD)
   Swedish Institute for Infectious Disease Control
   Nobels väg
   18171 82
   Solna, SWEDEN

   Phone: +46 8 457 2310
   Fax: +46 8 30 36 68
   E-mail ragnar.norrby@~~smi.ki.se~~ smi.se

   Signature:...

SPONSOR MEDICAL EXPERTISE:

The following investigators and qualified physicians are the sponsor's medical experts that are responsible for trial related medical decisions:

1. Dr Muhammad Bakari (MD, MMed, PhD). Senior Lecturer, Department of Internal Medicine, MUHAS. P.O. Box 65001, Dar es Salaam, Tanzania

2. Dr Eric Aris (MD, MMed). Consultant Physician, Department of Internal Medicine, Muhimbili National Hospital (MNH), P.O. Box 65000, Dar es Salaam, Tanzania

3. Dr Mohamed Janabi (MD. MSc, PhD). Consultant Physician, Department of Internal Medicine, Muhimbili National Hospital (MNH), P.O. Box 65000, Dar es Salaam, Tanzania

4. Dr Patricia Munseri (MD, MMed). Lecturer, Department of Internal Medicine, MUHAS. P.O. Box 65001, Dar es Salaam, Tanzania

5. Prof Eric Sandstrom (MD, PhD). Institute of Clinical Research and Education, Sodersjukhuset, Karolinska Institutet (KI), Sweden.

TRIAL MONITORING:

Clinical Aspect:

1. Prof Eric Sandstrom (PhD)
Institute of Clinical Research and Education
Sodersjukhuset, Karolinska Institutet
Sjukhusbacke 10
S 118 83
Stockholm, SWEDEN

Phone: +46 8 616 2571
Fax:: +46 8 616 2509
E-mail: eric.sandstrom@sodersjukhuset.se

Laboratory Aspect:

2. Dr Charlotta Nilsson (PhD)
Swedish Institute for Infectious Disease Control (SMI) and Karolinska Institute (KI)
Nobelsvag 18
SE 17182
Solna, SWEDEN

Phone: +46 8 4572612
Fax: +46 8 337460
E-mail: charlotta.nilsson@smi.ki.se

PERSON AUTHORIZED TO SIGN THE PROTOCOL AND PROTOCOL AMENDMENTS FOR THE SPONSOR:

**Name**	**Title**
Prof. Fred Mhalu	Prinicpal Investigator

TRIAL SITE:

Muhimbili University of Health and Allied Sciences (MUHAS)
P.O.Box 65001
Dar es Salaam
TANZANIA

Phone: *+255 22 2153027*
Fax *+255 22 2153927*

SYNOPSIS

Objectives

1. To assess the safety of a plasmid DNA-MVA prime boost HIV-1 vaccine candidate among volunteers in Dar es Salaam, Tanzania.
2. To determine immunogenicity of a plasmid DNA-MVA prime boost HIV-1 vaccine candidate among volunteers in Dar es Salaam, Tanzania.
3. To build expertise and capability in evaluating HIV-1 vaccine candidates in Dar es Salaam, Tanzania

Methodology

Study Site

The clinical site will be MNH/MUHAS, Dar es Salaam, TANZANIA. Laboratory tests will be performed at the Department of Microbiology and Immunology, MUHAS, Dar es Salaam, and at the Swedish Institute for Infectious Disease Control, Stockholm, SWEDEN

Study Volunteers

Primarily, volunteers were recruited from the Police Force in Dar es Salaam with whom we have had extensive prior contact since 1994. The study has to date enrolled all the intended 60 volunteers, of whom 15 (25%) are females. They were all HIV negative by ELISA HIV antibody/antigen assay at the time of enrolment, and were all considered to be at low risk for HIV acquisition.

Study Design

Randomised, controlled, double blinded study. The laboratory staff will also be blinded to the immunization regimens.

**Arm**	**Volunteers**	**DNA immunization**	**MVA boost**
I	20	DNA 3.8mg intramuscularly by Bioject	MVA 10^8 pfu intramuscularly
II	20	DNA 1.0mg intradermally by Bioject	MVA 10^8 pfu intramuscularly
IIIa	10	Saline intramuscularly by Bioject	Saline i.m
IIIb	10	Saline id by Bioject	Saline i.m

The vaccine (immunogens)

Priming with a plasmid having HIV-1 DNA encoding the following genes:

1. env, from HIV-1 subtypes A, B, C
2. rev, from HIV-1 subtype B
3. gag, from HIV-1 subtypes A,B
4. Rtmut (mutated reverse transcriptase), from HIV-1 subtype B

Boosting will be effected by a Modified Vaccinia Ankara vaccine (MVA-CMDR). It is a a live recombinant poxvirus vector vaccine that has been genetically engineered to express the following HIV-1 genes:

1. gp160 (Subtype E, CM235), and
2. gag and pol (integrase-deleted and reverse transcriptase nonfunctional, Subtype A, CM240).

HIV-DNA/placebo will be given at weeks 0, 4 and 12 as injections. The 1st HIV-MVA/placebo boost injection will be given at 24 weeks (6 months) after the last (third) DNA/placebo priming injection. A 2nd MVA/placebo injection will be given at 80 weeks after enrolment.

Duration of Study
The enrolment took place between 20th Feb 2007 and 26th Feb 2008.Volunteers will be monitored up to 24 weeks after the last injection.

Dosage and product administration

DNA plasmids
The plasmids will be produced by Vecura, Huddinge, Stockholm, SWEDEN for the Swedish Institute for Infectious Disease Control, 171 82, Solna. SWEDEN, to be administered either i.m or i.d using a Biojector.

MVA
Will be provided by the Walter Reed Army Institute of Research, US Military HIV Research Program through the Karolinska Institute. It will be administered i.m in a dose of 10^8 infectious doses.

DOSES:

**Vaccine and delivery method**	**Route**	**Ampoule 1 (env), Left arm**	**Ampoule 2 (gag, Rtmut), Right arm**
DNA by Biojector	Intramuscularly	1.0 ml (tot 2.0 mg DNA)	0.9 ml (tot 1.8 mg DNA)
DNA by Biojector	Intradermally	3 injections of 100ul (Total 0.6mg DNA)	2 injections of 100ul (total 0.4mg DNA)

Placebo
Appropriate placebos (saline) for DNA and MVA will be supplied by the Swedish Institute for Infectious Disease Control and Walter Reed Army Institute, US Military HIV Research Program respectively.

Endpoints

<u>Primary</u>
The safety of immunization will be assessed by clinical features and standard biochemical and haematological laboratory tests. Adverse events will be evaluated by assessing local (pain, cutaneous reactions including induration), general (fever, chills, headache, nausea, vomiting, malaise, myalgia) and other unsolicited adverse events

within 7 days of vaccination as possibly or probably related to the immunization. Any grade 3 or 4 event will be taken as an indication that the vaccination arm is less tolerable. The magnitude and quality of humoral (neutralizing and binding antibodies) as well as cellular immune responses as elicited by the different schedules of immunization will be determined to assess immunogenicity.

Secondary
A qualitative and quantitative assessment of the presence of necessary infrastructure and human capacity to conduct HIV-related vaccine studies at MUHAS/MNH.

The HIVIS project, and the subsequent TaMoVaC-I project are undertaken as part of an implementation of the Tanzania's National HIV Vaccine Framework. Financial support has been received from the European Union (EU), Sida/SAREC, the Swedish Embassy in Tanzania through the Tanzania Government and lately from the EDCTP.

BACKGROUND AND LITERATURE REVIEW

HIVIS Project

The HIV Vaccine Immunogenicity Study (HIVIS) is a study of the United Republic of Tanzania that receives support from the European Union (EU) as a collaborative project involving the Karolinska Institute in Sweden, Walter Reed Army Institute of Research (WRAIR) USA, University of Munich, Germany, and the University of Cape Town, South Africa.

Global HIV-1 Situation

Globally, the major cause of AIDS is HIV-1, and by the end of 2004 it was estimated that more than 60 million people had been infected with HIV since the epidemic started, and that 39.4 million million people were living with HIV by the end of 2004 globally (1). In the year 2004 it was estimated that there were about 14,000 new infections occurring daily. The majority of these occur in developing countries of sub-Saharan Africa, Asia and South America. Sub-Saharan Africa remains by far the worst affected region, with 25.4 million people living with HIV at the end of 2004, compared to 24.4 million in 2002. Just under two thirds (64%) of all people living with HIV are in sub-Saharan Africa, as are more than three quarters (76%) of all women living with HIV. In 2004, an estimated 3.1 million people in the region became newly infected, while 2.3 million died of AIDS. Reports of alarmingly high national adult HIV prevalences are still a common place in Southern Africa. In Swaziland, for example, HIV prevalence among pregnant women was 39% in 2002, up from 34% in 2000 and only 4% in 1992. It is 18% in Malawi in the year 2003, 16% in Zambia and 25% in Zimbabwe. HIV infection in this part of the world is largely due to HIV-1 and the predominant mode of transmission is heterosexual contact.

HIV-1 situation in Tanzania

The first three cases of AIDS in Tanzania were reported in 1983 from the Kagera region in the northwest, and were serologically confirmed two years later. It was also shown then that different groups of populations had different prevalences of HIV. The National AIDS Control Programme estimated that by the end of 2002 over 2.4 million individuals had been infected by HIV, and that about 800,000 had already developed AIDS in Tanzania despite great efforts with health education campaigns (2). Though the actual prevalence of HIV-infection still varies from place to place, the national average for the adult population was estimated at 10% in the year 2003 by the National AIDS Control Programme (NACP). In the most populous city, Dar es Salaam, the seroprevalence among sex workers is between 40 and 60%. The prevalence among hotel workers was 10.4% (3) and among members of the police force and antenatal clinic attendees, presumably representing the general population, was found to range from 13-18% (4,5). Among blood donors at Muhimbili National Hospital (MNH) the prevalence of HIV was 8.7% (6). The age group that is particularly affected is 20-49 years, a group that is reproductively important, and also the economically most productive age group (2)

Need for an HIV vaccine

Although antiretroviral drugs have shown great promise in reducing the replication of HIV, the cost is substantial and side effects are a potentially limiting factor. Development of an effective and well-tolerated vaccine is likely to be the best way to stop further spread of the virus. Despite the on-going efforts, many uncertainties still remain about the most appropriate vaccines, the best effective timing of the immunization and correlates of protective immunity.

Current efforts to develop a HIV Vaccine

In many countries, development of candidate HIV vaccines are at an advanced stage, with some already evaluated in clinical trials. The first human trial of an HIV vaccine candidate was conducted in the United States in 1987. By mid 2004, more than 30 different HIV candidate vaccines had been tested in approximately 80 phase I/II trials.

The first phase I/II vaccine trial in a developing country was conducted in China in 1993 (8). Since then, more than 20 phase I/II trials have been completed in developing countries, the majority in Thailand (9 trials), but also in Brazil, Cuba, Haiti, Kenya, Peru, Trinidad, Uganda, Botswana and South Africa. The tested vaccine concepts included: envelope based candidate vaccines (gp120, V3 peptides and V3 protein); Canarypox and Modified Vaccinia Ankara (MVA) vectors, DNA constructs, and prime-boost combinations; Multiepitope DNA vaccine, Adenovirus vectored, VEE vectored, DNA/MVA prime-boost.

African experience

In some African countries, several efforts are being made to develop suitable HIV vaccines. By 2003, only four phase I/II HIV vaccine trials had been conducted in Africa. The first one, conducted in Uganda in 1999, was sponsored by the National Institute of Health (NIH) of the United States of America. In this ALVAC-HIV preventative HIV vaccine study, a canary pox vector containing HIV-1 clade B antigens was used. Immunogenicity was low but the vaccine elicited CD8+T-cell responses with detectable cross-activity against clade A and D antigens in a significant proportion of vaccine-recipients (9,10). Three other trials were sponsored by the International AIDS Vaccine Initiative (IAVI) and were conducted in Kenya in 2001 and 2002, and one on-going in Uganda. The vaccine concept being evaluated is based on a prime-boost combination using DNA and MVA candidate vaccines expressing a number of genes from clade A HIV-1 strain. It is reassuring that preliminary results from these trials have revealed that the concept is well tolerated (11). A more detailed account of vaccine trials in Uganda, Kenya and South Africa is provided below:

HIV vaccine trials in Uganda

These have largely benefited from the collaborative work between the US Military HIV Research Programme (USMHRP) and Makerere University. The UMNHRP plans to advance two major vaccine candidates to Phase III field efficacy testing. The first is an accelerated advancement of the National Institute of Allergy and Infectious Diseases (NIAID) Vaccine Research Centre (VRC) trivalent (clade A, B and C) DNA vaccine prime and Recombinant Adenovirus-5 (rAd5) vaccine boost to efficacy testing at sites in East Africa. The second approach, a DNA prime and MVA boost, will follow the first by two to three years allowing additional research and development efforts to improve the prime-boost vaccine strategies to elicit enhanced HIV-specific cellular and humoral immunity. In addition, the DNA/MVA approach may be required in parts of Africa where adenovirus type 5 antibody titers may limit the current DNA prime/rAd5 boost strategy.

It all began in May 1998, when the Honorable Crispus Kiyonga, Ugandan Minister of Health, formally invited the U.S. Army to conduct HIV and malaria research within Uganda. The U.S. Department of Defence formally acknowledged this new program, and collaborative research work began in August 1998. Makerere University and the Henry M. Jackson Foundation have established a non-profit organization for the express purpose of conducting collaborative HIV research, the Makerere University-Walter Reed Project (MUWRP).

MUWRP has administrative and laboratory facilities on the campus of Makerere University College of Medicine in Kampala, Uganda. In addition, a research clinic is located off campus

approximately 1 kilometer from the main lab/administrative complex. A cohort development project is under progress for a Phase III Vaccine Trial among the Adult population of Kayunga District in Uganda. This project receives funding from the U.S. President's Emergency Plan for AIDS Relief (PEPFAR) to enable us to provide ARVs for these participants, their family members and health care workers in the district.
In addition to cohort development activities, the USMHRP has conducted RV 156, a phase I trial of the VRC DNA candidate vaccine for HIV-1 in Kampala Uganda. This product expresses gag from subtype B HIV and envelop gp 140 from subtypes A, B and C. The study evaluated safety and immunogenicity among healthy, HIV negative, low risk, adult male and female volunteers between the ages of 18 and 40. 222 volunteers were screened to identify and enroll 27 men and 4 women. The vaccine regimen consisted of 3 x 4 mg injections of the DNA product at 0,1 and 2 months using the Biojecter system. All volunteers have received all vaccinations without significant local or systemic toxicity. Immunogenicity data is pending. The next study with VRC products will be an amendment to RV 156 to permit all currently available and willing volunteers to receive an injection of the VRC recombinant, replication incompetent Adenovirus type 5 vector which expresses cognate gene products. In addition, we plan to embark upon a phase I/II trial with 324 volunteers to evaluate the VRC DNA and Adenovirus type 5 vector alone and in combination in Uganda, Kenya and Tanzania.
Recruitment approaches included information seminars for community leaders, pre-existing groups and later the general public. Although the study was publicized through newspaper and radio advertisement, brochures and banners, and later seminars were conducted, the study faced several challenges during recruitment especially of women, and that misunderstandings were common. This necessitates the need more community sensitization and government support to conduct HIV vaccine trials. The other challenge to enrolment encountered was laboratory abnormalities, which advocates for determination of local reference ranges.

HIV vaccine trials in Kenya

By 2004, there have been four HIV vaccine trials in Nairobi, Kenya: a completed Phase I MVA study (subtype A); an on-going Phase I DNA study (subtype A); and an on-going Phase IIA DNA/MVA study. The studies are being conducted at the Kenya AIDS Vaccine Initiative (KAVI), a collaborative partnership among the University of Nairobi (UoN), the British Medical Research Council (MRC) and the Commercial Sex Workers (CSW) in Nairobi and Gambia who, despite repeated exposure to the virus, do not get infected. These women have been observed to have CTLs specific to HIV but no antibodies to HIV. The vaccines contain clade A HIV-1 gag (p17 and p24) and 25 CTL epitopes from HIV-1 (gag, pol and nef). The vaccine is in two forms, a naked DNA and MVA. Animal studies had shown best responses when given as DNA and MVA boost.
The Nairobi vaccine trials began in January 2001 and were the first HIV vaccine trials held in Kenya. There was no prior experience with the process of approval, but there was significant support from the Ministry of Health, Office of the President, Ministry of Education, and from the Cabinet. There were some initial misunderstandings, including Media interpretation of the clinical trial concept as experimenting with people, and the perception that Kenyans would be used as guinea pigs; issues pertaining to patents and intellectual property rights of the vaccine product, concern that the vaccine might result in the participants being HIV-infected; Questions whether the vaccine was for therapeutic purposes; and fears of the confidentiality of the volunteers and possible stigmatization of participating volunteers. To resolve these challenges, meetings were organized with the stakeholders (members of parliament, the media, religious

groups, and community leaders). There was also a tripartite agreement and signing of a Memorandum of Understanding among the parties involved: UoN, MRC, and IAVI.
The main recruitment strategies for the early vaccine trials were seminars in tertiary educational institutes and at the trial site, use of posters, radio, television, and print media. Word of mouth from volunteers to friends and relatives was also very effective. These strategies were time consuming, but produced good results. The majority of volunteers were men. Current strategies involve community-based seminars, with 13 peer leaders in target communities identified and trained monthly for 7 months. These peer leaders should be respected members of the society, easily available and reliable. Their training curriculum focuses on HIV vaccines, informed consent process, and confidentiality. There are also community activities aimed at recruiting volunteers, such as outreach clinics and HIV awareness walks. Couples Voluntary Counseling and Testing (CVCT) has been introduced in an attempt to recruit more women. The main reasons for volunteering are cited as 80% humanitarian, 15% to know health status, and 5% for other reasons.
Lessons learned from the vaccine trials included: usefulness of stakeholders involvement; positive response from the communities; motivated volunteers; and very good follow up rates of up to 95%. Word of mouth was the most effective way of recruiting participants with the community recruitment approach being the most suitable.
Currently, a multi-site study is on-going to determine the reference ranges for the local population, having noted that during enrolment, 40% of subjects were rendered ineligible due to abnormal biochemistry based on reference values from the developed countries.
Preliminary results from the on-going phase IIA dose and route study involving 115 volunteers recruited in Nairobi and London of a DNA prime MVA boost vaccines expressing clade A HIV-1 gag and 25 defined CTL epitopes found that the vaccines were generally safe and well tolerated, and that the immunogenicity was poor irrespective of MVA dosage or route.

HIV vaccine trials in South Africa

HIV vaccine trials in South Africa are being run under the South African AIDS Vaccine Initiative (SAAVI) whose long-term strategy is to have multiple, concurrent trials for internal SAAVI-designed products, products developed in collaboration with other groups, and products developed externally.
SAAVI has four to five trial sites with the capacity to run phase I/II trials, and is now launching a phase III preparedness program. A detailed community preparedness program emphasizes ethics and human rights, and recognizes the need to include adolescents in trials.
By 2004, SAAVI was involved in two phase I trials: VEE-based vaccine and MVA-based vaccine. The VEE-based vaccine trial is a model of collaboration, and is looking at four different doses of VEE replicons. All volunteers in the trial had been recruited for the two-dose trial, and the next set of doses was in regulatory review. The MVA-based vaccine trial is a collaboration between African, European and American Scietists.
There were about ten clinical trials in the planning stages with various partners. Two SAAVI products under development, a DNA-based vaccines and a MVA-based vaccine, will be the first African products to be tested.
In 2004 the Cape Town HIV vaccine Clinical Trials Consortium began a study to recruit, enroll and retain over 12 months of follow-up or until HIV infection occurs a cohort of 200 persons living in a typical South African peri-urban township, as a preparatory effort for a phase III HIV vaccine trial.

Phase III trials
The first phase III HIV vaccine trials, assessing the protective efficacy of two different versions of a gp120 candidate vaccine, were initiated in North America and Europe in 1998 and in Thailand in 1999. Preliminary results from the North American trial became available in February 2003, and they showed that the vaccine failed to confer protective efficacy in the majority of the population (12). A preliminary subset analysis of less than 10% of the enrolled volunteers, suggested vaccine efficacy among black volunteers, however the interpretation of those data are still being debated. The data also suggest that women produced higher levels of antibodies than men and that vaccinated volunteers preferentially excluded viruses resembling vaccine antigens (virus sieving) (12). Those results are controversial and additional data are needed before a definitive conclusion can be reached. Preliminary results from the Thai trial indicate that the vaccine failed to confer protection (12). A second phase III trial, with an Alvac vector and gp 160 started in Thailand in 2003, with definitive results expected in 2008.

Need for developing countries to participate in HIV vaccine trials
Immunogenicity of vaccines in the industrialized world populations does not predict the response in developing countries. Nutritional deficiencies, genetic differences, and microbial burden in individuals may alter immune constitution. Such differences have been documented with polio, rabies, and yellow fever vaccines. In addition, the distribution of MHC Class I and Class II HLA types is determined genetically and is highly divergent across populations of different racial origins. Both humoral and cellular responses are determined to a large extent by the particular MHC Class I and Class II types in vaccine recipients. Therefore, the humoral and cellular immune reponses to immunogens should be compared with those responses among individuals in the developed world receiving the same vaccines.

Need for African countries to participate in HIV vaccine development and evaluation
It is important to conduct HIV vaccine trials in developing countries including those in Africa because the genetic variability of HIV may necessitate testing of vaccine candidates in different areas of the world, where different subtypes are prevalent. Furthermore, it may also be necessary to evaluate how different infection routes, cofactors for HIV transmission and host genetic backgrounds influence vaccine-induced protection. Licensing of a successful vaccine by regulatory bodies may require prior trials in countries with similar epidemiological settings.

Further, the conduct of HIV vaccine trials will in themselves contribute to capacity building and infrastructure development in Africa. In addition, if African countries are not involved in HIV vaccine development, clinical trial findings and benefits from developed countries may not be generalizable and may take a long time to benefit Africa. Participation of Africa in HIV vaccine trials will also provide an opportunity for scientists to generate results in a shorter period using a small sample size due to the high incidence of HIV in the region. By participating in vaccine trials, African countries will have ownership of results and thus guarantees that vaccine found to be efficacious would be available for Africa. Furthermore, the results obtained will give immediate access to the immunologically effective vaccine candidates.

Need for an HIV vaccine for Africa
An AIDS vaccine is urgently required for Africa and special efforts to support the development of such a vaccine relevant to Africa in the shortest possible period are needed for the following reasons:

- The majority (70%) of new HIV infections in the world continue to occur in Africa.
- AIDS has serious and devastating social, economic, health and security consequences and is threatening our own existence in Africa.
- Treatment for HIV/AIDS including access to antiretroviral therapy is not yet readily available to most affected individuals in Africa and will therefore, be unlikely to suffice in HIV prevention and control.
- Private sector market driven HIV vaccine research and development has mainly been directed to HIV of subtype B, while in Africa many subtypes are prevalent.

HIV vaccine strategies under investigation

Early candidate HIV-1 vaccines employed non-replicating compounds such as recombinant HIV-1 proteins. Vaccination of humans or non-human primates with recombinant HIV-1 or Simian Immunodeficiency Virus (SIV) proteins has generated specific antibody responses, but not generally protective immunity.

Vaccine strategies attempting to induce both enhanced T cell and antibody responses have focused primarily on the prime boost strategy (13).

It has been suggested that HIV-1 specific T cell responses may facilitate protective immunity. Individuals who are exposed to HIV-1, but do not become persistently infected, develop HIV-1 specific cytotoxic T lymphocytes (CTL) and T-helper lymphocytes without the generation of systemic HIV-1 antibodies, although mucosal HIV-1 antibodies have also been detected (14, 15). The generation of CTL and T- helper responses correlate in time with the control of acute HIV-1 viraemia in humans and macaques. Such a correlation is not seen with antibodies and, therefore, the induction of HIV-1 specific CTL and T-helper responses is widely seen as critical to the success of an HIV-1 vaccine.

There is thus a broad scientific consensus that a successful vaccine to prevent HIV-1 transmission must be able to elicit HIV-specific CD8+ cytotoxic T-lymphocytes (CTL) and also antibodies capable of neutralising primary HIV isolates (Nab). These include live, attenuated vaccines; inactivated viruses with adjuvants; subunit vaccines with adjuvants; live-vector based vaccines; and DNA vaccines.

Subunit vaccines, such as highly purified recombinant monomeric HIV-1 envelope proteins elicit neither virus-specific CTL nor antibody responses that can neutralize primary patients' isolates of HIV-1, even when adjuvanted with potent immunostimulants. Recently the first HIV vaccine efficacy trials with gp120 have been completed. Antibody was induced, but there was no significant protection against primary infections.

At the present, combining DNA vaccines and live-vector based vaccines in prime-boost regimens appears to be the most promising vaccine strategy. However many issues remain to be addressed. One issue is the efficacy of the DNA immunization, which is the topic of this investigation. Another issue is that animal studies so far indicate that this approach might offer a limited protection from infection, while the protection from development of HIV disease might be its main feature. This means a change in paradigm in vaccine development and has profound implications on vaccine efficacy trials and prospects for further HIV transmission.

HIV exists in many subtypes with different prevalences in different parts of the world. It is as yet unclear if immunization with one subtype will induce immunity to a different subtype. This may vary between different viral proteins. Today it is prudent to include antigens from the subtypes that are prevalent in the area where the vaccine is planned to be used.
In the current trial plasmids containing genes for ***env*** of subtypes A and C are included since these are the major subtypes present in Tanzania (16-20). ***Env*** of subtype B is included since it is closely related to subtype D that also is circulating in Tanzania, and is also highly immunogenic. Subtype B is the dominating subtype in Europe and the US. ***Gag*** of subtypes A and B are included for the same reason. The vaccine is complemented with ***RT*** of subtype B, since the pol gene does not vary so much between the subtypes. ***Rev*** of subtype B is included to augment the expression of the envelope genes. The optimal antigens to be used in a vaccine have not been determined. At this point it is advisable to include as many as practically feasible, as long as there is no risk of reconstructing a viable virus. Env is desirable primarily in order to raise antibodies. Since it is highly variable it is prudent to try to match it as to the predominating subtypes. ***Gag*** and ***RT*** are known to give rise to potent CTL responses and are less likely to vary. Additional desirable immunogens would be early regulatory proteins such as the genes for ***nef*** and ***tat.***

DNA HIV Vaccines

Plasmid DNA has been shown to induce primarily cell-mediated memory immune responses, but these responses have generally been weak. They have been successfully boosted by immunogens included in various viral vectors, such as vaccinia, fowlpox or adenoviruses. However the doses of DNA that need to be given are substantial and it would be a great advantage if more efficient delivery modes/routes can be established.

The favored route of injection has been intramuscular injection by needle. Recently immunogenicity has been increased by using a needle-less device that disperses the plasmids to a wider area in the tissue. It has also been found that intradermal injections will target antigen-presenting cells that are present in the skin. Intradermal injections given by needle are difficult to standardize due to technical difficulties. Injection by needleless devices can potentially offer advantages such as better dispersion of vaccine in tissues, standardized inoculation and less risk of exposure to unsterilized needles. Disposable single use devices are now available. One such commercially available device is the Bioject®, which is to be used in this study. The risk for cross-contamination, when multiple use devices are used for vaccination is now addressed by use of exchangeable single use nozzles. This means that these devices will be adapted to mass use. Needleless devices can also be used for intradermal injections. The present study sets out to study needleless injections in the muscle and skin.

DNA Toxicology Reports in Animals

Preclinical studies have been conducted in animals and reported DNA to be safe:
<u>In study 1</u>; (2001-11-25) the p37 gag gene, the inactivated RT gene as well as placebo in increasing doses (1-100 µg) was administered intraperitoneally (i.p.) in mice. The mice showed neither growth retardation nor signs of toxicity throughout the experiment.

<u>Studies 2 and 3</u>; (2001-10-22, 2001-11-26, 2002-02-06); the pKCMVp3gag and pKCMVRTmut was delivered i.p. to mice. Seven weeks after administration, Preliminary anatomic diagnosis (PAD) showed no abnormal findings. The pKCMVnef was delivered Intradermally by gene gun

to mice. Two hours after i.d. administration, plasmid DNA was detectable in the skin and draining lymph nodes. At 24 hours, DNA was detected only in the skin, and after 48 hours no DNA could be detected in any organ. The following organs were studied; lung, liver, uterus with ovaries, lymph nodes, kidney, brain, spleen, gut, heart, skin ventral (gene gun area), and dorsal skin. PAD revealed inflammatory reaction at day 14 of the ventral skin. Otherwise, there were no abnormal findings.

Study 4 (2003-03-06); the HIV *env-gag-pol* genes have been studied for repeat dose toxicity in a GLP study of a different vector (MVA) in rabbits by collaborators at the Henry M Jackson foundation. Seventy-two adult rabbits (24 placebo) of both sexes received four i.m. or i.d. doses of the vaccine. There were no signs of toxicity in this study.

Study 5 (2003-05-06); fifty-four Balb/c mice and six placebo mice received plasmids encoding *env A* or *env B* or *env C* i.p. in doses of 5, 50 or 500 µg. Pathology of lung, liver, uterus, lymph node, kidney, brain, spleen, intestine, heart and muscle showed normal gross histopathology. Body weight development in immunized mice was not different from un-immunized controls during this study.

Single dose biodistribution
When a single dose biodistribution was performed in these studies, it was found that in study 1 (2001-10-22); five weeks after delivery of a human dose of the *ga*-encoding plasmid and the *Rt*mut encoding plasmid (100 µg i.p.) to mice, no plasmid DNA was detected in any of the investigated organs; lung, liver, uterus with ovaries, lymph nodes, kidney, brain, spleen, gut or heart.
In 2 studies the pKCMV nef was delivered intradermally by gene gun to mice. Two hours after i.d. administration, plasmid DNA was detectable in the skin and draining lymph nodes. At 24 hours, DNA was detected only in the skin, and after 48 hours no DNA could be detected in any organ. The following organs were studied; lung, liver, uterus with ovaries, lymph nodes, kidney, brain, spleen, gut, heart, skin ventral 1 (gene gun area), and dorsal skin.
Study 3 performed in collaboration with SBL vaccine AB, Solna, Sweden using standardized procedures. Balb/c mice were immunized i.m. with 50 µg plasmid DNA encoding the envelope gene from subtype A, B and C. Organs were collected at 2 hours, 72 hours and 3 weeks. DNA was detected in muscle and draining lymph nodes after 2 hours. After 72 hours or later, no DNA could be detected in the investigated tissues.
Study 4 was performed using stnadrdized procedures at the Swedish Institute for Infectious Disease Control and MTC, Karolinska Institute. It was done according to the anticipated HIVIS delivery program. C57 B1/6 mice were immunized with 200 mg of the HIVIS vaccine, either i.m. with GM-CSF or i.d. with the Biojector. The following organs were collected: lung; liver; testes; kidney; intestines; brain; heart; spleen; draining lymph nodes closest to the site of i.m. inoculation, right side; draining lymph nodes closest to the site of i.m. inoculation, left side; quadriceps muscle , right side; quadriceps muscle left side. At two hours, plasmids were found in injected organs (skin and muscle) and lymph node draining the injected organ, as well as in skin and muscle at the contralateral side. At 72 hours plamid was not discovered in any organ. Complete assays were performed for both *env* plasmids were found in any organ; controlled by both *env* and *gag* plasmid

Immunogenicity and challenge models of DNA vaccine pre-clinical trials in animals
In mice
Inbred strains of mice: C57BL/6 and Balb/C were used to determine immunogenicity since they will develop Th1 and Th2 focussed responses respectively. Challenge with HIV-1 has been performed in an HIV-1/MuLV pseudovirus system. It was shown that a combination of envelope genes A, B and C gave a better protection against HIV-1 challenge than either the B alone or A+C genes. Furthermore it has been shown that all the proposed DNA from gag, RT and env of subtypes A, B and C are highly immunogenic. In this model the following modes of delivery were most efficient: Biojector, intranasal and intramuscular. GM-CSF given as a protein immediately before the DNA injection was a very good adjuvant to increase both Th2 and Th1 responses. By gene gun delivery it was possible to lower the doses of DNA, however the immunogens mainly gave Th2 reactivities (21, 22, 23)

In macaques
Experiments in macaques have shown the safety, immunogenicity and protective efficacy of a prime-boost vaccine regimen based on plasmid DNA expressing various HIV-1 (env, gag, RT, rev, tat, nef) and SIV (SIVmac gag/pol) immunogens followed by MVA expressing the same immunogens (24, 25). In the first experiments we found that intramuscular immunization followed by mucosal immunization with a jet injector induced stronger cellular immune responses and more effective control of SHIV challenge virus (given intravenously) than intramuscular immunization only. All vaccinated monkeys had reduced viral load compared to controls and one of 8 vaccinated monkeys was completely protected against infection. In the second experiment all monkeys (n=6) were first immunized with DNA vaccine intramuscularly and then mucosally with a jet injector followed by two booster immunizations with MVA vaccine. All monkeys showed good cellular immune responses as measured by the ELISPOT IFN-gamma assay and the lymphocyte proliferation assay. Two weeks after intra-rectal SHIV challenge the median plasma viral load was two logs lower in the vaccinated monkeys than in the naive controls.

The Modified Vaccinia Ankara (MVA)
The Modified Vaccinia Ankara (MVA) was derived from vaccinia virus strain Ankara (WT) by over 570 serial passages in chick embryo fibroblast cells (CEFC) (26, 27, 28). The resulting MVA strain lost the capacity to productively infect mammalian cells and suffered seven major deletions of DNA, including at least two host-range genes (K1L and C7L) (29, 30)

Origin of MVA (MVA-CMDR)
MVA is originated from the Dermovaccinia strain CVA. The early history of both CVA and MVA is given in Mayr et al. Infection 3:6-14 (1975). In summary from that article, CVA was retained for many years (Ankara Vaccination Station) via donkey-calf-donkey passages. In 1953, Mayr et. al.- purified it and passaged it twice through cattle. In 1954-55, the MVA strain was transferred to the State Institution, Bayerische Landesimpfantalt, and used by the Federal Republic of Germany as a smallpox vaccine. No adverse effects were documented in the 120,000 vaccinated individuals.

In 1958, attenuation experiments with CVA were begun in the laboratory of Dr. Anton Mayr (Institute for Microbiology and Infectious Diseases of Animals, University of Munich, Munich, Germany) by serial passage of terminal dilutions in chicken embryo fibroblasts (CEF). After the

360th passage, the virus was cloned by three successive plaque purifications and maintained in CEF through approximately 570 passages in Dr. Mayr's laboratory. At passage 516, and after its clinical examination in humans, the virus was named "Modified Vaccinia Virus Ankara" or "MVA" Stock to distinguish it from other attenuated vaccinia strains. Dr-Gerd Sutter, working in the laboratory of Dr. Anton Mayr, amplified MVA P572 (2/22/74) to P574 in CEF at which time he plaque purified it by limiting dilution three times in CEF. The first large stock preparation of this further plaque purified virus is P580 8/15/88 (this virus is also referred to as the "F6 clone"). Lyophilized MVA P580 8/15188 was brought by Dr. Gerd Sutter in December 1990 to the laboratory of Dr. Bernard Moss, Laboratory of Viral Diseases, National Institutes of Allergy and Infectious Diseases, National Institutes of Health, Bethesda, and used in the creation of MVA-CMDR.

Previous Studies with MVA Constructs

Animal Studies

Several animal studies have been conducted with various recombinant MVA constructs expressing non-retroviral (influenza, parainfluenza 3, respiratory syncytial virus, malaria, Japanese encephalitis virus, equine herpes virus type 1, tumor associated) and retroviral (SIV, HIV) antigens (31-39). The murine studies collectively show that both protective antibody and CTL activity are induced after rMVA vaccination by various routes: intranasally, subcutaneously, intramuscularly, intravenously; 10^3 to 10^8 infectious units (21) and protection is conferred to both infectious and tumor challenges. While all routes were immunogenic, not all routes were equally protective in all animal model systems. Nuances of immunogenicity variability due to route of infection differences will likely have to be sorted out in humans, as animal model routes do not always convey the same information to humans. Nonetheless, pre-clinical studies provide abundant evidence for MVA as a useful candidate vaccine vector.

Primate studies

The dynamics of plasma viremia have been explored in SIV-infected rhesus macaques (SIVsm E660) that had received prior immunization with either control MVA or rMVA expressing SIV gag-pol, env (im, 5 x 10^8 CCID50), or recombinant vaccinia vaccine (rVV) expressing SIV gag-pol, env (id, 10^8 pfu/mL) at 0, 12, 20 and 28 weeks. Three of four macaques immunized with rMVA showed low levels of primary plasma viremia with maintenance of normal lymphocyte subsets and intact lymphoid architecture as compared with other groups, which showed high levels of plasma viremia and CD4 T cell decline. These data link the dynamics and extent of virus replication to disease course and suggest that sustained suppression of virus promotes long-term, asymptomatic survival of SIV-infected macaques (40). The utility of modified vaccinia virus Ankara (MVA) as a vector for eliciting virus-specific cytotoxic T lymphocytes (CTL) was explored in the simian immunodeficiency virus (SIV)/rhesus monkey model. After two intramuscular immunizations (0 and 13 weeks, 10^8 pfu) with recombinant MVA-SIV-SM expressing gag and pol, three of the four Mamu-A*01 monkeys developed a gag epitope-specific CTL response readily detected in peripheral blood lymphocytes after the first immunization and in all macaques after boosting. The elicited CTL response could be boosted with repeated MVA-SIV immunizations. This result suggests that immunity to the vector was insufficient to limit viral protein expression on repeated inoculations. Moreover, those immunizations also elicited a population of CD8+ T lymphocytes in the peripheral blood that bound a specific major histocompatibility complex class I / peptide tetramer. These gag epitope specific CD8+ T lymphocytes also were demonstrated by using both functional and tetramer-binding assays in lymph nodes of the immunized monkeys. These observations suggest that MVA may prove a

useful vector for an HIV-1 vaccine and that tetramer staining may be a useful technology for monitoring CTL generation in vaccine trials in nonhuman primates and in humans (41). More recently, by using a multicytotoxic T-lymphocyte (CTL) epitope gene and a DNA prime-MVA boost vaccination regimen, high levels of CTLs specific for a single simian immunodeficiency virus (SIV) gag-derived epitope were elicited in rhesus macaques. These vaccine-induced CTLs were capable of killing SIV-infected cells in vitro. Flow cytometric analysis using soluble tetrameric major histocompatibility complex-peptide complexes showed that the vaccinated animals had 1 to 5% circulating CD8(+) lymphocytes specific for the vaccine epitope, frequencies comparable to those in SIV-infected monkeys. Upon intrarectal challenge with pathogenic SIVmac251, no evidence for protection was observed in at least two of the three vaccinated animals. This study demonstrates that the DNA prime-MVA boost regimen is an effective protocol for induction of CTLs in macaques. It also shows that powerful tools for studying the role of CTLs in the control of SIV and HIV infections are now available (42).

The immunogenicity and protective efficacy of a modified vaccinia virus Ankara (MVA) recombinant expressing the simian immunodeficiency virus (SIV) Gag-Pol proteins (MVA-gag-pol) was explored in rhesus monkeys expressing the major histocompatibility complex (MHC) class I allele, Macaque mulatta (Mamu) A*01. Macaques received four sequential intramuscular immunizations with the MVA-gag-pol recombinant virus or nonrecombinant MVA as a control. Gagspecific cytotoxic T-lymphocyte (CTL) responses were detected in all MVA-gagpol-immunized macaques by both functional assays and flow cytometric analyses of CD8+ T cells that bound a specific MHC complex class I-peptide tetramer, with levels peaking after the second immunization. Following challenge with uncloned SIVsmE660, all macaques became infected; however, viral load set points were lower in MVA-gag-pol-immunized macaques than in the MVA-immunized control macaques. MVA-gag-pol-immunized macaques exhibited a rapid and substantial anamnestic CTL response specific for the p11C, C-M Gag epitope. The level at which CTL stabilized after resolution of primary viremia correlated inversely with plasma viral load set point ($p = 0.03$). Most importantly, the magnitude of reduction in viremia in the vaccinees was predicted by the magnitude of the vaccine-elicited CTL response prior to SIV challenge (43). Prior studies demonstrated that immunization of macaques with simian immunodeficiency virus (SIV) Gag-Pol and Env recombinants of the attenuated poxvirus modified vaccinia virus Ankara (MVA) provided protection from high levels of viremia and AIDS following challenge with a pathogenic strain of SIV. This MVA-SIV recombinant expressed relatively low levels of the Gag-Pol portion of the vaccine. To optimize protection, second-generation recombinant MVAs that expressed high levels of either gag-Pol (MVA-gag-pol) or env (MVAenv), alone or in combination (MVA-gag-pol-env), were generated. A cohort of 24 macaques was immunized with recombinant or non-recombinant MVA (four groups of six animals) and was challenged with 50 times the dose at which 50% of macaques are infected with uncloned pathogenic SIVsmE660. Although all animals became infected post-challenge, plasma viremia was significantly reduced in animals that received the MVA-SIV recombinant vaccines as compared with animals that received non-recombinant MVA ($p = 0.0011$ by repeated-measures analysis of variance). The differences in the degree of virus suppression achieved by the three MVA-SIV vaccines were not significant. Most importantly, the reduction in levels of viremia resulted in a significant increase in median ($p < 0.05$ by Student's t test) and cumulative ($p = 0.01$ by log rank test) survival. These results suggest that recombinant MVA has considerable potential as a vaccine vector for human AIDS (44).

Neutralizing antibodies were assessed before and after intravenous challenge with pathogenic SIVsmE660 in rhesus macaques that had been immunized with recombinant modified vaccinia virus Ankara expressing one or more simian immunodeficiency virus gene products (MVA-SIV). Animals receive either MVA-gag-pol, MVA-env, MVA-gag-pol-env, or non-recombinant MVA. Although no animals were completely protected from infection with SIV, animals immunized with recombinant MVA-SIV vaccines had lower virus loads and prolonged survival relative to control animals that received nonrecombinant MVA. Titers of neutralizing antibodies measured with the vaccine strain SIVsmH-4 were low in the MVA-env and MVA-gag-pol-env groups of animals and were undetectable in the MVA-gag-pol and non-recombinant MVA groups of animals on the day of challenge (four weeks after final immunization). Titers of SIVsmH-4-neutralizing antibodies remained unchanged one week later but increased approximately 100-fold two weeks post-challenge in the MVA-env and MVA-gag-pol-env groups while the titers remained low or undetectable in the MVA-gag-pol and nonrecombinant MVA groups. This anamnestic neutralizing antibody response was also detected with T-cell-line-adapted stocks of SIVmac251 and SIV/DeltaB670 but not with SIVmac239, as this latter virus resisted neutralization. Most animals in each group had high titers of SIVsmH-4-neutralizing antibodies eight weeks post-challenge. Titers of neutralizing antibodies were low or undetectable until about 12 weeks of infection in all groups of animals and showed little or no evidence of an anamnestic response when measured with SIVsmE660. The results indicate that recombinant MVA is a promising vector to use to prime for an anamnestic neutralizing antibody response following infection with primate lentiviruses that cause AIDS. However, the Env component of the present vaccine needs improvement in order to target a broad spectrum of viral variants, including those that resemble primary isolates (45).

Preclinical studies with the current MVA-HIV env/gag/pol E/A have shown this vector to be immunogenic in mice, eliciting envelope and p24 binding antibodies, proliferative responses, and CTL activity (Earl P, VanCott T et al. unpublished – see Investigator's Brochure). Other rMVA-SIV/SHIV constructs have shown both immunogenicity (cellular and humoral) and boosting with lower viral set points after challenge in pre-clinical testing (see Investigator's Brochure).

MVA Administered in Immunosuppressed Animals

Neither non-irradiated nor neutron-irradiated (300 rad) rabbits developed any signs of disease after an intravenous injection of MVA (5 x 10^8 pfu). In no case MVA could be recovered from internal organs. The animals showed a delayed but marked antibody response and were protected against a subsequent challenge with the vaccinia virus strain Elstree (46). MVA was found to be non-virulent for both adult and infant mice after the intracerebral inoculation of high doses. The MVA maintained these properties in immunosuppressed mice. All routes of immunization were well tolerated in animals including the intradermal route (0.2 mL at 10^6 cell culture infective dose; 50% of cells infected (CCID50)/mL) in rabbits and monkeys (47).

Severely immunodeficient mice (SCID) remained healthy for 133 days of observation when inoculated with MVA at 1000 times the lethal dose of vaccinia virus derived from the licensed Dryvax vaccine seed whereas all mice succumbed to the 3-log lower dose of the Wyeth vaccine seed strain within 30 days (48). Mice with B cell deficiencies or CD8 T cell deficiencies could be immunized with rMVA and were protected upon vaccinia challenge. However, mice with CD4 T cell or MHC Class II deficiencies were not protected after rMVA immunization.

MVA safety has also been studied in immune-suppressed macaques (total body irradiation-TBI, anti thymocyte globulin-ATG, or measles infection). Macaques were inoculated with high doses of MVA (109 pfu) via various routes: intradermal (ID), intranasal (IN), and intramuscular (IM). The vaccinations were well tolerated and no clinical, hematological or pathological abnormalities were observed. No MVA was cultivated from their tissues (using CEF culture system) when tested 13 days after inoculation (49)

Human Experience with MVA

As mentioned above, a large clinical experience was acquired when non-recombinant MVA was used for primary vaccination against smallpox by intradermal, subcutaneous, and intramuscular routes of over 120,000 humans against smallpox in southern Germany and Turkey. The original idea of using an attenuated vector such as MVA was to sequentially immunize first with MVA and boost with the classical vaccinia vaccine (VV) to offer protection against smallpox with fewer complications. During these extensive field studies, including high-risk patients, no major side effects were associated with the use of the MVA vaccine (50-52). Four schedules were tested in vaccinia naïve individuals who received a MVA dose at 10^6 CCID50 /mL:

MVA ID followed by VV scarification: 120,000 subjects were immunized with MVA (0.2 mL, ID) and received the second shot (VV) 14 days later (range five days to six months). The reactions to the first MVA immunization were a red papule; very few subjects (unspecified in report) had fever >38°C and most of them experienced mild systemic symptoms. The reactions to the second VV immunization consisted of accelerated mild pustular reaction and no fever. Primary reactions typically observed following VV administration were not observed in this study when VV immunization was preceded by an MVA priming.

MVA SC followed by VV scarification: Performed in toddlers (0.2 mL, SC). Palpable nodules four to five days post MVA. Same local reactions post VV as described above.

MVA IM followed by VV scarification: The lower MVA dose (0.2 mL, IM) was probably too low since all subjects developed primary responses to subsequent VV immunization. However a higher dose (0.5 mL) was well tolerated with no local or systemic reactions (N=193) and prevented the primary reaction to a second shot with VV as seen in above 2 parts.

MVA IM followed by MVA IM: Two 0.5 mL MVA IM injections (N = 10 subjects) were given 14 days apart (N = 10). Two of 10 subjects developed a small local infiltrate. No systemic reactions were reported. Four weeks after the post-second immunization, all raised their hemagglutination antibodies.

More recently, two British groups continue to test in humans two different rMVA constructs: one expressing malaria genes and the other expressing HIV-1 genes. The malaria-MVA study was recently published showing remarkable safety and tolerability of intradermal vaccination (53) and the induction of immune responses in a prime-boost strategy (54).

The HIV MVA vaccine studies are ongoing in both the UK and Uganda and Kenya (IAVI 003,005,008, and 009). These latter studies are part of a DNA prime MVA boost strategy. Recent work suggests that the rMVA–HIV clade A was well tolerated when delivered intradermally and immunogenic, stimulating HIV-specific T-cell responses in the majority of volunteers (55)

Another group based in Germany is giving a recombinant MVA-HIV (Bavaria Nordic) to HIV infected patients in an effort to enhance their immune responses (i.e. therapeutic vaccination). The patients are tolerating the injections well and enhanced immune responses are developing (personal communication Dr. Thomas Harrar, Erlangen, Germany). This study in seropositives will yield very important results regarding MVA-HIV safety because the participants are immunosuppressed, yet appear to tolerate the attenuated vaccine quite well.

Rationale for a Clinical Trial with MVA *env/ gag/pol* Subtype E/A

HIV-1 exists as multiple genetic subtypes (currently designated A-I, O) (56). In the United States, the vast majority of HIV-1 strains are Subtype B, whereas in Tanzania the circulating subtypes are A, C, D and their recombinants (16-20). The circulating Subtype E HIV-1, prevalent in South East Asia is characterized as a recombinant virus; the env gene derived from Subtype E and the gag/pol genes derived from Subtype A (56-58). Data from antibody cross-reactivity studies demonstrate that binding and neutralizing antibodies from Subtype B and E infected subjects react preferentially with viruses from the same subtype (59, 60). Conversely, several studies in HIV-infected subjects and also in vaccinees have shown some cross-subtype CTL activities, limited in breadth by the HLA polymorphism of individuals (61, 62). These studies along with the genetic and antigenic variability of HIV-1, the lack of specific knowledge of immune correlates of protection, and the lack of a predictive HIV-1 animal model illustrate the technical limitations of the formal approach to the development of HIV-1 vaccines.

A historically valid approach would be to empirically identify and test products that elicit strong humoral and cellular immune responses to viral strains prevalent within the region where the candidate vaccine will be evaluated. Such an approach is currently being tested in Thailand, i.e. producing and evaluating a recombinant MVA that incorporates genes/proteins isolated from individuals infected with HIV-1 Subtype E/A from Thailand (63-65). Although, this MVA-HIV construct does not match the HIV-1 Subtype B circulating in North America, it will be tested initially in the US among healthy HIV-uninfected volunteers for safety and immunogenicity. This is a conservative approach to perform early safety studies in the US before moving products into the international arena.

Human Experience to Date

Several MVA vaccines (e.g. MVA-malaria, MVA-HIV) are currently in Phase I international trials. Such as a HVTN 055 [A Phase I Trial to Evaluate the Safety and Immunogenicity of rMVA-HIV (rMVA-HIV env/gag+rMVA-HIV tat/rev/nef-RT) and rFPV-HIV (rFPV-HIV env/gag+rFPV-HIV tat/rev/nef-RT) Vaccines, Alone or in Combination, in Healthy, Vaccinia-Naïve HIV-1 Negative Participants] which is assessing the safety and immunogenicity of the simultaneous administration of two modified vaccinia Ankara (MVA)-vectored HIV vaccines as priming doses (one containing *env/gag* and the other containing *tat/rev/nef-RT*), followed by boosting doses of two fowlpox-vectored HIV vaccines containing identical inserts.

The product to be used in this study, MVA-VMDR (HIV-1 CM235 ENV/ CM240 GAG/POL) will have undergone a phase I trial (RV 158) in the USA. This trial is designed as a double-blind randomized dose escalating, placebo controlled study with the aim of determining the safety, tolerability and immunogenicity of the product as administered by the intramuscular or intradermal routes. The study will enroll 48 HIV-uninfected volunteers aged 18 to 49 years.

Prime – boost strategy
The rationale for the combined prime-boost regimen stems from pre-clinical data demonstrating that priming with DNA vaccines and boosting with pox virus vaccines induces stronger cellular immunity than either DNA or MVA alone. The DNA prime is thought to focus on immune response on the desired antigens, whereas the recombinant pox virus booster immunization is thought to boost this response, both by expressing higher levels of recombinant antigen and by the immunostimulatory activity of a pox virus infection. Excler and Plotkin (66) have recently reviewed prime-boost approaches to HIV preventive vaccination. They described animal data, as well as results from initial phase I clinical trials that assessed prime-boost regimens in HIV-1/2 uninfected volunteers using poxvirus-based HIV recombinants to prime and recombinant envelope glycoproteins to boost HIV-specific immune responses. The prime-boost approach induced both HIV-specific humoral and cell-mediated responses and conferred partial protection in animal models.

A Phase I HIV-1 vaccine trial in Sweden
A randomized, open label, phase I trial to assess the safety of different modes of administering the same DNA vaccine candidate (a plasmid DNA with inserted HIV genes env, rev, gag and RT) has recently been completed in Stockholm, Sweden. The combination of HIV-DNA and HIV-MVA proved safe and highly immunogenic. Thirty four out of 37 vaccinees (92%) having valid assays became positive in the IFN gamma ELISPOT assay, thirty-one vaccines reacted to Gag peptide pools (p17 and p24) and 24 to Env peptide pools (gp120 and gp41). Includng other assaysidentifying HIV-specific immunity, all but one vaccines, 37/38 (97%), developed HIV-specific immune responses. All DNA injections were delivered by the Bioject needleless device as is happening with the current HIVIS03 trial in Dar es Salaam, Tanzania. Preliminary analysis indicate that ¼ of the DNA given id primed equivalently to a 3.8mg dose given im, and that boosting with HIV-MVA can be achieved in volunteers despite remote small pox immunization. It was also shown that 10^8 pfu HIV-MVA im gave a better boosting of the HIV-DNA priming than did 10^7 pfu given id. Furthermore, volunteers below the age of 40 years exhibited higher levels of immune responses as compared to the other volunteers (>40 years) whose immune responses were markedly dampened (67).

The on-going Phase I/II trial (HIVIS03) in Tanzania
The HIVIS03 trial is now fully enrolled with the required 60 volunteers, all of whom came from the Police Force. 15 (25%) of them are females. Attendance at the scheduled follow up visits has so far been excellent. As of 19th Myay 2008 a total of 57 volunteers had already received the 3 DNA/Placebo vaccinations, and the immunogenicity data has shown very good results, with 19 out of 46 (41.3%) exhibiting responses by interferon gamma ELISPOT. A total of 26 volunteers have received their first MVA/placebo vaccination, and these have all not been associated with any untoward side effect.
Generally speaking, the vaccines have been very safe, and todate we have had 3 SAE's which are all unrelated to vaccination.

It is unfortunate that it was not possible to obtain MVA for the second boost in time to continue with MVA boosting as per earlier protocol. This was due to production difficulties at the WRAIR. These problems have now been solved, as a dedicated Contractor has been hired to carry on with the assignment of MVA production. The person in charge of the original production of the vaccine for the 1st boost, Dr Thomas vanCott, now works for that company. Hence the MVA vaccine is now expected to be available in Tanzania during October- November 2008. Indeed this amendment takes a further minor delay into account.
It is reassuring to note that so far the candidate vaccines being used in HIVIS 03 have been very safe as expected, and that the delay in providing a second MVA boost is not expected to negatively influence the safety or Immunogenicity results.
Furthermore, additional funds have been secured to cover the extension of study activities from the European and Developing Countries Clinical Trials Partnership (EDCTP) as one of the work packages under the Tanzania and Mozambique Vaccine (TaMoVac) programme for the period 2008 to 2011.
Among the volunteers, the issues regarding not fathering a child or becoming pregnant for 4 months after final immunization will be specifically addressed when the amendment is presented to the volunteers. Since all volunteers will be separately informed of the amendment and asked to sign a new informed consent explicitly stating their right to withdraw at any time, there will be an opportunity for a prolonged counseling that will address this issue, as well as the issues of HIV prevention. It is our belief that the Scientific information to be obtained out of this work is of great potential value, inspite of the minor discomfort which the study may cause on the volunteers.

Existing capacity for HIV-1 vaccine trial in Dar es Salaam
The HIV Vaccine Immunogenicity Study (HIVIS) is the daughter study of the TANSWED HIV programme. The TANSWED HIV programme in Tanzania is made up of 30 medical doctors at six departments at MUHAS and MNH, 6 investigators from the Police Force and 4 non-medical scientists from MUHAS/MNH/University of Dar es Salaam. It is a collaborative research programme between MUHAS and the Swedish Institute for Infectious Disease Control (SMI) and Karolinska Institute and the Umea University that has existed since 1986.
The overall aim of the programme is to generate information about the epidemiology, clinical manifestations, virology, serology, immunology, immunopathology, and socio-behavioural factors related to HIV infection. Furthermore to introduce and evaluate interventions for HIV prevention and control as well as strengthening the research capacity of the departments involved in the programme in Tanzania.

Through TANSWED HIV program, a strengthening of human capacity and laboratory infrastructure has taken place at MUHAS/MNH. The clinical trial group at the department of Internal medicine, MUHAS/MNH has one full professor, one Associate Professor, one Senior Lecturer, one Lecturer, 8 Medical specialists, 1 nurse and 3 nurse /counselors.
Clinical investigators involved with this project have been instrumental in the establishment in December 1999, and for the eventual growth of an HIV clinic at the Muhimbili National Hospital (MNH), as well as the planning of Tanzania's HIV/AIDS Care, Treatment and Support Plan. The HIV clinic began by providing counseling, treatment of opportunistic infections and provision of cotrimoxazole prophylaxis, and beginning July, 2004 the clinic took the lead in a pilot project of providing ARV's an experience which was instrumental in the initiation of Tanzania Government's National ARV scale up programme which began in October 2004.

Initially running once a week, the clinic currently runs daily with about 70-80 patients being seen on each of the 5 days of the week.
As of Dec 2004 a total of 1600 patients had been enrolled into the clinic, of whom 954 had been started on ARV's and continued to be followed up at the clinic. This clinic provides an important basis for ethical conduct of HIV vaccine trials in terms of providing care to the potential study participants who may be found with HIV during screening or who may seroconvert to HIV during vaccine trials.

The staff at the department of Microbiology and Immunology includes one Professor, one Associate Professor, one Senior Lecturer, one Lecturer, one Scientist and 5 Laboratory technologists.

The TANSWED HIV programme has funded the refurbishment of laboratory rooms and provided laboratory equipment for HIV serology and cellular immunology work as well as HIV DNA and RNA PCR testing. The equipment includes 6 laminar flow hoods, ELISA readers, a haemocytometer, a FACScan flow cytometer, a FACScalibur flow cytometer, a FACS count machine, a cell harvester and Betaplate reader for measurement of thymidine incorporation in lymphocyte proliferation assays, 2 liquid nitrogen containers, several –20 C and –70 C freezers, a thermocycler for PCR, refrigerated centrifuges, and a back-up electricity generator.

HIV laboratory assays performed at the laboratory include HIV serological assays (ELISA, rapid simple assays, Western blot analysis), lymphocyte subset determinations by flow cytometry and FACS count, haematological investigations by haemocytometry, lymphocyte proliferation assay by thymidine incorporation, ELISPOT-IFN gamma assay, HIV DNA and RNA determinations by PCR. A lymphocyte proliferation assay using whole blood and flow cytometry and the intracellular cytokine staining method for IFN gamma determination are being established.

As a continuation of HIVIS, a European funded project, abbreviated as TaMoVaC (Tanzania and Mozambique Vaccine) Project will be in operation beginning March 2008. It is a consortium involving institutions in the North (Karolinska Institutte and Swedish Institutte for Infectious Disease Control in Sweden; University of Munich in Germany; Imperial College in UK), and from the South (MUHAS, National Institute for Medical Research and Mbeya Medical Research Programme in Tanzania; and National Institute of Health & Maputo Central Hospital in Mozambique). The project is aimed at further optimization of DNA vaccine delivery, and the planned activities will include a prolonged follow up of HIVIS03 as well as a new Phase II HIV vaccine trial to be conducted in Dar es Salaam and Mbeya. There will also be preparatory activities for conducting HIV vaccine trials among youths in Tanzania and Mozambique. Funds have been secured for the extension of the HIVIS trial to take care of the delay in MVA availability for the second boost and subsequent safety and immunogenicity assessments.

Preliminary work on HIV vaccine trials in Dar es Salaam
Tanzania was among the first countries assessed by WHO in 1994 and recommended to be considered as a potential country for future HIV/AIDS vaccine trials. To that end, a cohort of police officers was recruited to assess its suitability for future HIV vaccine trials by determining the prevalence and incidence of HIV-1 infections. The number of police officers in Dar es

Salaam is around 15000 and they have a relatively low turn over. The overall HIV-1 sero-prevalence at recruitment among 2733 police officers recruited in 1994-1996 was found to be 13.8%. The overall crude HIV-1 incidence was 19.6/1000 PYAR, a figure that is within the 10-30/1000 PYAR interval recommended by the WHO for efficient vaccine trials (3). These results indicated that the police officers cohort is a potential suitable population for vaccine trials.

Tanzania's National HIV Vaccine Strategic Framework
It is of note that the HIVIS Project was conceived as a consequence of the first National HIV Vaccine Workshop in Tanzania that took place in July 2001 at the Bahari Beach Hotel, Dar es Salaam, which received support from the WHO-UNAIDS HIV Vaccine Initiative, the European Union, the Walter Reed Army Institute and the Henry M. Jackson Foundation both of the U.S.A. Government.

A further workshop to develop the Tanzania National HIV Vaccine Framework was conducted in mid-September 2004. This workshop was supported by, among others, the WHO/UNAIDS, and the African AIDS Vaccine Programme (AAVP).

The framework aims to guide the development and evaluation of HIV vaccines in the country, with a vision being to ensure availability of effective, safe, affordable and accessible HIV vaccines for the benefit of all Tanzanians. The mission is to promote research, development, production and evaluation of suitable HIV vaccines and ensure sufficient availability of the vaccine in an equitable way for the entire needy population of the country, through national, regional and international collaboration. The plan has now been finalised and was officially presented to the MOH in Jan 2005 for further action.
As a further consequence, the Ministry of Health is in the process of facilitating the formation of Tanzania's HIV Vaccine Initiative (TAVI) to include representatives from institutions and stakeholders involved in HIV vaccine Research and Development in the country.

OBJECTIVES
The objectives of the current proposal are as follows:

Primary objectives

1. To assess the safety of a plasmid DNA-MVA prime boost HIV-1 vaccine candidate among volunteers in Dar es Salaam, Tanzania.

2. To determine the immunogenicity of a plasmid DNA-MVA prime boost HIV-1 vaccine candidate among volunteers in Dar es Salaam, Tanzania.

Secondary Objectives

1. To build expertise and capability in evaluating HIV-1 vaccines in Dar es Salaam, Tanzania

2. To evaluate the immunogenicity of plasmid DNA-MVA prime boost HIV vaccines on the background of antibodies against Vaccinia virus

METHODOLOGY

Study design
This is a randomised, controlled, double blinded study. The laboratory staff will also be blinded to the immunization regimens.

Setting
The clinical site will be MNH/MUHAS, Dar es Salaam, TANZANIA. Laboratory tests will be performed at the Department of Microbiology and Immunology, MUHAS, Dar es Salaam, and at the Swedish Institute of Infectious Disease Control and Karolinska Institute, Stockholm, SWEDEN

Duration of study
The enrolment took place between 20th Feb 2007 and 26th Feb 2008.Volunteers will be monitored up to 24 weeks after the last injection

Study volunteers
All the 60 volunteers for the study were recruited from the Police Force in Dar es Salaam, with whom we have had extensive contact and collaboration since 1994.
Fifteen out of the 60 (25%) enrolled volunteers, are females. They were all HIV negative by ELISA HIV antibody/antigen assays at the time of enrolment, and they were considered to be at low risk for HIV acquisition at the time of enrollment.

Eligibility (Inclusion) criteria

1. Age: 18 to 40 years
2. Willing to undergo counseling and HIV testing
3. Have a negative antigen/antibody ELISA for HIV infection
4. Able to give informed consent
5. Literacy corresponding to a minimum of 7 years of primary education.
6. Resident in Dar es Salaam, and willing to remain so for the duration of the study
7. At low risk of HIV infection, defined as the absence of an identifiable risk factor/ behaviour:
    - sexual partner with HIV
    - sexual partner with unknown HIV serostatus who is also unwilling to use protective condoms consistently in all sexual relations
    - sexual partner is known to be at high risk for HIV
    - more than one sexual partner in the last 6 months.
    - History of being an alcoholic [as medically defined or more than 35 units /week]
    - History of STI within past 6 months.
8. Verbal assurances that adequate birth control measures are used not to conceive/father a child during the study and up to 4 months after the last vaccine injection
9. Have a negative urinary pregnancy test
10. Be willing to practice safe sex for the duration of the study to avoid sexually transmitted infections including HIV.
11. Good health as determined by medical history, physical examination, clinical judgment and by key laboratory parameters

Reference ranges will be in accordance with data generated at MUHAS for hematology values, and that generated at Mbeya (MMRP) for biochemical parameters. Exclusion by presence of Diabetes mellitus will be based on the WHO cut-off value of a Fasting Blood Glucose <7.8 mmol/l

No grade 1 or higher routine laboratory parameters (see section on **appendix xv** for Definitions):

- Hb >10.5g/dl
- White blood cell count >1,300/mm^3
- Granulocytes >6.4/ mm^3
- Lymphocytes >1.0/ mm^3
- Platelets >120,000/ mm^3
- CD4 >400cells/ mm^3
- Random Blood Glucose 2.5-7.0 mmol/L; if elevated, then a Fasting Blood Glucose <7.8 mmol/l
- Bilirubin <1.25 x uln
- ALT <1.25 x uln
- Creatinine <1.25 x uln
- Urine dipstick for protein and blood: negative or trace. (If either is ≥ 1+, obtain complete urinalysis (UA). If microscopic UA confirms evidence of hematuria or if proteinuria ≥ 1+, the volunteer is ineligible).

Exclusion criteria

The following are the criteria for exclusion from the study:

1. Active tuberculosis or other systemic infectious process elicited by review of systems, physical examination and laboratory detection. Such as detection of Hepatitis B surface antigen, or active syphilis.
2. Have a history of immunodeficiency, chronic illness requiring continuous or frequent medical intervention
3. Autoimmune disease by history and physical examination.
4. Severe eczema
5. Have history of psychiatric, medical and/or substance abuse problems during the past 6 months that the investigator believes would adversely affect the volunteer's ability to participate in the trial.
6. History of grand-mal epilepsy, or currently taking anti-epileptics
7. Have received blood or blood products or immunoglobulins in the past 3 months.
8. Are receiving immunosuppressive therapy such as systemic corticosteroids or cancer chemotherapy.
9. Have used experimental therapeutic agents within 30 days of study entry.
10. Have received any live, attenuated vaccine within 60 days of study entry. {NOTE: Medically indicated subunit or killed vaccines (e.g., Hepatitis A or Hepatitis B) are not exclusionary but should be given at least 2 weeks before or after HIV immunization to avoid potential confusion of adverse reactions}.
11. Have previously received an HIV candidate vaccine.
12. History of severe local or general reaction to vaccination defined as:
    - **Local**: Extensive, indurated redness and swelling involving most of the major circumference of the arm, not resolving within 72 hours
    - **General**: Fever >= 39.5 ^{0}C within 48 hours; anaphylaxis; bronchospasm; laryngeal oedema; collapse; convulsions or encephalopathy within 72 hours
13. Are lactating mothers
14. Are study site employees who are involved in the protocol and may have direct access to the immunogenicity results
15. Unlikely to comply with protocol as judged by the principal investigator or his designate.

RECRUITMENT PROCEDURE

Recruitment

As aforementioned, volunteers will be recruited from a cohort of Police Officers' (PO's) volunteers in the on-going HIV incidence and socio-behavioural studies. In brief, in these studies a total of 3000 volunteers are expected to be enrolled. From amongst them, a "core group" of about 300 PO's will be formed who will assist with educational activities on HIV/AIDS, and HIV vaccine in the force as well as recruitment of the 60 volunteers.
Meetings at the Police stations, efforts by the "core group" of collaborators and a one to one contact, are the main modalities to reach potential volunteers. During the recruitment volunteers will be provided with detailed information about the study and will be given an information sheet (***Appendix i, Form 2-I or II***). In the next visit if they are still interested and willing to participate, they will be invited to attend for screening, whereby a check list of items (appendix iii, Form 1), will be employed. At this point they will be given a personal identification four-digit Medical Record Number (MRNO).

The study site for the successive visits and HIV vaccinations will be a building where the HIV clinic at MNH used to run (Makuti). It is expected that by the time the study starts, the HIV clinic will have shifted to the new outpatient department building sponsored by Axios International with funding from the Abbot Laboratories, Inc.

Likewise, recruitment will be conducted on an individual basis and by personnel without ties to the particular group involved; should any of the other alternative sources of potential volunteers (listed above) be considered. As for police officers, voluntariness will be emphasized during recruitment.

Screening

The first screening visit (study visit 1) will take place between 4 and 8 weeks before the first planned injection. At this visit the study volunteer will meet the study doctor(s) and study nurse(s), and the trial will be discussed in detail. This discussion will also address on the following: the meaning of 'randomization', potential adverse effects of the vaccine, about the fact that it is unknown whether or not the study vaccines will protect him/her against HIV infection. They will be informed that following immunization they may develop antibodies that will produce a positive reaction in a routine HIV test, but that provisions have been made to distinguish between a post vaccination response and HIV infection during and after the trial. That volunteer will be excluded from donating blood or organs and that they may be vulnerable to social risk if they develop HIV antibodies, or by revealing their participation in the study. They will be assured of the level of care that will be made available to them should they be found to be HIV infected at any time during their participation in the study, including the screening period. The placebo concept will also be explained. Study doctor or nurse will use a checklist of items (***Appendix v {Form 1}***) as a guide in order to make sure that all issues are discussed.

Volunteers will be counseled by study personnel about safe sex. Volunteers will be counseled on the importance of contraception from starting immunization up to four months after the last immunization. That volunteer should continue to use condoms with sexual partners whose HIV status is not known. They will also be reminded about these items on each day of immunization. High quality hypo-allergenic condoms will be provided free of charge to volunteers throughout the trial.

Upon this information about the trial, volunteers will be requested to sign part 1 of the written informed consent for screening (***Appendix ii/iv (CRF's 4-I/II)***). Screening will involve the following procedures:

a. Clinical history and examination

Medical status and medical history, including any ongoing medication and any previous allergic reactions, any previous reaction to vaccination, history of epileptic fit, exposure to smallpox vaccine, and smoking practices. All these will be documented in study form CRF 1-I (***Appendix vi***). The general examination will include weight (kg), height (cm) and arm circumference, mouth and throat inspection, palpation of lymph nodes (cervical, clavicles, and axillaries), blood pressure, and inspection of the skin to exclude severe eczema and check for the presence of a vaccinia scar, respiratory, cardiovascular, genital urinary, central nervous system and gastrointestinal system examination. These will be filled in CRF 1-II (Appendix vii)

b. HIV counseling and testing

Study personnel will assess volunteers for past and current risk of HIV infection using the screening questionnaire, followed by counseling for those who will consent to undergo HIV test prior to collecting blood. The counseling process will ensure that volunteers have sufficient knowledge about HIV infection to understand what the test is for, the implications of a positive, negative and equivocal result and the standard of care available for HIV infection locally. They will also be informed on how and when they will receive the result.

After a volunteer is found to be eligible, the screening number will be used as the study number. This means that study numbers may be discontinuous. After signing written study consent the study volunteer will be invited to donate blood for safety laboratory tests including determination of HIV status.

c. Safety laboratory tests

HIV testing

All volunteers will have their blood sample screened for HIV using Vironostika/Murex antigen antibody combined assay (Biomerieux, Netherlands/Abbot Murex, UK). Non-reactive samples on the first ELISA will be regarded as negative. All reactive samples will be confirmed by antibody based assay Enzygnost anti-HIV-1/2 Plus (Dade Behring, Germany). Discrepant results between two ELISAs will be resolved by Innolia immunoblot assay (Innogenetics, Belgium).

Those found to be HIV negative will be evaluated on the status of the liver and renal functions and random blood glucose that will be followed by fasting blood sugar for individuals whose tests will be suspicious of diabetes mellitus. Other tests will include a full blood picture, screening for syphilis, hepatitis B (surface antigen) infection and immunological tests (CD4 counts and percentage) as well as urine dipsticks. All women will have a urine pregnancy test done.

Genital infection screening

The following will be collected if indicated on account of risk and symptoms, according to trial specific clinical standardized operating procedures: urethral, vaginal, cervical specimens, as appropriate for common sexually transmitted infections.

Blood and urine collection

Blood will be collected using a sterile needle, usually from the ante-cubital fossa, according to the schedule and transported to the appropriate laboratories. Urine will be collected into a sterile container as indicated on the schedule and either transported to the appropriate laboratory or tested by a member of the clinical team according to trial specific standardized operating procedures.

The total estimated volume of blood to be collected at different time points is shown on the investigations flow chart at each scheduled visit. All biological samples to be analysed by the clinical laboratory will be collected and processed according to institutional guidelines and regulations regarding biobanks. All samples sent to the appropriate laboratories at MUHAS will be labelled with the following information: Medical record number, volunteer initials, study visit (week), visit number, date and time of collection, type of sample (serum, plasma, blood etc).

All participants will be given a chart with planned attendance dates so that they can be adjusted to the participant's availability, ie +/- 1 day of the ideal.

All samples for routine haematology, biochemistry and other investigations will be labelled appropriately with the full study volunteer identity. Samples that will be analysed after freezing shall be prepared, labelled, and stored at the appropriate temperature. Specimens that are shipped will be correctly prepared, labelled, and kept at the correct temperature.

The **second screening visit** (study visit 2) takes place two to four weeks before the first planned injection. This visit will include review of all laboratory results. At this visit the study nurse confirms that the volunteer fulfils all the inclusion criteria, including laboratory results, makes an abbreviated risk assessment and a brief summary of the study design. Reassurance has to be made that the study volunteer fully understands the concept of the study. The results of the screening investigations will be reviewed and volunteers who are still eligible and willing will be asked to donate blood samples for baseline immunogenicity and complete part 2 of the Informed consent (***Appendix ii/iv, CRF 4-II***). Volunteers will also be reminded that they are free to end all obligations in the study whenever they wish and that a premature termination will not have any negative influence in future contacts with the clinic. If no contraindications are revealed volunteer will be asked to donate blood for first baseline immunogenicity tests.
The volunteer will then be randomized into one of the study arms. At randomization a total of sixty sealed plain envelopes containing an equal number of assignments to groups I, II and IIIa & IIIb, will be prepared and put in the box. Group numbers refer to the immunization schedule. The study Pharmacist will take the prepared envelopes and put them in the box that is kept under lock. Volunteers accepted into the study wll be randomized by a Pharmacist through CRF7-I, by selecting the next consecutive envelope containing the vaccine or placebo assignment in accordance with the generated randomization list. This will be documented in CRF 7-II.
The Pharmacist will then inform the Study Nurse at the clinic of the randomization group of the volunteer (ID or IM) on a copy of CRF 7-I, including a number of the envelope, but will not inform on whether this is vaccine or placebo. The Study Nurse will record the envelope number and the IM/ID assignement into a Register book, the medical record file (source document), as well as the outer cover of the volunteer's file.
The pharmacist will then deliver to the nurse the appropriate vaccine/placebo that corresponds to the assigned group.
Recruitment of volunteers will be staggered so that a maximum of 3 volunteers per week will enter the trial during the first 5 weeks.

Study Procedures
At **week 0 (study visit 3)** the study volunteer again meets both study nurse(s) and study doctor(s). Once again confirmation has to be made that the volunteer fulfils all inclusion criteria, including laboratory results, and fully understands the concept of the study. After doing so blood is donated for second baseline immungenicity tests. Urine for pregnancy test will be performed and found negative.

The volunteer will then receive the first injection according to the assigned study arm. The study visit will be documented in study form CRF 8-I (***Appendix xii***). Following injection volunteers will be observed for 30 minutes for acute adverse reactions and will also be contacted the day following injection by the study nurse for a brief adverse reaction interview. In addition, volunteers will complete diaries (***Appendix xvii {Form 7}***) over events for 7 days following each vaccination and receive telephone numbers to call 24 hours a day, in order to get in contact with the study personnel if needed. A folder with diary cards, disposable thermometers and a ruler will be issued to each volunteer.

Blinding procedure & Study visits
The vaccines will be kept in the department of Microbiology/Immunology, MUHAS and the pharmacy under appropriate storage conditions according to manufacturer's specifications. One identified pharmacist who has access to the code will draw the study vaccine and the reference vaccine into syringes and label them with three initials and study number and hand them over to the study nurse for injection. All samples from the volunteers will be labeled using three initials and study numbers. Nurses administering the vaccines together with all other investigators who will be involved in the follow up process as well as the volunteers themselves will be blinded to the type of vaccine that the volunteer will be receiving.

Week 2 (study visit 4) is conducted by the study nurse(s) and the study doctor. Study form CRF CRF 8-II is filled in and blood donated according to flow chart. Any medications taken will be filled in CRF 9. Abbreviated risk assessment and risk behaviour counseling will be done.

Week 4 (study visit 5) the visit for the second injection of the study vaccine, is conducted by both the study nurse and the study doctor. Before injection the diaries over events after the last injection will be discussed with the study volunteer and an abbreviated risk assessment and risk behaviour counseling will be performed. Urine for pregnancy test will be performed and found negative. Enough time has to be planned for the visit so that all questions from the volunteer about the study can be answered or reflected upon. After blood donation the second injection is given and the visit is documented in study form CRF 8-II. Again volunteers will be observed for 30 minutes following injection and new diaries over events will be issued for recording events over 7 days.

Week 6 (study visit 6) is conducted by the study nurse(s) and the study doctor. Study form CRF CRF 8-II is filled in and blood donated according to flow chart. Abbreviated risk assessment and risk behaviour counseling will be done.

Week 8 (study visit 7) is conducted by the study nurse(s) and the study doctor. Diaries over events after the last injection will be discussed with the study volunteer. Study form CRF 8-II is

filled in and blood donated according to flow chart. Urine for pregnancy test will be performed and found negative. Abbreviated risk assessment and risk behaviour counseling will be done.

Week 12 (study visit 8), the visit for the last DNA injection before MVA-boost, is conducted by both the study nurse and the study doctor. Before injection the diaries over events after the last injection will be discussed with the study volunteers and an abbreviated risk assessment and risk behaviour counseling will be performed. Urine for pregnancy test will be performed and found negative. Enough time has to be planned for the visit so that all questions from the volunteers about the study can be answered or reflected upon. After blood donation the third injection is given and the visit is documented in study form CRF 8-II Again volunteers will be observed for 30 minutes following injection and new diaries over events for 7 days will be issued.

Week 14 (study visit 9) is conducted by the study nurse(s) and the study doctor. Study form CRF 8-II is filled in and blood donated according to flow chart. Abbreviated risk assessment and risk behaviour counseling are done.

Week 16 (study visit 10) is conducted by the study nurse(s) and the study doctor. Diaries over events after the last injection will be discussed with the study volunteer. Study form CRF 8-II is filled in and blood donated according to flow chart. Abbreviated risk assessment and risk behaviour counseling will be done.

In between study visit 10 and 11 we have at least four visits 'Pre MVA' aiming at keeping in touch with the volunteers. During the visits counseling is provided and evaluation of safety is done clinically.

Week 36 (study visit 11), the visit for the first MVA injection of the study vaccine, is conducted by both the study nurse and the study doctor. Before injection the diaries over events after the last injection will be discussed with the study volunteers and an abbreviated risk assessment and risk behaviour counseling will be performed. Urine for pregnancy test will be performed and found negative. Enough time has to be planned for the visit so that all questions from the volunteers about the study can be answered or reflected upon. After blood donation and performance of a baseline ECG these will be documented in CRF 6. The MVA injection is then given and the visit is documented in study form CRF 8-II. Again volunteers will be observed for 30 minutes following injection and new diaries over events for 7 days will be issued.

Week 38 (study visit 12) is done 2 weeks post 1st MVA. It is conducted by the study nurse(s) and the study doctor. Study form CRF 5 is filled in, a post-MVA ECG is done and documented in CRF 6. Clinical assessment for safety is performed, and CRF 8-II is filled in. Blood is donated according to flow chart for safety and immunogenicity. Abbreviated risk assessment and risk behaviour counseling are done.

Week 40 (study visit 13), is done one month post 1st MVA/placebo. It is conducted by the study nurse(s) and the study doctor. Diaries over events after the last injection will be discussed with the study volunteers. Study form CRF 8-II is filled in to document clinical assessment. Blood is donated according to flow chart for safety and immunogenicity. Abbreviated risk assessment and risk behaviour counseling are done

Week 44 (Study visit 14) is done two months post 1st MVA/placebo. It is conducted by the study nurse(s) and the study doctor. Diaries over events after the last injection will be discussed with the study volunteers. Study form CRF 8-II is filled in to document clinical assessment. Blood is donated according to flow chart for safety and immunogenicity. Abbreviated risk assessment and risk behaviour counseling are done

Week 60 (Study visit 15) is done six months post 1st MVA/placebo. It is conducted by the study nurse(s) and the study doctor. Diaries over events after the last injection will be discussed with the study volunteers. Study form CRF 8-II is filled in to document clinical assessment. Blood is donated according to flow chart for safety and immunogenicity. Abbreviated risk assessment and risk behaviour counseling are done

The **Study visit (visit 16)** is performed at week 64. Again the study doctor takes a complete medical history and performs a physical examination according to study visit 1. An extended risk assessment and risk behaviour counseling is performed. i

Volunteers will be be asked to participate in long-term follow-up studies. For the time being this will entail participating in a study that will involve giving a 2nd MVA/placebo boost
Consent will be requested from each of the the volunteers for this extension of the trial. The 2nd MVA/placebo will be given 44 weeks after the 1st MVA/Placebo. Pregnancy and HIV infection will be excluded.
Safety and Immunogenicity will be assessed at 2 weeks, 1 month, 2 months and 6 months post 2nd MVA.

Between the 1st and 2nd MVA/Placebo vaccinations consenting volunteers will attend the clinic at 8 weeks intervals with a standard follow up. Whenever appropriate, volunteers will also be invited to meetings with the investigators and representatives of the Comminity Advisory Board aimed at emphasing low risk behavior towards HIVand AIDS as well as giving updates on matters relating to HIV vaccine trials and HIV and AIDS in general.
It will also be an opportunity to maintain contacts with the volunteers and enable the volunteers to share experiences on being in the trial.

Week 76 (study visit 17), this visit will be held four weeks before the 2nd MVA/placebo vaccination, for those who would have given consent during visit 16. Blood will be donated for ELISA HIV testing and for HIV DNA or RNA PCR for those found to have HIV antibodies by ELISA in order to determine if seropositivity is due to HIV acquisition or is a result of candidate HIV vaccine immunogenicity.

Week 78 (study visit 18), this visit will be held two weeks before 2nd MVA vaccination. Volunteers will be given their HIV test results, and those found HIV negative will be invited for 2nd MVA at visit 19. Abreviated risk assessment and appropriate counseling will be conducted

Week 80 (study visit 19), the visit for the second MVA injection of the study vaccine, is conducted by both the study nurse and the study doctor. Before injection the diaries over events after the last injection will be discussed with the study volunteers and an abbreviated risk assessment and risk behaviour counseling will be performed.

Urine for pregnancy test will be performed on female volunteers .
Enough time has to be planned for the visit so that all questions from the volunteers about the study can be answered or reflected upon. Blood will be donated for immunogenicity. The MVA injection is then given and the visit is documented in study form CRF 8-II. Again volunteers will be observed for 30 minutes following injection and new diaries over events for 7 days will be issued.

Week 82 (study visit 20) is done 2 weeks post 2nd MVA. It is conducted by the study nurse(s) and the study doctor. Study form CRF 5 is filled in, a post-MVA ECG is done and documented in CRF 6. Clinical assessment for safety is performed, and CRF 8-II is filled in. Blood is donated according to flow chart for safety and immunogenicity. Abbreviated risk assessment and risk behaviour counseling are done.

Week 84 (study visit 21), is done one month post 2nd MVA/placebo. It is conducted by the study nurse(s) and the study doctor. Diaries over events after the last injection will be discussed with the study volunteers. Study form CRF 8-II is filled in to document clinical assessment. Blood is donated according to flow chart for safety and immunogenicity. Abbreviated risk assessment and risk behaviour counseling are done

Week 88 (Study visit 22) is done two months post 2nd MVA/placebo. It is conducted by the study nurse(s) and the study doctor. Diaries over events after the last injection will be discussed with the study volunteers. Study form CRF 8-II is filled in to document clinical assessment. Blood is donated according to flow chart for safety and immunogenicity. Abbreviated risk assessment and risk behaviour counseling are done

Week 104 (Study visit 23) is done six months post 2nd MVA/placebo. It is conducted by the study nurse(s) and the study doctor. Diaries over events after the last injection will be discussed with the study volunteers. Study form CRF 8-II is filled in to document clinical assessment. Blood is donated according to flow chart for safety and immunogenicity. Abbreviated risk assessment and risk behaviour counseling are done

Week 106 (Study visit 24) this is the final study visit. Again the study doctor takes a complete medical history and performs a physical examination according to study visit 1. An extended risk assessment and risk behaviour counseling is performed.

Volunteers will be thanked and could be asked to participate in a longer-term follow up if by then this will be deemed necessary.All study volunteers will be issued a card signed by the principal investigator (full names, tel. numbers and addresses) stating that the holder has participated as a volunteer in a HIV-1 immunization study and that the outcome of standard HIV diagnostic tests if found positive may not not signify HIV-1 infection until special tests i.e western blot and HIV PCR are conducted to distinguish between true HIV infection and vaccine-induced seroconversion.

Immunizations
Immunization schedule

While DNA immunizations will be performed at weeks 0, 4 and 12. 1st MVA will be given at weeks 36 and the 2nd MVA will be given at week 80 The immunization schedule is summarized below:

Weeks	-4-8	-2-4	0	2	4	6	8	12	14	16	36	38	40	44	60	64	76	78	80	82	84	88	104	106
Visits	1	2	3	4	5	6	7	8	9	10	11	12	13	14	15	16	17	18	19	20	21	22	23	24
Type of immunization			DNA1		DNA2			DNA3			MVA 1					EXIT 1			MVA 2					EXIT 2

Arm	Volunteers	DNA immunization	MVA boost
**I**	20	DNA intramuscularly by Bioject	MVA 10^8 i.m
**II**	20	DNA id by Bioject	MVA 10^8 i.m
**IIIa**	10	Saline intramuscularly by Bioject	Saline i.m
**IIIb**	10	Saline id by Bioject	Saline i.m

Doses

Vaccine and delivery method	Route	Ampoule 1 (env), Left arm	Ampoule 2 (gag, Rtmut), Right arm
DNA by Biojector	Intramuscularly	1.0 ml (tot 2.0 mg DNA)	0.9 ml (tot 1.8 mg DNA)
DNA by Biojector	Intradermally	3 injections of 100ul (Total 0.6mg DNA)	2 injections of 100ul (total 0.4mg DNA)

Mode of Vaccine Delivery

All DNA immunizations will be performed with the Bioject device according to the manufacturer's instruction. Intramuscular injections will be given in the deltoid muscle. Ampoule 1 will be given in the left arm and ampoule 2 in the right arm. During the procedure the overlying skin will be stretched flat prior to applying the Bioject device. To ensure that the fluid reaches the muscle, and that the product does not leak into the surrounding tissues, a decision about the orifice size required will be made individually for each participant, based on published recommendations [29, 30]. Intradermal injections will be given in the skin over the deltoid muscle with the extension supplied by the manufacturer for this purpose. Ampoule 1 will be given in the left arm and ampoule 2 in the right arm. Ten minutes after immunisation, the site will be inspected and any local reactions recorded on the case record form.
The MVA-boost injection will be delivered intramuscularly in the deltoid muscle.

The immunisations will take place in an outpatient setting. Participants will be closely observed for 30 minutes after each immunisation, at which point vital signs (pulse, blood pressure and respiratory rate) will be recorded on the case record form, as will any local or systemic reaction. The needles, syringes and other materials will be disposed of according to MNH biohazard regulations. As with any parenteral vaccine, epinephrine and corticosteroids will be available for immediate use should an immediate hypersensitivity reaction, such as anaphylaxis, occur. Intravenous injections of vaccines will NOT be performed. Study vaccine will not be administered to individuals with hypersensitivity to any component of the vaccine. All immediate hypersensitivity reactions will be managed according to the developed SOP's. Volunteers will also be contacted 1-3 days at the clinic following injection for a brief adverse reaction interview. In addition, volunteers will complete diaries over events for 7 days following each vaccination.

Safety follow-up in the days following immunization
A diary card will be given, with instructions and a full verbal explanation, for volunteers to record local and systemic adverse events following immunization, as well as medication taken. Volunteers will be observed on the day of immunization as above, and contacted within 3 days following injection for a brief adverse reaction interview at the clinic. In addition, volunteers will complete diaries over events for 7 days following each vaccination. Contact will be maintained by the clinical team, using a method chosen by the volunteer, as indicated by the evolution of adverse events, up to resolution of solicited local and systemic events. Additional visits may be recommended at the discretion of the clinical and principal investigators, if clinically indicated or in order to clarify observations. All concomitant medication must be recorded in study form CRF 9 (***Appendix xiii***) with the reason for administration, the dosage regimen, the onset and end of treatment.

The following medications are prohibited while the volunteer is in the study:
- Immunomodulatory agents (i.e. immunoglobulin)
- Immunosuppressive agents (i.e., systemic steroids, chemotherapy)
- Live, attenuated vaccines. Other vaccines (subunit or killed) should be given at least two weeks before or two weeks after HIV immunization.

Final visit
Assessments will be undertaken according to schedules as depicted above.

Unscheduled visits
If the volunteer attends or contacts the clinic for any health related changes or event following immunization, a CRF 12 will be filled in, and where appropriate, an adverse event form (CRF 8-1 and CRF 8-II) will be filled in.

REIMBURSEMENT
Regular payments will be made to all volunteers to cover their travel expenses (TShs 5,000.00 x 2) as well as time and inconvenience (TSh 10,000.00) compensation. This will amount to 20,000.00 T.Shs (approx=17.00 USD) per visit. During the study, volunteers will receive hypoallergenic latex condoms free of charge. If any medication is required as a result of the study, this will also be provided free of charge. Such medication will include painkillers, antipyretics and the like. In case of hospitalization/or if further care will be required, these will be covered under the medical insurance; all these will be confined to the period of the study.

IN THE EVENT OF DISCONTINUATION

Study volunteer may withdraw his/her consent to participate in the study at any time without prejudice. Wherever possible, the tests and evaluations listed for the termination visit will be carried out if the volunteer refuses follow-up according to the protocol visit schedule. The Data and Safety Monitoring Board (DSMB) will be notified of all study withdrawals within 24 hours. Volunteers who withdraw from the study will be replaced only as long as the study is still open for enrolment. No replacement will be allowed when the enrolment is closed. All volunteers who receive at least one immunization will be included in the safety analysis. If a volunteer does not complete the immunization schedule secondary to a serious adverse event or toxicity, he or she will continue to be followed according to the protocol visit schedule, *and, at* a minimum, until the adverse event/toxicity is resolved and/or the cause is identified.

A genuine effort will be made to determine the reason(s) why a volunteer fails to return for the necessary visits. This information will be recorded on the appropriate source document, exit Form CRF 11.

Pregnancy reporting and follow up

For volunteers who become pregnant before completing the vaccine series, no further immunizations will be given. A positive UPT test result will be communicated to the volunteer and her ineligibility to continue with the study. A pregnancy report form CRF 10, the exit form CRF 11 and an adverse event CRF 8-II are filled in. The volunteer will be followed for all the remaining scheduled visits according to the schedule of procedures for safety evaluation, as well as her antenatal clinic for the maternal outcomes during pregnancy, labor, delievery,and post-delivery periods. The infant outcome will also be sought.

THE VACCINE (TRIAL PRODUCTS)

DNA Priming

Will be effected with DNA plasmids derived from puC8 with a kanamycin resistance gene, hCMV promotor, HPV 16 poly A and origin of replication for *E. coli*. They will be carrying HIV-1 genes of subtypes A, B and C. These are pKCMV**gp160**A, KCMV**gp160**B, pKCMV**gp160**C, pKCMV**rev**, pKCMV**p37**A(ba), pKCMV**p37**B, and pKCMV**pRT**B.

Supply, storage and composition of DNA

Vecura company (Karolinska University Hospital at Huddinge, Stockholm, Sweden) will be responsible for bulk manufacture of the DNA vaccine, release testing and technical release of vialled product and labelling. All procedures will be according to Good Manufacturing Practice. The presentation is in liquid form, with an extractable volume of 1.0 ml and 2ml vials, which should be stored at –20 ^{0}C until use. Care will be taken not to break the cold chain

The DNA plasmids were approved for human use on November 27th 2004 by the Swedish Medical Products Agency, (läkemedelsverket), and the second revision of the protocol ethically cleared by the Swedish Internal Review board, (Regionala forskningsetiska kommitten) December 2004, and will need to have a further approval by the Tanzania Food and Drugs Authority (TFDA)

Labels

DNA will be packed by Vecura. The trial products will be in vials pre-labelled according to standardised operating procedures. Each vial will be packed in a box labelled with the name of the clinical site and principal investigator, the storage details and the name of the supplier for the product. Cartons will be supplied to the designated pharmacist at MNH for storing the used vials, labelled with the supplier, the name of the trial and protocol version; the volunteer study number; and the name of the clinical site and principal investigator.

Having been manufactured by Vecura Company, the DNA vaccine will be supplied to the study by the Swedish Institute for Infectious Disease Control, 171 82 Solna. SWEDEN.

Prior to use the vials will be thawed at room temperature. When completely thawed the vials will be gently swirled. Care will be taken not to shake or invert the vials.

MVA Boosting

Boosting will be effected by a Modifed Vaccinia Ankara vaccine (MVA-CMDR). The MVA - CMDR, produced by WRAIR/NIH, is a live recombinant poxvirus vector vaccine that has been genetically engineered to express the following HIV-1 genes:

1. **gp**160 (Subtype E, CM235), and
2. **gag** and **pol** (integrase-deleted and reverse transcriptase nonfunctional, Subtype A, CM240).

MVA-CMDR is formulated in a liquid form in vials and will be administered at a dose of 10^8 pfu by intramuscular route following appropriate dilutions.

Supply, storage and composition of MVA

Walter Reed Army Institute of Research (WRAIR), USA, will be responsible for the bulk manufacture of the clinical material, release testing, as well as the technical release of vialled product, and labeling. The full address of the manufacturer for the MVA-DNA used for the 1st boost is: Walter Reed Army Institute of Research (WRAIR), Forest Glen Section, Building 501, Department of Biologics Research The Pilot Bioproduction Facility, Silver Spring, MD 20910, USA The MVA-DNA to be used for the 2nd boost is produced by Advanced Biosciences Laboratories, Inc. Kensington, Maryland, 20895-1078, USA using the same seed lot as for the 1st boost All procedures will be according to Good Manufacturing Practice. The presentation is in liquid form, with an extractable volume of 1ml vials, which should be stored at -20 ^{0}C until use. Care should be taken not to break the cold chain. The product has already received approval from the USA Food and Drugs Authority (FDA), and the Swedish Medical Produtcs Agency and will be presented to the TFDA for approval before use in Tanzania.

Dispensing records and disposal of unused product

The designated pharmacist will, upon receipt of supplies prior to commencement of the trial, conduct an inventory and complete a receipt, one copy of which will be retained at the site, and the original returned to the supplier (Karolinska Institute, Sweden). During the trial the pharmacist will be responsible for reviewing the dispensing log. On the day of immunization the study nurse will fill in the request form with the study number, date of birth, and immunization number. Upon receipt of the syringes with the vaccine from the pharmacist, the study nurse will deliver the vaccine. The immunization number and date will be entered against the study number in the dispensing log. The vial label will be cross-checked against the details on the prescription and dispensing log by two individuals (pharmacist and nurse), prior to the vaccine/placebo being administered.

The Pharmacist will be responsible for ensuring that the return of used vials is recorded in the dispensing log at the end of the clinical session, and that they are placed in the appropriate volunteer carton. At the end of the trial all used and unused vials will be checked against the inventory by the monitor before disposal on site according to MNH biohazard regulations. Documentation of disposal will be provided to the monitor and supplier. During the trial, product accountability will be monitored by the prescriptions, the dispensing log, the returns, the trial register and data collected on the clinical research forms.

END POINTS

Primary

Safety
The safety of immunization with the seven DNA plasmids carrying HIV-1 genes will be assessed by *clinical features as well as standard biochemical and haematological laboratory tests*. Any worsening of the severity grade will be considered for causality with the vaccine.

The alternative routes of immunization (intramuscularly and intradermally with the Bioject device) will be evaluated by assessing local (pain, cutaneous reactions including induration), general (fever, chills, headache, nausea, vomiting, malaise, myalgia) and other unsolicited adverse events on vaccination day and within 7 days. Any grade 3 or 4 event will be taken as an indication that the arm is less tolerable.

The primary safety parameters will be graded according to ***appendix xiv***, and are:
1. Local adverse event Grade 3 or above (pain, cutaneous reactions including induration)
2. Systemic adverse event Grade 3 or above (temperature, chills, headache, nausea, vomiting, malaise, and myalgia)
3. Other clinical or laboratory adverse event Grade 3 or above confirmed at examination or on repeat testing respectively

Tests for possible toxicity due to vaccination will include: Liver function tests, renal function tests and full blood picture. Any event attributable to vaccine leading to discontinuation of the immunization regimen must be documented.

Data on local and systemic events listed above will be solicited with specific questions or using a diary card for a minimum of 7 days following each immunization. Data on other clinical events and laboratory events will be collected with an open question at each visit and through routine scheduled investigations respectively.

Immunogenicity
Responses to vaccination will be assessed by humoral and cell mediated tests. The assessment of humoral response will be by the determination of specific binding and neutralizing antibodies against the vaccine.

The qualitative and quantitative evaluations of cell mediated immunogenicity will be through the determination of interferon gamma by the ELISPOT assay; whole blood lymphocyte proliferation assay (FASCIA); intracellular cytokine staining (ICCS) using flow cytometry as well as cytotoxic T lymphocytes (CTL) assays against peptides of the different immunogens. A detailed description of safety and immunogenicity assessments is depicted below.

SAFETY AND IMMUNOGENICITY ASSESSMENTS

SAFETY ASSESSMENTS
Local and systemic assessments whether normal or abnormal will be recorded starting from 10 minutes up to 4 weeks after immunization in the following intervals: 10 and 30 minutes as direct observation after immunization, day 1-3 (at the clinic or by home visit), 2nd and 4th week at

clinic. While study personnel will use {CRF 8 (I-III)} to record all local and systemic events at the clinic, volunteers will use diary cards at home for the same. Volunteers who will not report to the study clinic within the first three days will be home visited within a week. Volunteers will be instructed to call or report to the specified study personnel immediately if any unusual or severe sign or symptom appears after immunization. These volunteers should be seen in the clinic at the time of maximal symptoms, if possible, and will be followed up clinically until resolution of symptoms.

An adverse event is any undesired, noxious or pathological change in a patient or volunteer as indicated by physical signs, symptoms, and/or laboratory changes that occurs following administration of one of the vaccines, whether or not considered vaccine related. This definition includes intercurrent illnesses or injuries, and exacerbation of pre-existing conditions.

Local adverse events
Pain in the muscle injected will be graded by the volunteer according to the criteria in the specific appendix as mild (1) moderate (2), severe (3) or serious (4) and recorded in the appropriate case record form or once the volunteer has left clinic, in the diary card. Redness and or induration will be recorded as the maximum diameter and expressed as a proportion of the arm circumference and graded on this criteria and the presence of symptoms according to the specific appendix. Arm circumference will be measured at screening, at the point one third of the way down from the acromio-clavicular joint towards the elbow joint in the injected arm. Investigating personnel will record the maximum diameter, as well as the presence of any itching or other discomfort and any medication taken for relief of symptoms. Blistering (vesiculation) or ulceration will be graded according to size, depth, time to healing and character of blisters (blood-filled).

The sole presence of an induration following intramuscular injection considered grade 3 will not be sufficient to stop the trial (see table). Clinical staff will complete the case record form following each immunisation. In the event that there are two observations on the same day, those confirmed at a visit will take precedence over diary card observations in the analysis. Study personnel should verify all grade 3 or 4 events.

Systemic adverse events
Temperature will be measured by the axillary route prior to immunisation and 30 minutes after the immunization by study personnel, and graded according to the table in ***appendix xiv***. Volunteers will be given thermometers to record their temperature in the diary card on the evening of immunisation, and daily thereafter for 7 days, and if still raised, they will be advised to continue to monitor their temperature daily until it returns to normal. The temperature observed by the clinical team will be recorded in the case record forms for visits. In case of grade 3 and 4 fever, malaise, chills/rogors and headache, and where indicated, additional tests to exclude other common causes of fever like malaria will be performed.

Chills, headache, nausea, vomiting, malaise and myalgia will be graded by the volunteers according to ***appendix xiv***, recorded in the diary card on the evening following immunization and daily for 7 days, or until resolution of symptoms whichever is longer. On days where a visit coincides with the diary card, the clinical staff will collect the information directly and record it on the case record form, and this will take precedence over diary card entries in the analysis. Study personnel will verify all grade 3 or 4 events.

Other adverse events
These will be recorded as reported following an open question to volunteers, with the dates of commencement and resolution, and any medication required. They will be graded according to the general principles outlined in ***appendix xiv***. Social harm will also be recorded as an adverse event, graded according to the general guidelines (which include denial of health and life insurance, employment, immigration and marriage relationships). All grade 3 or 4 events will be verified by study personnel.

Routine laboratory parameters for safety assessments
The following safety assessments will be undertaken at appropriate laboratories in MUHAS according to standard operating procedures.

Haematology
Haemoglobin; white blood cell count, total and differential counts, specifically granulocytes and lymphocytes; platelet count and red blood cell counts.

Clinical chemistry
Liver function tests (ALT, bilirubin total and direct), and serum creatinine and blood glucose.

Immunology
Determination of CD4 counts and percentage
Syphilis (VDRL, and when necessary TPHA)
Screening for HBsAg

Other tests
ECG will be done at MVA vaccination and two weeks after the MVA vaccination. It will also be done at anytime whenever a volunteer comes with symptoms and signs suggestive of peri-myocardtitis.

Troponin test
Serum for a later troponin test will be collected

The following assessment will be undertaken on a specimen of urine, conducted by a member of the study staff according to trial specific standardised operating procedures:

Urinalysis
Dipstick conducted by clinical staff and recorded normal/abnormal. If abnormal a mid-stream urine specimen will be collected in order to grade the event according to ***appendix xiv***.

All grade 3 or 4 adverse events or serious events and/or those that necessitate a physician's visit or are treated with a prescribed medication will be evaluated. The volunteer will be followed carefully until the condition is resolved and/or the cause is identified. Any medication or other therapeutic measure taken to relieve symptoms of the medical problem must be recorded on the appropriate case report form page(s) in addition to the outcome of the adverse event. Where a diagnosis is possible, it is preferable to report this rather than a series of terms relating to the diagnosis. When reporting a syndrome, the associated signs and symptoms will be parenthetically indicated following the syndrome rather than as separate event.

IMMUNOGENICITY ASSESSMENTS

Antibody responses
HIV serology for seropositivity will be performed on samples collected at screening and at week 60 and week 76.
Antibodies to HIV proteins covering the vaccines will be assessed using ELISA. Following completion of the study, neutralizing antibodies to HIV subtypes A, C and D will be performed. Serum samples at different time points will be collected as per specimen schedule and will be stored until the time for the assay. In vivo antibody neutralization will be used for testing. At this point in time, it is not known which time point sample is going to induce broadly neutralizing antibodies or not. Therefore, testing will be performed on the last serum sample collected 2 weeks post vaccination and other earlier time point samples should the last sample testing show any interesting results

The specific assessment of HIV-1 specific antibody responses will thus include:
- Serum binding antibody determination by ELISA and Western blot assay
- Serum neutralization antibody assay including cross clade reactivity (against HIV-1 subtypes A, C and D which are prevalent in Tanzania)

Cellular responses
The assessment of primary cellular responses will be achieved by determining the following HIV-1 specific cellular immune responses:
- CD8 and CD4 T helper cell responses
  - IFN-γ ELISPOT and Interleukin-2 assay (on stored Peripheral Blood Mononuclear Cells (PBMC's)), including determination of cross clade responses, using pools of overlapping peptides representing env, gag and pol proteins
  - Intracellular cytokine staining (ICS) for IFN-γ and Interleukin-2 (IL-2) (ref 27) using the same peptide pools as in the ELISPOT and testing both for CD8 and CD4 T cell responses
- CD4 T helper cell responses
  - Lymphocyte proliferation assay (LPA) to the immunizing antigens and mitogens on fresh PBMC's
    - Standard assay: measurement of proliferation by uptake of ^{3}H-thymidine
    - New assay: whole blood flow-cytometric assay (FASCIA=Flow-cytometric Assay of Specific Cell-mediated Immune response in Activated whole blood) developed at SMI. Both CD4 and CD8 T cell responses can be demonstrated by this method.

- CD8 T cell response
  - Cytotoxic T lymphocytes (CTL) by chromium release assay

Interferon-gamma Enzyme-linked immunospot (ELISPOT) will be used as a standard assay to monitor HIV cellular immune response in vaccinated individuals and will be performed on fresh PBMC's. These procedures will be validated prior to the trial. Individuals with <50 SFC/million PBMCs will be regarded as non-responders. If the average value of sample wells is >4 times the

background (medium only) and >55 SFC/million PBMCs the individuals will be regarded as responders.
A new whole blood flow-cytometric assay (FASCIA=Flow-cytometric Assay of Specific Cell-mediated Immune Response in Activated whole blood) developed at SMI will also be performed later on stored PBMCs. Both CD4 and CD8 T cell responses can be demonstrated by this method. Quality control and exchange of specimens with international laboratories will be performed.

Storage of Cellpellets for HLA testing
Lymphocyte cellpellets will be stored at MUHAS department of Microbiology/Immunology for later HLA typing that will be performed by molecular methods at a laboratory to be identified later.

OTHER ASSESSMENTS

HIV testing
Samples will be tested at MUHAS, department of Microbiology/Immunology using HIV antigen/antibody ELISA, and discordant results will be confirmed by a Western blot Inno-Lia immunoblot according to standard operating procedures.

Antibodies against Vaccinia
Serum samples will be stored at MUHAS, department of Microbiology/Immunology for later testing.

Following adverse event
Other assessments may be performed as clinically indicated due to an adverse event.

CONCOMITANT MEDICATION
Volunteers will be asked about medication taken at each visit up to and including week 18, and this will be recorded in the case record form. Thereafter, they will be asked about medication only in relation to adverse events reported. It is expected that the name of the drug, indication for use, dose, frequency, start and stop dates will be available either from the volunteer or from the prescribing physician. For medication available over the counter or other sources, the maximum information available on questioning the volunteer will be recorded.

ADVERSE EVENTS
Volunteers will be provided with a diary card, disposable thermometers and ruler at the immunizations.

Definitions
An adverse event is any adverse experience occurring during the course of the study including the screening period. Criteria for grading clinical and laboratory events are listed in ***appendix xiv***. The severity of events reported on the adverse event form will be determined by the investigator, based on the Toxicity Grading Scale or if the event is not listed the event will be graded following these guidelines:

- Mild (Grade 1): Transient or mild discomfort. No limitation in normal daily activity.
- Moderate (Grade 2): Some limitation in normal daily activity.
- Severe (Grade 3): Unable to perform normal daily activity.
- Serious (Grade 4): Life threatening
- Death (Grade 5): Death

A severe adverse event is one **graded 3 or 4** by criteria in ***appendix xiv***. Some, but not all grade 3 and 4 adverse events will be "serious" by ICH GCP criteria below.

Serious Adverse Events (SAEs)
An adverse event is considered to be a "serious adverse event" by ICH Good Clinical Practice (ICH GCP) criteria if it results in the following:
1. Death,
2. A threat to life,
3. Requires in-patient hospitalization or prolongs existing hospitalisation (hospitalisation for elective treatment of a pre-existing condition is not included),
4. Results in persistent or significant disability or incapacity,
5. Is a congenital anomaly (i.e. the outcome of pregnancy involving a volunteer),
6. Is any other important medical condition*.

*Examples of conditions regarded as "any other important medical condition" include allergic bronchospasm requiring intensive emergency treatment, seizures or blood dyscrasias which did not result in hospitalisation or development of drug dependency.

Relationship of adverse events to study product

This can be classified as:

None: Adverse events felt to be due to extraneous causes that neither follow a known pattern of response nor a reasonable temporal relationship to study product.

Remotely related: Adverse events that are unlikely to be related to product but which follow a reasonable temporal relationship, such that this cannot be completely excluded or events that could be associated with product but which are unrelated in time.

Possibly related: Adverse events that may be due to extraneous causes but which follow a known pattern of response and/or a reasonable temporal relationship to study product.

Probable: Adverse events that cannot be explained by extraneous causes; which follow a known pattern of response and/or a reasonable temporal relationship; which disappear or decrease on cessation of study product and reappear on re-challenge.

Reporting adverse events
Adverse events should be recorded on the appropriate case record form (CRF 8-III). Any grade 3 or 4 adverse event (see ***appendix xiv***) or any event resulting in discontinuation of the vaccination schedule should be reported within 2 working days to the DSMB for review and appropriate recommendations. The proposal as to whether continue or discontinue the study will be submitted to the monitor, TCC, NIMR and TFDA for further review and action

Serious adverse events should be reported to the monitor the same working day that the Clinical Investigator becomes aware of the event fulfilling the above criteria. This can be done by telephone, e-mail or fax. The minimum criteria required in reporting a SAE are the volunteer identifiers (trial number/ date of birth/initials), reporting source (name of Investigator), and why the adverse event is identifiable as serious. The adverse event reporting telephone/ fax number for MUHAS is 255 22 2153027 (HIVIS office). The address for reporting to the monitor will be determined by the study monitor.

The monitor will confirm that the event is potentially reportable and arrange for urgent review of the case by the TCC within 2 working days. Should the case be considered reportable by this group, a report will be prepared by the clinical investigator or his deputy and filed with the TFDA and the DSMB. The Principal Clinical Investigator is responsible for notifying the Local Research Ethics Committee.

Examples of SAEs that do not require expedited reporting include:
1. Hospitalisation for scheduled surgery unrelated to vaccine, other than temporally (within 30 days).
2. Orthopaedic or traumatic injuries requiring hospitalization.
3. Hospitalisation planned for pre-existing conditions not due to an aggravation in the condition.
4. SAEs occurring more than 30 days after vaccination or having appeared before vaccination without any aggravation after vaccination

DISCONTINUATION PROCEDURES (including withdrawal and stopping participation in the study)

Volunteers may withdraw at any time if they wish to do so, for any reason. The date of withdrawal and reason for doing so should be recorded in the appropriate case record form. The clinical investigator may decide that it is not in the best interests of the volunteer to proceed to the next immunisation following an adverse event (see below). The discontinuation and reason should be recorded in the appropriate immunisation and reportable adverse event case record forms, which should be forwarded to DSMB within the next 2 working days. Trial visits should carry on at least until resolution or stabilisation of the event, but ideally up to the last visit in the schedule, provided the volunteer is willing. The frequency of visits and laboratory investigations may be reduced on consultation with the Principal Investigator or Trial Management Group

Criteria for withdrawal of volunteers from injection schedule
Under certain circumstances, a volunteer will be terminated from participating in further injections. These events include:
1. HIV infection

2. Pregnancy
3. Grade 3 or 4 systemic events classified as probably or definitely associated with immunization (with the exception of grade 2 nausea, headache or myalgia).
4. Type 1-hypersensitivity associated with immunization.
5. Serious intercurrent illness that is not expected to resolve prior to next scheduled immunization.
6. Need for systemic glucocorticoids or other immunomodulators than NSAIDs for any reason.
7. Repeated failure to comply with protocol requirements.
8. The Sponsor, vaccine provider or principal investigator decides to stop or cancel the study.
9. The NIMR and or MUHAS ethical committee decides that the study be stopped

Trial volunteers who are discontinued from additional study injections will continue to be followed according to the schedule to further evaluate safety and monitor adverse events. They will also continue to be covered by the medical insurance till the end of the study period.

Criteria for stopping the study

1. The Principal investigator will closely monitor and analyse study data as it becomes available and will make determinations regarding the presence and severity of the adverse events
2. Any grade 3 systemic event assessed by DSMB as possibly, probably or definitely related to the vaccination (following exclusion of other unrelated common causes) or if one volunteer experiences grade 4 or 5 (death) SAE assessed as possibly, related to the vaccination.

The study injections and enrolments would resume only after review of the adverse events by the DSMB and a decision by the TCC including the Chairman resulted in a recommendation to permit further study injections and study enrolments. Safety data and changes in study status will be submitted to the ethics committee.

CLINICAL MANAGEMENT

The clinical trial team who will assess and treat the event as appropriate will manage events.

Management of HIV issues during and following the trial

Individuals who test HIV-positive during the screening process will be referred for HIV care, treatment and support at the HIV/AIDS clinic MNH. At this clinic the following services will be provided; sustained counseling about HIV/AIDS, counselling on diet, management of symptoms and opportunistic infections as well as provision of antiretroviral drugs if indicated

HIV testing

Only volunteers with a non-reactive antigen/antibody HIV ELISA result will be enrolled. A risk assessment will be undertaken prior to every immunisation and if risk status has changed then immunization may have to be delayed until the HIV status of the volunteer is clarified.

It is possible that volunteers may develop antibodies and test ‘positive’ in routine commercial HIV ELISA assays subsequent to immunisation. The laboratories at MUHAS, department of

Microbiology/Immunology, will conduct molecular tests (HIV-1 DNA PCR) to distinguish between infection and a post-immunisation response required either for clinical management, or at the request of a volunteer.

Weeks 40, 60, 76 and 104 specimens will be tested in real-time by antigen antibody ELISA and DNA PCR if found to be ELISA positive, so that volunteers can be informed of the result before the end of the trial and a plan to recall and retest them made should this be necessary. In the event of ongoing post-immunisation positive ELISA, the volunteers will be invited to re-attend specified centre annually until such time as this response has disappeared, and provided with an explanatory identity card in the interim.

Verification of HIV status of volunteers
If certification is required at the request of the recruited volunteer, this can be provided by the trial Principal investigator after testing at the trial laboratory. Results will always be given at post-test counseling session by a member of the study team, unless the volunteer requests otherwise. If a specimen from a volunteer suggests that the volunteer is HIV infected, a second specimen will be collected, retested and the results confirmed. If a volunteer for any reason needs to establish his or her HIV status extended tests will be offered as described above.

Management of volunteers who acquire HIV infection during the study period
In the unexpected circumstances that a volunteer in the trial acquires HIV infection they will be managed in the following way:

Referral for clinical care
Volunteers will be referred initially to a study HIV specialist physician at MUHAS/MNH for a full discussion of the clinical aspects of HIV infection and planning for further care. Further investigations will be undertaken as necessary. At this clinic the following services will be provided, sustained counseling about HIV/AIDS, counselling on diet, management of symptoms and opportunistic infections as well as provision of antiretroviral drugs if indicated. Efforts will be done in collaboration with the Ministry of Health that such individuals are assured of life-long follow up at MNH/MUHAS in terms of their immunological and clinical fate

Referral for counseling
A referral to a counselor at the HIV clinic at MNH will be arranged by the specialist physician. The counseling process will assist the volunteer in the following issues:

a. Psychological and social implications of HIV infection
b. Who to inform and what to say
c. Implications for sexual partners
d. Facilitation of risk reduction plan
e. Avoidance of risk to others in the future
f. Information on available medical treatment

Informing the other involved heath care personnel
The volunteer will be encouraged to do this, but the decision will remain at the discretion of the individual.

Immunological follow-up

Follow-up of HIV infected individuals who have received study vaccine products will be determined by the Trial Management Group for the duration of the study and after the study period they will be referred to the existing treatment center. The intensity of assessments will be dependent on clinical progression, including changes in surrogate markers such as CD4 count.

If a volunteer is found to be HIV-infected during the trial, he/she will be excluded from further immunizations in the trial.

SOCIAL DISCRIMINATION AS A RESULT OF A POST-VACCINE RESPONSE

The aim is to minimize the possibility of social discrimination in volunteers who develop a positive HIV-ELISA test by providing HIV testing by PCR and certification for volunteers as required. In addition, an identification card stating that the individual has participated in a vaccine trial, with a contact number in case of medical emergency, will be provided. In the unlikely event that a volunteer suffers social discrimination as a result of a post-vaccination response, the clinical investigators will assist the volunteer.

TRIAL DATA MANAGEMENT

Data management at the Clinical Centre

Data will be recorded directly onto the case report forms, which will provide the majority of source data for the trial. There will be some additional source data in the clinical notes, such as medical history related to eligibility, results of laboratory analyses, dates of visits including immunisation, and details of clinical management (description of adverse events and concomitant medication). The Clinical co-ordinator or his/her designate will cross-check the completeness of the CRF's at the clinic. The original copy will be kept at the clinic, and one photocopy of the completed case report form will be forwarded to the trial external monitor at the Karolinska Institute, Sweden for inspection by the monitor and entry into the database. Another data base will be maintained at the HIVIS office, Makuti, MNH. Any changes to the CRFs should be signed and dated, including changes made before a copy is forwarded to the trial monitor and data entry. Study related documents including clinical notes and original laboratory results should be kept in a secure location at MUHAS after completion of the trial.

Data management in the Microbiology/Immunology laboratories

Standardised operating procedures will be followed in all laboratories to ensure the quality of data. Data will be stored in both hard copies and electronically in an agreed format and data files transferred to trial coordinating centre for the main analysis. As for clinical data all results from the laboratory will be reviewed by the laboratory investigator for completeness before forwarding them to the trial coordination centre database. He/She will review both the electronic forms and hard copies and approve them by signing the copies. The original copy will be stored at the laboratory and one copy sent to the trial coordination center for data entry.

Data management at the trial coordination centre (Karolinska Institute)

All CRFs will be reviewed for completeness and inconsistencies by the data manager according to standardised operating procedures. Any enquires will be communicated with the clinical centre and will be passed for review by the trial physician if required. The data manager will approve the CRFs for data entry into the database. Data will be coded and doubly entered into a computerized database. Consistency checks and range checks will be performed at the data entry level.

After approval and entry into the database, the copy including documentation detailing all the queries and changes will be stored in a study specific binder at the trial coordination centre. The documents in the binder will be organized according to study number. The original case report forms will be stored together with the individual source data at the clinical centre.

Archiving

Study related documents including clinical notes, completed CRFs and original laboratory results, hard copies and electronic copies will be kept in a secure location at MUHAS.

STUDY MONITORING
A trial monitor will be appointed by the sponsors of the study, MUHAS and SMI; he/she will be responsible to the Principal Investigators of the Project and the Trial Coordinating Committee to ensure that the trial is conducted according to ICH GCP guidelines including monitoring vaccine data consistency, vaccine accountability, dispatch and arrival of immunological specimens.

Preparation of reports to assist the monitoring
The monitor will visit the clinical centre to validate trial data against the clinical records. The following data should be verifiable from source documents: (Initials, study registration number, sex, birth date, address)

i. Documentation of any existing conditions or past conditions relevant to eligibility
ii. A signed consent
iii. Dates of visits including dates of immunizations
iv. Source verification of all data entered into the database
v. Grade 3 or 4 adverse events and any events leading to discontinuation of the immunisation schedule
vi. Concomitant prescribed medication

Vaccine stocks will also be monitored at visits to the clinical centre/pharmacy.

DATA OWNERSHIP
The data generated in this study will be the property of the HIVIS Investigators and will be held on their behalf by MUHAS until completion of the study or until it is stopped. Thereafter the data will be passed over to MUHAS for archival purposes according to accepted regulation for a maximum of 5 years. If required at an earlier date for the purposes of regulatory submissions, a request should be made to the Trial Coordinating Committee.

TRIAL COMMITTEES
Trial Coordinating Committee (TCC)
The supervision of the trial will be a responsibility of the Trial Coordinating Committee (TCC). The committee will have an independent chair and a secretary. The members will include the 2 Principal Investigators, study director, clinical co-ordinator, laboratory co-ordinator, study nurse and data manager. The TCC will be meeting at least once a week during the trial period. The TCC will prepare a monthly report for presentation at the HIVIS governing board meeting. This committee will be responsible for final decisions about grade of adverse events and relationship to study vaccine. Notes of meetings will be kept, and the Committee will report to the HIVIS Project Governing Board. The trial may be terminated by this Committee for any reason, including on the recommendation of the DSMB.

Trial Management Group (TMG)
This group will oversee the day-to-day running of the trial and the members will be primarily the clinical and data management teams. The members will be the clinical co-ordinator, study nurse and data manager. The Microbiologists will join if there are relevant items on the agenda. Notes will be taken and will form the basis of the progress report to the Trial Coordinating Committee.

HIVIS Governing Board
The HIVIS Governing Board will review the notes from the TCC meetings regarding progress of the trial, and will be meeting once a month.

Data and Safety Monitoring Board (DSMB)
An independent Data and Safety Monitoring Board will be appointed by the sponsor of the study, MUHAS and SMI. The role of the DSMB will include;

1. To review the research design and the plans for data and safety monitoring
2. To receive and review at intervals the progress reports from the investigators
3. To monitor safety data
4. To monitor and evaluate trial progress, including
    a. Periodic assessment of data quality and timelines
    b. Performance of the study sites
5. To recommend to the sponsor, local IRBs and investigator concerning continuation or to stop the study.

The DSMB will meet whenever a safety report has been submitted and make a recommendation to the TCC within 7 days as to the future conduct of the trial.

They will also meet on one occasion to review the safety data, after half the volunteers have received the first immunization in order to make recommendations to the sponsors of the study.

Community Advisory Board
There will also be a Community Advisory Board drawing members mainly from the Police Force. There may also be members from the media as well as legal representation.
In addition, the wider Tanzanian community has since the first high profile National HIV vaccine Strategy Plan Workshop held in Bagamoyo in 2001 been informed through the mass media on the need and on plans for HIV vaccine trials in Tanzania. The public will continue to be informed through press releases and through other channels on all the stages of the HIVIS trial.

INDICATIONS FOR ADDITIONAL REVIEW
There will be an additional meeting if 3 or more volunteers experience an unexplained, unexpected grade 3 or 4 clinical or laboratory event (confirmed on attendance or repeat testing) not resolved within 72 hours and considered probably or possible and likely to be related to vaccine product.

STATISTICAL CONSIDERATIONS
By the end of this study 60 volunteers will have been exposed to DNA or placebo as well as MVA or saline placebo and described in the quantitative and qualitative immunogenicity and safety evaluation.

Analysis
All safety end-points will be graded by the Clinical Investigator and reviewed by the Trial Management Group. Any queries about grade and relationship to study product that cannot be resolved will be referred to the Trial Coordinating Committee for a final decision.

The primary end-points are:

1. The safety of immunization with the seven DNA plasmids carrying HIV-1 genes as assessed by clinical signs, and standard biochemical/haematological laboratory tests where any worsening of the severity grade will be considered for causality by the vaccine

2. A qualitative and quantitative evaluation of the magnitude and quality of humoral (neutralizing and binding antibodies, by serology) as well as cellular (cytotoxic T-cell, IFN-gamma by ELISPOT, T-cell proliferation (Fascia) and intracellular cytokines by flow cytometry) immune responses as elicited by the different schedules of immunization

The alternative routes of immunization (im and id with the Bioject device) will be evaluated for adverse events by assessing local (pain, cutaneous including induration), general (fever, chills, headache, nausea, vomiting, malaise, myalgia) and other unsolicited adverse events within 7 days. Any grade 3 or 4 event will be taken as an indication that the arm is less tolerable

The secondary endpoints are:

1. A qualitative and quantitative assessment on the presence of necessary infrastructure and human capacity to conduct HIV-related vaccine studies at MUHAS.

Statistical analysis
The final analysis will be conducted once all study participants have received all their immunizations and completed a 24 weeks follow-up period, or have permanently withdrawn from the study. Safety analyses will further be updated in the event that there is a 52 weeks follow up. Analyses will be based on the principle of intention-to-treat.

Volunteer characteristics
Baseline characteristics will be summarized by study arms, using appropriate summary statistics. Study participant characteristics to be summarized will include demographic variables and baseline values relevant to safety and immunogenicity.

Safety data
The number and percentage of subjects experiencing any adverse events (AE), grade 3 or 4 adverse events, serious adverse events (SAE), and the number of events reported, will be summarized by severity and reported relationship to study treatment for each treatment group, both for all adverse events and by type of adverse event. Calculation of incidence rates of adverse events will include each subject only once, either according to worst severity or first reported event.

Rates of the various adverse events will be compared between the study arms using Fisher's exact test. Rates will be summarized using exact binomial confidence intervals

Safety haematological and biochemical laboratory parameters will be summarized at each study visit according to study arm. Comparisons will be done using 2-sample t-tests or non-parametric equivalents as appropriate to the data

Immunogenicity Data
Immunogenicity endpoints will be compared formally at week 0, 10 and 12, according to study dosing group. Binary endpoints will be compared using Fischer's exact test. Continuous endpoints will be compared using 2-sample t-tests or non-parametric equivalents as appropriate to the data.

Immunogenicity of the candidate vaccine will be determined based on comparisons between study treatment arms. Associations between immunogenic endpoints will be assessed using multivariate regression models.

ETHICAL CONSIDERATIONS

Full medical confidentiality will be preserved. The study will be conducted according to ICH GCP guidelines and the Declaration of Helsinki (version 2002), and it is the responsibility of the Clinical Investigators to ensure adherence. Before the study starts all personnel involved in the clinical and laboratory trial units will be trained for Good Clinical Practice (GCP) and/or Good Laboratory Practice (GLP) respectively. Study personnel will also be trained on standard operating procedures (SOPs) that are related to the protocol and will follow the GCP and GLP guidelines.

This protocol has been reviewed by WHO VAC advisory board. It has also been submitted to the national ethical committees in Tanzania, ie, National Institute of Medical Research (NIMR) and after receiving approvals from NIMR will then be submitted to the institutional ethical committee at Muhimbili University College of Health Sciences (MUHAS) and subsequently to the appropriate ethics committee in Sweden. The DNA and MVA vaccines which will be donated by the Karolinska Institute in Sweden have been approved by the Swedish Medical Products Agency and are currently used in the ongoing clinical trials in Sewden. The US Food and Drug Authority has previously approved the MVA which is in ongoing clinical trials in the USA. Approval of these immunogens will also be undertaken independently by the Tanzania Food and Drug Authority (TFDA).

The Principal and Clinical Investigators are also responsible for informing the ethics committees of any SAEs as required, and submitting annual reports as required. No study materials will be obtained from study volunteers before approval from the relevant bodies. The Karolinska institute in Sweden will be responsible for providing the Investigator’s Brochure (IB) for DNA as well as the Investigator’s brochure for MVA.

The TCC will be responsible for preparing the randomisation list, all aspects of data management including monitoring of the clinical sites, and the analysis. Staff will also be responsible for coordinating the response to any SAEs that arise during the course of the trial and reporting these if indicated to the regulatory authorities.

INDEMNITY

Insurance for investigators

The Principal Investigator will ensure that relevant clinical and laboratory staff engaged in the study are covered for possible legal actions against the investigators for harm through indemnity insurance schemes which will be paid for by the project during the study period.

Health care Insurance for trial volunteers

All trial volunteers will be provided with health insurance cover for injury or death related to the trial through insurance schemes.

Vecura company in Sweden will have an insurance that covers any defect in the production of the immunogen.

PROTOCOL AMENDMENTS

The TCC will be responsible for preparing protocol amendments needed by the HIVIS governing board. Amendments to the protocol will be made only after consultation and agreement between sponsors and Principal investigators. The only exception is where the study director considers that a volunteer’s safety is compromised without immediate action. All amendments that have an impact on volunteer risk or the study objectives, or require revision of the informed consent document, must receive approval from the relevant ethical committees prior to their implementation.

PUBLICATION

It is intended that the results of this study will be published in an appropriate peer-reviewed journal, with the aim of submitting a paper for publication within 6 months of the study’s completion.

The HIVIS Trial Coordinating Committee and Bioject Inc will have 30 days to comment on any manuscript. No other publications, whether in writing or verbally, will be made before the definitive manuscript has been agreed and accepted for publication, without the prior approval of this committee. A final report of the study will be prepared by the Investigators and circulated to the HIVIS governing board for comments.
The presenting author for the first conference presentation of the main trial results will be one of the Tanzanian principal investigators or their deputy.

STUDY PERSONNEL & RESPONSIBILITIES

MUHAS/MNH

Clinical
Kisali Pallangyo, (MD, MMed). Co-Principal Investigator
Responsible for overall conduct of the study in collaboration with the other co-PI
Handles scientific, Administrative, and Financial matters pertaining to the study. Specific responsibility in ensuring that the Clinical Research Organization is in place and functions properly.

Muhammad Bakari, (MD, MMed, PhD). Study Director
Responsible for day to day study activities, overseer of protocol development and financial matters. He is also the Project Co-oridnator for the EDCTP-funded TaMoVac Project

Joel Francis, (MD, MSc). Study Clinical co-ordinator
Principal co-ordinator of study clinical activities. Patient recruitment, clinical evaluation, and follow up. Prepares responses to Monitoring reports. Ensures integrity of CRF's, and conduct of trial in accordance with SOP's. Over-seer of Clinical staff.

Eric Aris, (MD, MMed)
Preparation of study subjects and training of staff, protocol development, conduct of the trial

Mohamed Janabi, (MD, MSc, PhD)
Preparation of study subjects, protocol development, conduct of the trial

Ferdinand Mugusi, (MD, MMed)
Training of staff and smooth running of the clinical trial unit

Joachim Mwami, (BA, MA, PhD)
Preparation of study subjects, social issues

Edith Mrosso, (BScN, MPhil)
Preparation of study subjects, social issues

Deus Buma (BPharm), Study Pharmacist
Responsible for vaccine procurement, storage, preparation, dispensing and disposal.

Lughano Kabadi (BPharm), Deputy Study Pharmacist
Assists the study Phramacist

Laboratory
Fred Mhalu, (MBChB, DMed). Co-Principal Investigator
Responsible for overall conduct of the study in collaboration with the other co-PI
Scientific and Administrative issues pertaining to the study. Specific responsibility in ensuring that laboratory aspects of the study function properly

Eligius Lyamuya, (MD, MMed, PhD)

Will oversee proper specimen collection, storage and ultimate determination of prevalent HIV-1 strains. He is the Laboratory Co-ordinator, responsible for day to day co-ordination of all laboratory activities.

Willy Urassa, (MD, MMed, MSc, PhD)
Laboratory Investigator
Responsible for laboratory enhancement and immunological aspects of the study

Said Aboud, (MD, MPhil, MMed)
Deputy Laboratory Coordinator.Also responsible for safety laboratory tests and immunological tests

Judica Mbwana, (ADMLS, MSc., Ph.D)
Laboratory Technical Manager
Overall responsible for technical Coordination and supervision

Data Management
Candida Moshiro, Ph.D., Data manager
Day to day data management. Production of reports

Data entrants (2)
Entry of data, doubly

Support staff
Senior Study Nurse(s)
Administration of vaccine to volunteers. Follow up for immediate and later ADR
Driver,
Administrator,
Messenger/Office assistant

COLLABORATORS
SWEDEN (Karolinska Institute)
Eric Sandstrom
Monitor of the study on behalf of the Karolinska Institute (KI) in Stockholm,Sweden

Gunnel Biberfeld
Immunological aspects, establishment of ELISPOT assays at MUHAS, training of lab personnel

Charlotta Nilsson
Immunological aspects, training of laboratory personnel, Quality control & assessment of lab activities.
Laboratory Monitor of the study on behalf of the Swedish Institute for Infectious Disease Control, Stockholm, Sweden

Britta Wahren
Oversees production of DNA and delivery of DNA and MVA

Bo Hejdeman

Protocol development, liase for data on Swedish trial, Trial issues in Tanzania

GERMANY (University of Munich)
<u>Michael Hoelscher</u>
Strain characterization

SOUTH AFRICA (University of Cape Town)
<u>Carolyn Williamson</u>
Strain characterization

REFERENCES

1. UNAIDS/WHO-2004. AIDS epidemic update December 2004. Geneva, Switzreland. Available at http://www.unaids.org/wad2004/EPI_1204_pdf_en/Epiupdate04_en.pdf

2. Ministry of Health, National AIDS Control Program. Medium Term Plan III, 1998-2002.

3. Bakari M, Urassa W, Pallangyo K, Swai A, Mhalu F, Biberfeld G, Sandström E. The Natural Course of Disease Following HIV-1 Infection in Dar es Salaam, Tanzania; a Study among Hotel Workers Relating Clinical Events to CD4+ T lymphocyte counts. Scand J Infect Dis. 2004; 36(6-7):466-73.

4. Bakari M, Lyamuya E, Mugusi F, Aris E, Chale S, Magao P, Josiah R, Janabi M, Swai A, Pallangyo N, Sandstrom E, Mhalu F, Biberfeld G, Pallangyo K. The prevalence and incidence of HIV-1 infection and syphilis in a cohort of police officers in Dar es salaam, Tanzania. A potential population for HIV vaccine trials. AIDS. 2000; 14: 1-8

5. Kwesigabo G, Killewo J, Godoy K, et al. Decline in the Prevalence of HIV-1 Infection in Young Women in the Kagera Region of Tanzania. J Acquir Immune Defic Syndr Human Retrovirol. 1998; 17:262-268

6. Matee MI, L.E., Mbena EC, Magessa PM, Sufi J, Marwa GJ, Mwasulama OJ, Mbwana J., Prevalence of transfusion-associated viral infections and syphilis among blood donors in Muhimbili Medical Centre, Dar Es Salaam, Tanzania. East Afr Med J. 1999; 76(3): 167-71

7. Ministry of Health, National AIDS Control Program. Medium Term Plan III, 1998-2002. 2002

8. Esparza J, Osmanov S, Pattou-Markovick C, et al. Past, present and future of HIV vaccine trials in developing countries. Vaccine. 2002 May 6;20(15):1897-8

9. Mugwera RD, Kaleebu P, Mugyenyi P, et al. First trial of the HIV-1 vaccine in Africa: Ugandan experience. Br Med J. 2002; 324: 226-2

10. Cao H, Kaleebu P, Hom D, et al. Immunogenicity of a recombinant human immunodeficiency virus (HIV)-canarypox vaccine in HIV-seronegative Ugandan volunteers: results of the HIV Network for Prevention Trials 007 Vaccine Study. J Infect Dis. 2003 Mar 15;187(6):887-95

11. Jaoko, W, Omosa G, Bhatt K, et al. Safety and immunogenicity of DNA and MVA vaccines in phase I HIV-1 vaccine trials in Nairobi, Kenya. AIDS Vaccine 2004 Conference, Laussane, Switzerland. August 30th – Sept 1st. Oral abstract No 56

12. Adis International Ltd. HIV gp120 vaccine - VaxGen: AIDSVAX, AIDSVAX B/B, AIDSVAX B/E, HIV gp120 vaccine - Genentech, HIV gp120 vaccine AIDSVAX - VaxGen, HIV vaccine AIDSVAX - VaxGen. Drugs R D. 2003;4(4):249-53

13. Robinson HL. New hope for an AIDS vaccine. Nature reviews 2002;2:239-250.

14. Rowland-Jones S, Sutton J, Ariyoshi K, et al. HIV-specific cytotoxic T-cells in HIV-exposed but uninfected Gambian women. Nature Med 1997. 1:59-64.

15. Mazzoli S, Trabattoni D, Lo Caputo S, et al. HIV-1 specific mucosal and cellular immunity in HIV-seronegative partners of HIV-seropositive individuals. Nature Med 1997. 3:1250-1257.

16. Lyamuya E, Olausson-Hansson E, Albert J, Mhalu F, Biberfeld G. Evaluation of a prototype Amplicor PCR assay for detection of human immunodeficiency virus type 1 DNA in blood samples from Tanzanian adults infected with HIV-1 subtypes A, C and D. J Clin Virol. 2000; 17(1):57-6

17. Hoelscher M, K.B., Maboko L, Mhalu F, von Sonnenburg F, Birx DL, McCutchan FE; UNAIDS Network for HIV Isolation and Characterization. High proportion of unrelated HIV-1 intersubtype recombinants in the Mbeya region of southwest Tanzania. AIDS. 2001; 15(12):1461-70

18. Kiwelu IE, R.B., Chaplin B, Sam N, Nkya WM, Shao J, Kapiga S, Essex M. HIV type 1 subtypes among bar and hotel workers in Moshi, Tanzania. AIDS Res Hum Retroviruses. 2003; 19(1):57-64

19. Renjifo B, C.B., Mwakagile D, Shah P, Vannberg F, Msamanga G, Hunter D, Fawzi W, Essex M. Epidemic expansion of HIV type 1 subtype C and recombinant genotypes in Tanzania. AIDS Res Hum Retroviruses. 1998; 14(7):635-8

20. Koulinska IN, N.u.T., Mwakagile D, Msamanga G, Kagoma C, Fawzi W, Essex M, Renjifo B. A new human immunodeficiency virus type 1 circulating recombinant form from Tanzania. AIDS Res Hum Retroviruses. 2001; 17(5):423-31

21. Ljungberg, K., Rollman, E., Eriksson, L., Hinkula, J. & Wahren, B.: Enhanced immune responses after DNA vaccination with combined envelope genes from different HIV-1 subtypes. Virology. 302; 44-57, 2002

22. Rollman E, Zuber B, Kjerrström A, Arteaga J, Hinkula J, Liu M, Wahren B, Ljungberg K. Multisubtype gp160 DNA immunization induces broadly neutralizing anti-HIV antibodies. Gene Therapy. 2004; 11: 1146-1154

23. Bråve A, Ljungberg K, Boberg A, Rollman E, Johansson S, Lundgren B, Blomberg P, Hinkula J, Wahren B. Needle free intradermal delivery of a multigene/multi-subtype vaccine with recombinant GM-CSF induces potent cellular and humoral immune responses to HIV-1. Manuscript 2005.

24. Mäkitalo B, Lundholm P, Hinkula J et al. Enhanced cellular immunity and systemic control of SHIV infection by combined parenteral and mucosal administration of a DNA prime MVA boost vaccine regimen. Submitted.

25. Biberfeld G, Mörner A, Liljeström P et al. Immunogenicity and protective efficacy of three different prime-boost HIV-1/SIV vaccine regimens in macaques. International Meeting of the Institute of Human Virology, 2003.

26. Sutter G, Moss B. Nonreplicating vaccinia vector efficiently expresses recombinant genes. PNAS 1992; 89: 10847-1085; 9

27. Sutter G, Moss B. Novel vaccinia vector derived from the host range restricted and highly attenuated MVA strain of vaccinia virus. Dev Biol Stand. 1995; 84: 195-200; 10

28. Moss B, Carroll MW, Wyatt LS, Bennink JR, Hirsch VM, Goldstein S, Elkins WR, Fuerst TR, Lifson JD, Piatak M, Restifo NP, Overwijk W, Chamberlain R, Rosenberg SA, Sutter G. Host range restricted, non-replicating vaccinia virus vectors as vaccine candidates. IN Novel strategies in design and production of vaccines Cohen S & Shafferman A Eds., Plenum Press , New York, 1996: 7-13

29. Meyer H, Sutter G, Mayr A. Mapping of deletions in the genome of the highly attenuated vaccinia virus MVA and their influence on virulence. J Gen Virol. 1991; 72: 1031-8

30. Antoine G, Scheiflinger F, Forner F, Falkner FG. The complete genomic sequence of the modified vaccinia Ankara strain: comparison with other orthopoxviruses. Virology. 1998; 244: 365-396

31. Durbin AP, Wyatt LS, Siew J, Moss B, Murphy BR. The immunogenicity and efficacy of intranasally or parenterally administered replication-deficient vaccinia-parainfluenza virus type 3 recombinants in rhesus monkeys. Vaccine. 1998; 16; 1324-30

32. Hirsch VM, Fuerst TR, Sutter G, Carroll MW, Yang LC, Goldstein S, Piatak Jr M, Elkins WR, Alvord G, Montefiori DC, Moss B, Lifson JD. Patterns of viral replication correlate with outcome in simian immunodeficiency virus (SIV)-infected macaques: effect of prior immunization with a trivalent SIV vaccine in modified vaccinia virus Ankara. J Virol. 1996; 70: 3741-52; 31

33. Seth A, Ourmanov I, Kuroda MJ, Schmitz JE, Carroll MW, Wyatt LS, Moss B, Forman MA, Hirsch VM, Letvin NL. Recombinant modified vaccinia virus Ankara-simian immunodeficiency virus gag pol elicits cytotoxic T lymphocytes in rhesus monkeys detected by a major histocompatibility complex class I/peptide tetramer. PNAS. 1998; 95: 10112-6

34. Hanke T, Samuel RV, Blanchard TJ, Neumann VC, Allen TM, Boyson JE, Sharpe SA, Cook N, Smith GL, Watkins DI, Cranage MP, McMichael AJ. Effective induction of simian immunodeficiency virus-specific cytotoxic T lymphocytes in macaques by using a multiepitope gene and DNA prime-modified vaccinia virus Ankara boost vaccination regimen. J Virol. 1999; 73: 7524-32

35. Hanke T, Blanchard TJ, Schneider J, Ogg GS, Tan R, Becker M, Gilbert SC, Hill AVS, Smith GL, McMichael A. Immunogenicities of intravenous and intramuscular administrations of modified vaccinia virus Ankara-based multi-CTL epitope vaccine for human immunodeficiency virus type 1 in mice. J Gen Virol. 1998; 79: 83-90

36. Belyakov IM, Wyatt LS, Ahlers JD, Earl P, Pendleton CD, Kelsall BL, Strober W, Moss B, Berzofsky JA. Induction of a mucosal cytotoxic T-lymphocyte response by intrarectal immunization with a replication-deficient recombinant vaccinia virus expressing human immunodeficiency virus 89.6 envelope protein. J Virol. 1998; 72: 8264-8272

37. Seth A, Ourmanov I, Schmitz JE, Kuroda MJ, Lifton MA, Nickerson CE, Wyatt L, Carroll M, Moss B, Venzon D, Letvin NL, Hirsch VM. Immunization with a Modified Vaccinia Virus Expressing Simian Immunodeficiency Virus (SIV) Gag-Pol Primes for an Anamnestic Gag-Specific Cytotoxic T-Lymphocyte Response and Is Associated with Reduction of Viremia after SIV Challenge. J Virol. 2000; 74: 2502-9

38. Ourmanov I, Brown CR, Moss B, Carroll M, Wyatt L, Pletneva L, Goldstein S, Venzon D, Hirsch VM. Comparative Efficacy of recombinant Modified Vaccinia Virus Ankara Expressing Simian Immunodeficiency Virus (SIV) Gag-Pol and/or Env in Macaques Challenged with Pathogenic SIV. J Virol. 2000; 74: 2740-51; 37

39. Ourmanov I, Bilska M, Hirsch VM, Montefiori DC. Recombinant Modified Vaccinia Virus Ankara Expressing the Surface gp120 of Simian Immunodeficiency Virus (SIV) Primes for a Rapid Neutralizing Antibody Response to SIV Infection in Macaques. J Virol. 2000; 74: 2960-5

40. Hirsch VM, Fuerst TR, Sutter G, Carroll MW, Yang LC, Goldstein S, Piatak Jr M, Elkins WR, Alvord G, Montefiori DC, Moss B, Lifson JD. Patterns of viral replication correlate with outcome in simian immunodeficiency virus (SIV)-infected macaques: effect of prior immunization with a trivalent SIV vaccine in modified vaccinia virus Ankara. J Virol. 1996; 70: 3741-52

41. Seth A, Ourmanov I, Kuroda MJ, Schmitz JE, Carroll MW, Wyatt LS, Moss B, Forman MA, Hirsch VM, Letvin NL. Recombinant modified vaccinia virus Ankara-simian immunodeficiency virus gag pol elicits cytotoxic T lymphocytes in rhesus monkeys detected by a major histocompatibility complex class I/peptide tetramer. PNAS. 1998; 95: 10112-6

42. Hanke T, Samuel RV, Blanchard TJ, Neumann VC, Allen TM, Boyson JE, Sharpe SA, Cook N, Smith GL, Watkins DI, Cranage MP, McMichael AJ. Effective induction of simian immunodeficiency virus-specific cytotoxic T lymphocytes in macaques by using a multiepitope gene and DNA prime-modified vaccinia virus Ankara boost vaccination regimen. J Virol. 1999; 73: 7524-32

43. Seth A, Ourmanov I, Schmitz JE, Kuroda MJ, Lifton MA, Nickerson CE, Wyatt L, Carroll M, Moss B, Venzon D, Letvin NL, Hirsch VM. Immunization with a Modified Vaccinia Virus Expressing Simian Immunodeficiency Virus (SIV) Gag-Pol Primes for an Anamnestic Gag-Specific Cytotoxic T-Lymphocyte Response and Is Associated with Reduction of Viremia after SIV Challenge. J Virol. 2000; 74: 2502-9

44. Ourmanov I, Brown CR, Moss B, Carroll M, Wyatt L, Pletneva L, Goldstein S, Venzon D, Hirsch VM. Comparative Efficacy of recombinant Modified Vaccinia Virus Ankara

Expressing Simian Immunodeficiency Virus (SIV) Gag-Pol and/or Env in Macaques Challenged with Pathogenic SIV. J Virol. 2000; 74: 2740-51

45. Ourmanov I, Bilska M, Hirsch VM, Montefiori DC. Recombinant Modified Vaccinia Virus Ankara Expressing the Surface gp120 of Simian Immunodeficiency Virus (SIV) Primes for a Rapid Neutralizing Antibody Response to SIV Infection in Macaques. J Virol. 2000; 74: 2960-5

46. Werner GT, Jentzsch U, Metzger E, Simon J. Studies on poxvirus infections in irradiated animals. Archives of Virology. 1980; 64: 247-56

47. Mayr A, Danner K. Vaccination against pox diseases under immunosuppressive conditions. 15th IABS Congress: Vaccinations in the developing countries, La Guadeloupe 1978. Develop Biol Standard. 1978; 41: 225-34

48. Wyatt LS, Earl PL, Eller LA, Moss B. Highly attenuated smallpox vaccine protects mice with and without immune deficiencies against pathogenic vaccinia virus challenge. Proc Natl Acad Sci U S A. 2004 Mar 30;101(13):4590-5

49. Stittelaar KJ, Kuiken T, de Swart RL, van Amerongen G, Vos HW, Niesters HG, van Schalkwijk P, van der Kwast T, Wyatt LS, Moss B, Osterhaus AD.Safety of modified vaccinia virus Ankara (MVA) in immune-suppressed macaques. Vaccine. 2001 Jun 14;19(27):3700-9

50. Mayr A, Hochstein-Mintzel V, Stickl H. Abstammung, Eigenschaften und Verwendung des attenuierten Vaccinia-Stammes. Infection. 1975; 3: 6-14; 41

51. Mayr A, Stickl H, Muller HK, Danner K, Singer H. Der Pockenimpfstamm MVA: Marker, genetische Struktur, Erfahrungen mit per parenteralen Schutzimpfung und Verhalten im abwehrgeschwachten Organismus. (The smallpox vaccination strain MVA: marker, genetic structure, experience gained with the parenteral vaccination and behavior in organisms with a debilitated defense mechanism). Zentralbl Bakteriol Parasitenkd Infektionskd Hyg Abt I Orig 1978; 167: 375-90

52. Mayr A, Danner K. Bedeutung von Tierpocken fur den Menschen nach Aufhebung der Pflichtimpfung gegen Pocken. Berl Muench Tieraerztl Wochenschr 1979; 92: 251-6

53. Moorthy VS, McConkey S, Roberts M, Gothard P, Arulanantham N, Degano P, Schneider J, Hannan C, Roy M, Gilbert SC, Peto TE, Hill AV. Safety of DNA and modified vaccinia virus Ankara vaccines against liver-stage P. falciparum malaria in nonimmune volunteers.Vaccine. 2003 May 16;21(17-18):1995-2002

54. McConkey SJ, Reece WH, Moorthy VS, Webster D, Dunachie S, Butcher G, Vuola JM, Blanchard TJ, Gothard P, Watkins K, Hannan CM, Everaere S, Brown K, Kester KE, Cummings J, Williams J, Heppner DG, Pathan A, Flanagan K, Arulanantham N, Roberts MT, Roy M, Smith GL, Schneider J, Peto T, Sinden RE, Gilbert SC, Hill AV. Enhanced T-cell immunogenicity of plasmid DNA vaccines boosted by recombinant modified vaccinia virus Ankara in humans. Nat Med. 2003 Jun;9(6):729-35

55. Gilbert SC, Schneider J, Hannan CM, Hu JT, Plebanski M, Sinden R, Hill AV. Enhanced CD8 T cell immunogenicity and protective efficacy in a mouse malaria model using a recombinant adenoviral vaccine in heterologous prime-boost immunisation regimes. Vaccine. 2002 Jan 15;20(7-8):1039-45

56. McCutchan FE, Hegerich PA, Brennan TP, Phanuphak P, Singharaj P, Jugsudee A, Berman PWm Gray AM, Fowler AK and Burke DS. Genetic variants of HIV-1 in Thailand. AIDS Res Hum Retrovir. 1992; 8:1887-95

57. McCutchan FE, Artenstein AW, Sanders-Buell E, Salminen MO, Carr JK, Mascola JR, Yu XF, Nelson KE, Khamboonruang C, Schmitt D, Kieny MP, McNeil JG and Burke DS. Diversity of the envelope glycoprotein among human immunodeficiency virus type 1 isolates of clade E from Asia and Africa. J Virol. 1996; 70: 3331-8; 47

58. Carr JK, Salminen MO, Koch C, Gotte D, Artenstein AW, Hegerich PA, St Louis D, Burke DS and McCutchan FE. 1996. Full length sequence and mosaic structure of a human immunodeficiency virus type 1 isolate from Thailand. J Virol. 1996; 70: 5935-43

59. Mascola JR, Louwagie J, McCutchan FE, et al. Two antigenically distinct subtypes of human immunodeficiency virus type 1: Viral genotype predicts neutralization serotype. J Infect Dis. 1994;169:48-54;49

60. VanCott TC, Bethke FR, Artenstein AW, et al. Serotyping international HIV-1 isolates by V3 peptides and whole gp160 proteins using BIAcore. Methods: A Companion to Methods Enzymol. 1994; 6: 188-98

61. Ferrari G, Humphrey W, McElrath MJ, et al. Clade B-based HIV-1 vaccines elicit crossclade cytotoxic T lymphocyte reactivities in uninfected volunteers. Proc Natl Acad Sci USA.1997; 94: 1396-1401; 51

62. Lynch JA, DeSouza M, Robb MD, Markowitz L, Nitayaphan S, Sapan CV, Mann DL, Birx DL, Cox JH. Cross-clade cytotoxic T cell response to HIV-1 proteins among HLA disparate North Americans and Thais. 1998. J. Infect. Dis. 178: 1040-1046

63. National AIDS Committee. National Plan for Prevention and Alleviation of HIV/AIDS, 1997-2001. Bangkok, 1997 [ISBN 974-7896-44-3]

64. Nitayaphan S, Brown AE. Preventive HIV vaccine development in Thailand. AIDS 12 (suppl B):S155-161, 1998

65. Thongcharoen P, et al. Future planning for HIV vaccine evaluation of Thai AIDS vaccine evaluation group (TAVEG) in Thailand. 12th World AIDS Conference, Geneva. Abstract 33222

66. Excler JL, Plotkin S. The prime-boost concept applied to HIV preventive vaccines. AIDS 1997; 11 Suppl A:S127-S13.

67. Sandstrom E, Wahren B, Hejdeman, B, et al. Multigene, multiclade HIV-1 plasmid DNA prime and MVA boost is safe and highly immunogenic in healthy human volunteers. International AIDS Vaccine 2006 Conference, Amsterdam.
